# Recent Advances and Challenges Toward Application of Fibers and Textiles in Integrated Photovoltaic Energy Storage Devices

**DOI:** 10.1007/s40820-022-01008-y

**Published:** 2023-01-20

**Authors:** Amjid Rafique, Isabel Ferreira, Ghulam Abbas, Ana Catarina Baptista

**Affiliations:** CENIMAT|I3N, Department of Materials Science, School of Science and Technology, NOVA University Lisbon, Campus de Caparica, 2829-516 Caparica, Portugal

**Keywords:** Flexible electronics, Electronic textiles, Energy harvesting, Supercapacitors, Photovoltaic devices

## Abstract

Flexible microelectronic devices have seen an increasing trend toward development of miniaturized, portable, and integrated devices as wearable electronics which have the requirement for being light weight, small in dimension, and suppleness. Traditional three-dimensional (3D) and two-dimensional (2D) electronics gadgets fail to effectively comply with these necessities owing to their stiffness and large weights. Investigations have come up with a new family of one-dimensional (1D) flexible and fiber-based electronic devices (FBEDs) comprising power storage, energy-scavenging, implantable sensing, and flexible displays gadgets. However, development and manufacturing are still a challenge owing to their small radius, flexibility, low weight, weave ability and integration in textile electronics. This paper will provide a detailed review on the importance of substrates in electronic devices, intrinsic property requirements, fabrication classification and applications in energy harvesting, energy storage and other flexible electronic devices. Fiber- and textile-based electronic devices for bulk/scalable fabrications, encapsulation, and testing are reviewed and presented future research ideas to enhance the commercialization of these fiber-based electronics devices.

## Highlights

- Compelling aspects of fiber- and textile-based flexible electrodes are reviewed in detail from the point of view of fabrication, properties, and devices performance.
- The advances of fibers and textile-based electrodes employed in flexible solar cells and flexible energy storage devices are discussed.
- The outlook and challenges in employing and developing textile-based flexible electrodes are highlighted.

## Graphical abstract

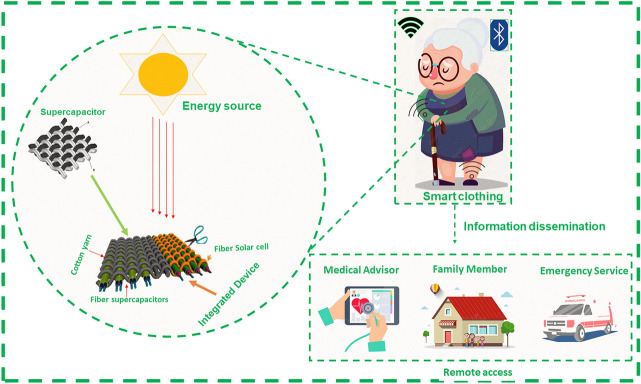

## Introduction

**Fig. 1:**
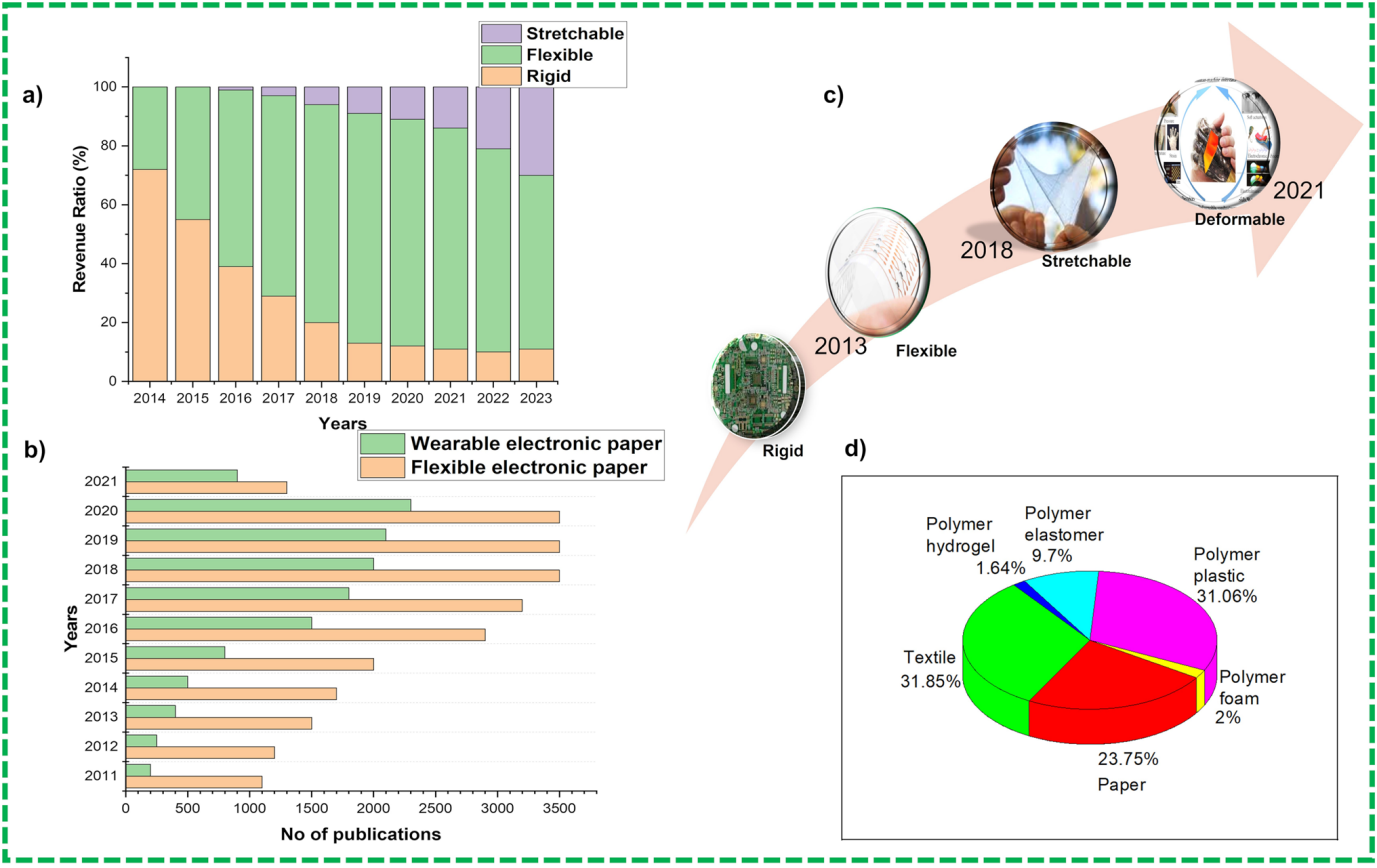
**a** world Revenue ratio of flexible electronics [6]. **b** Number of publications on flexible and wearable electronic devices during peri 2011–2021 period. **c** Evolution of fabrication of flexible electronics. **d** Comparative allocation of number of publications for different polymer and flexible substrates during 2011–2021 period and data are also indexed in Web of Science in December 2021

The Internet of Thing (IoT) has reached a huge impact on self-recognition, real-time access to information for better communication and data-driven decision-making. The demand for flexible, portable, and wearable devices (e.g., smart electronics devices, artificial skins and implantable) has increased in both civilian as well as military domains which requiring advanced electronics devices for managing to better decision making [1]. The increasing trends for flexible, portable, low-cost, and lightweight wearable electronics attracted academic interests to compliance with future challenges for the fabrication of the next generation FBEDs which highlight the importance of the technical advancements in terms of potential applications and performance of the devices as well as improvement in the quality of life and significant economic growth [2]. Figure 1a [3] shows the tendency of stretchable electronics market with a cumulated aggregate growth rate (CAGR) of 25.29% in the forecast period of 2020–2026, and the market value is expected to be worth USD 2981.2 million by end of 2026 [4]. This becomes a consequence of research and development (R&D) efforts in recent past with proof of increasing numbers of publications in the flexible electronic devices field as shown in Fig. 1b. Flexible devices have experienced a considerable growth from plain electronic devices to flexible devices (2014) and to stretchable electronics devices (2019), with present attention moving to foldable and deformable electronic devices as depicted in Fig. 1c [5]. These have also been associated with different flexible substrates as demonstrated in Fig. 1d where paper, textiles and polymers plastics get the major share above 80% of the reports.

Modern technology and devices, on one hand, enable us to better explore ourselves in terms of memory, disease, learning and aging [7, 8]. On the other hand, we are increasing our social connection with other people heavily relying upon the flexible and portable electronic devices such as laptops and smartphones [9]. These trends in turn boosted the development of the wearable electronic devices (WEDs) to comply with the tasks associated with fast, accurate, direct, and convenient processing of information [8]. For example, smart watches help us to track the movement and communicate to us when we do exercise, instead of holding other heavy electronic devices. These WEDs are in close contact with human bodies which are smooth and portable. During these contacts with body movements, they are subjected to different stress levels and deformations, so these devices must be stretchable and flexible enough to comply with these deformations. Moreover, to avoid the normal working and to ensure the comfort of the wearer, they must be lightweight. Traditional three-dimensional (3D) and two-dimensional (2D) devices failed to efficiently comply with above-mentioned requirements owing to their rigidity and bulkiness.

Moreover, flexible FBEDs, embracing one-dimensional (1D) assembly with modest curvature from 10 to 100 µm, have allured extensive interest toward its application in the wearable electronics area. These devices can be fabricated in different configurations such as interlacing, twisting and coaxial. Being fiber-based devices, they are light in weight and flexible and can adopt and sustain different deformation stresses such as bending deformation, stretching and distortion deformation. It is important to note that these 1D fiber-shaped devices can easily be weaved into flexible, breathable, and deformable textiles which facilitate practical applications of these devices. Hence, research efforts have focused on increasing their performance to exploit in the textile-based substrate for further growth of stretchable, self-healing, or shape-memory functionalities [8]. Self-powering devices by fabricating energy harvesting devices integrated with energy storage devices or energy storage devices integrated sensors have been demonstrated [10]. These advancements have motivated and inspired the tech industry like wearable electronic and clothing industry to exploit the well-established traditional textile technology for weaving and developing these fiber-based electronic devices (FBEDs). One example of such devices is a Jacquard smart jacket co-launched by Google and Levi’s which has integrated fiber-shaped sensors [11] but with a limited functionality such as reminding the wearers about keeping the smartphone with them.

**Fig. 2:**
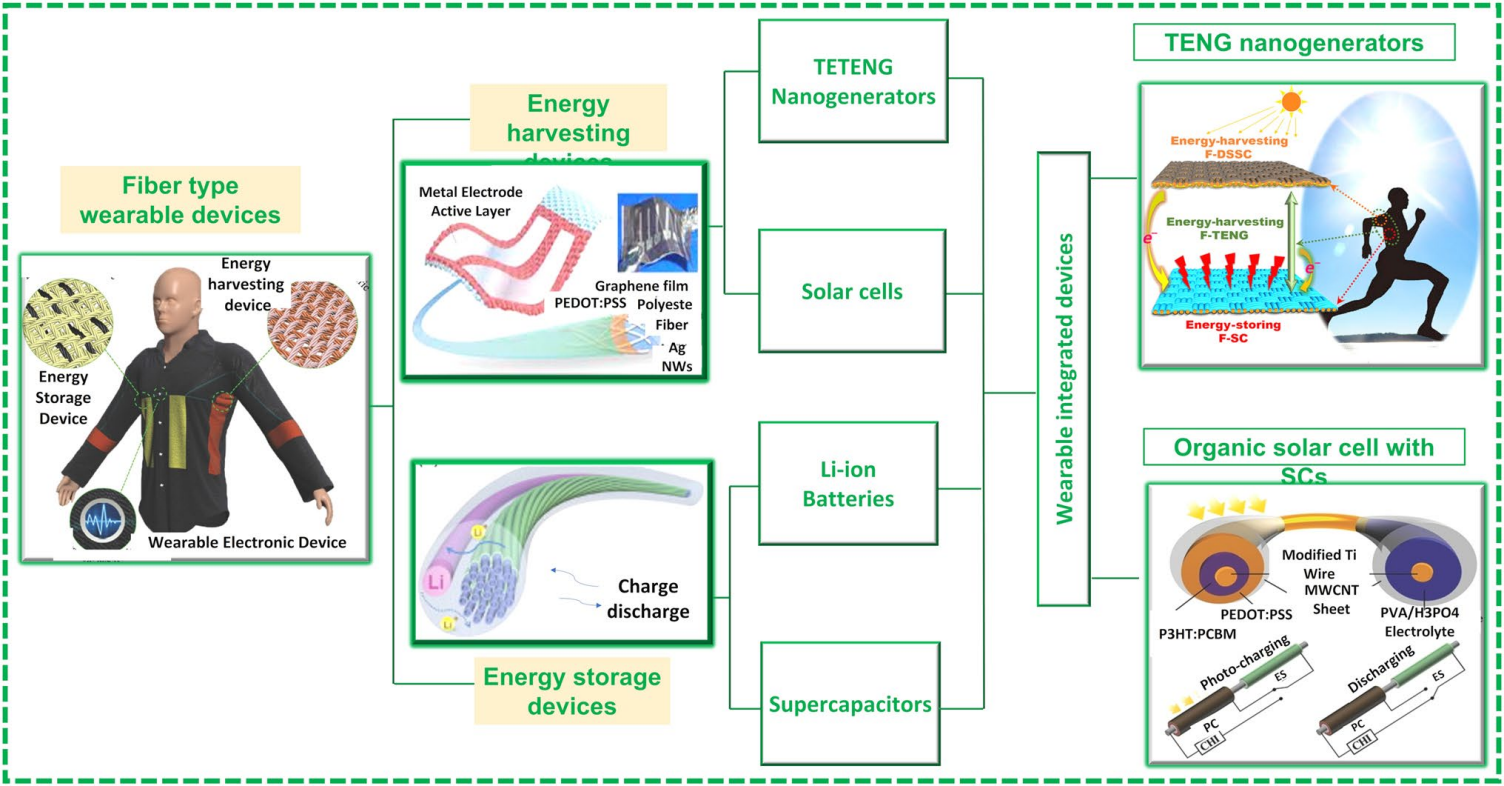
Graphic representation of the key features of this review article: Reprinted with permission from Refs. [13, 22–25]

Further efforts are required to manufacture advanced textile-based electronic devices integrating more functionalities and simultaneously be integrated in the textile fibers. The reported FBEDs are classified mainly into four categories: (1) textile-based energy-generating devices which transform the different types of energy, such as solar and mechanical energy, into electrical energy. These devices are mainly comprised of flexible solar cells, piezoelectric nanogenerator and triboelectric nanogenerators [8]. Fiber-shaped solar cells, piezoelectric and triboelectric nanogenerators are the most researched devices that can be integrated into textile effectively and are primarily reviewed in this paper. (2) Textile-based energy storage devices have been extensively investigated to save energy and dispense this power to other wearable electronic devices where required. The reported textile-based energy storage devices include supercapacitors (SCs) [12], flexible lithium-on batteries [13], Li–S batteries [14], Li–air batteries [15], sodium-ion batteries [16], Zn-ion batteries [17] and silver–zinc batteries [18]. Among these reported devices, SCs are the most cited ones owing to its easy fabrication, long cyclic life, and high-power throughput. Lithium-ion batteries have also been manufactured into textile-based energy storage devices and will be considered in this review. (3) The development of light emitting electronic devices for various applications such as flexible display, phototherapy, and illumination. Based on their working mechanisms, these devices are reported as electroluminescence, sonoluminescence, thermoluminescence, mechanoluminescence and chemiluminescence [8]. Amid these reported devices, electroluminescent devices are the most extensive studied devices owing to its good controllability and operated by direct current (DC) [19] and alternating current (AC) [20] methods. (4) Fiber-based sensors having excellent potential for applications in flexible electronics and implantable medical devices for physical health and fitness monitoring. Recently, they have also been explored for medical diagnostics, especially for elderly populations. Thanks to their 1D configuration, fiber-based sensors can be easily implanted and mounted on the human body with little or no damage, while providing multi-tasking when integrated into a small electronic unit [21]. These sensors worked through physical processes based on conducting fibers, optical fibers and chemical ligands exploiting chemical processes [8]. Here, focus is on energy storage and energy harvesting devices and their integration with textile for the development of E-textiles, as shown in Fig. 2.

Although there is clear progress in the development and manufacturing of textile fibers and electronic devices, we are still far away from their real applications due to many constrains. One of the most important is their often-deteriorating performance as their length increases together with scalability need to be addressed developing commercial-ready fiber-shaped devices [26]. Another key to the application of FBEDs is the connections to electronic components that must be compatible with a simple and efficient manufacturing process. Protection and stability of the electronic devices are other two vital issues to deal with as numerous FBEDs include toxic and/or volatile compounds such as Pb [27] and acetonitrile [28]. In that regard, the use of these toxic or flammable chemicals during fabrication of these devices need to be abandoned while developing a consistent protective encapsulation technology for its circumvention.

In this review, we have thoroughly reviewed the evolution of substrates starting from rigid to flexible and stretchable substrates, devices fabrications from 2D to 1D configurations which is suitable for E-textiles. we have also discussed in detail the challenges encountered by textile-based electronic devices through all the manufacturing stages, from single fabricated devices to constantly scalable manufacturing, device encapsulation, characterization, testing, and exploration of potential applications. Several authors reviewed [8, 29–31] the progress of application of fiber and textile fibers as flexible substrate in the electronic devices in the past. We have designed our review paper structure around following points: (1) Evolution of substrate from rigid to flexible substrates in realization of textile-based electronics: (2) Progress in material development for E-textiles: (3) Standardization of performance evaluation of the electronic devices: (4) Progress and challenges faced toward industrialization and large-scale production of the E-textiles. This review paper complements previous review studies and provide an updated progress of fiber and textile application in E-textiles. We have also presented the summary of published papers and patents in the field with reference to its impact on the scientific community and society. New ideas and future directions to boost their performance and commercialization are also discussed. We aim that this review provides a comprehensive understanding of prospects and challenges of each single stage and supports realizing new advancements in the research and application of fiber- and textile-based flexible electronic devices.

## Advantages of Fiber and Textile-Based Substrate for Wearable Electronic Devices

The development of fiber and textile-based electronics requires consideration over many different attributes ranging from technology, active material, working mechanisms, extent of variability in flexibility, mode of applications with reference to advantages, disadvantages, and limitations. One of the key factors in designing FBEDs is the choice of substrate which has an exceptional blend of performance features, making it appropriate for the specific application. Electronic devices comprising flexible substrates allow various degrees of flexibility namely, bendability, elastic stretchability, rollability, and permanent slandering. In addition to flexibility, these substrates offer maintainability of other key characteristics such as excellent adhesion of active material during compression, twisting and stretching stresses. Compared to rigid substrates, fiber and textile-based substrates in flexible electronic devices offer numerous advantages, e.g., light in weight, thin, high mechanical resistance, and cost-effectiveness. Application of these fiber and textile-based substrate in biomedical and consumer electronics is forecasted to have great potential for market growth. With the flexible electronic market value being projected to grow to USD 775 million during the 2018–2023 period, at a CAGR (compound annual growth rate) of 14.0% [32].

In the processing of design and manufacturing of the FBEDs, a key element is the selection of a substrate which possesses stretchability and mechanical flexibility. The detail list of the key variables comprises numerous physicochemical attributes, namely thermal stability (in case of extreme weather conditions), chemical stability, excellent adhesion of the coating, surface evenness, optical clarity (e.g., solar cells), and permeability and water repellency. The applications where wearable electronic devices need to contact with the human body, then biocompatibility and bioresorbable attributes of the substrate need to be selected carefully for long-term sustainability. Some of the key attributes of flexible substrates include.

### Mechanical Stability

The flexible wearable devices are subjected to various mechanical stresses in practical life; thus, it is a requirement for a flexible electronic device to sustain those mechanical stresses. The term flexibility refers to the ability to sustain various mechanical stresses while maintaining its functional properties and performance parameters under those stresses. The target flexibility for electronic devices is quite diverse owing to its broad range of applications. For example, in implanted sensors/communication devices which are mounted/integrated on human skin or other human organs, it is anticipated the need to cope with high stress. However, in other applications small recurring stress cycles or modest one-time strain are expected. In normal conditions, flexible substrates demonstrate essentially ductile-plastic behavior that protects mechanical failure beyond the elastic limit. As the ductility of the substrate is tuned to increase, the strain limit also increases in parallel which results in enhanced flexibility and elongation. Hence, the representative parameter which deals with the mechanical flexibility of the substrate is Young’s Modulus (YM) and measures the stiffness of the materials. YM is a ratio between the stress (*σ*) to strain (*ε*) in the linear elastic system of a uniaxial distortion. The low modulus material represents wide stress array which reveals desirable features without deteriorating the mechanical and electrical behavior. Similarly, through downscaling of the dimensions of the polymer-based flexible substrates, such as Polyethylene terephthalate (PET) and Polyimide (PI), the modulus can be reduced because the thickness of the film and modulus of strained film are inversely proportional to each other according to the theoretical model:

1 $$E = 54.872 \times h^{0.226}$$

where *h* is the thickness of the film [33].

**Fig. 3:**
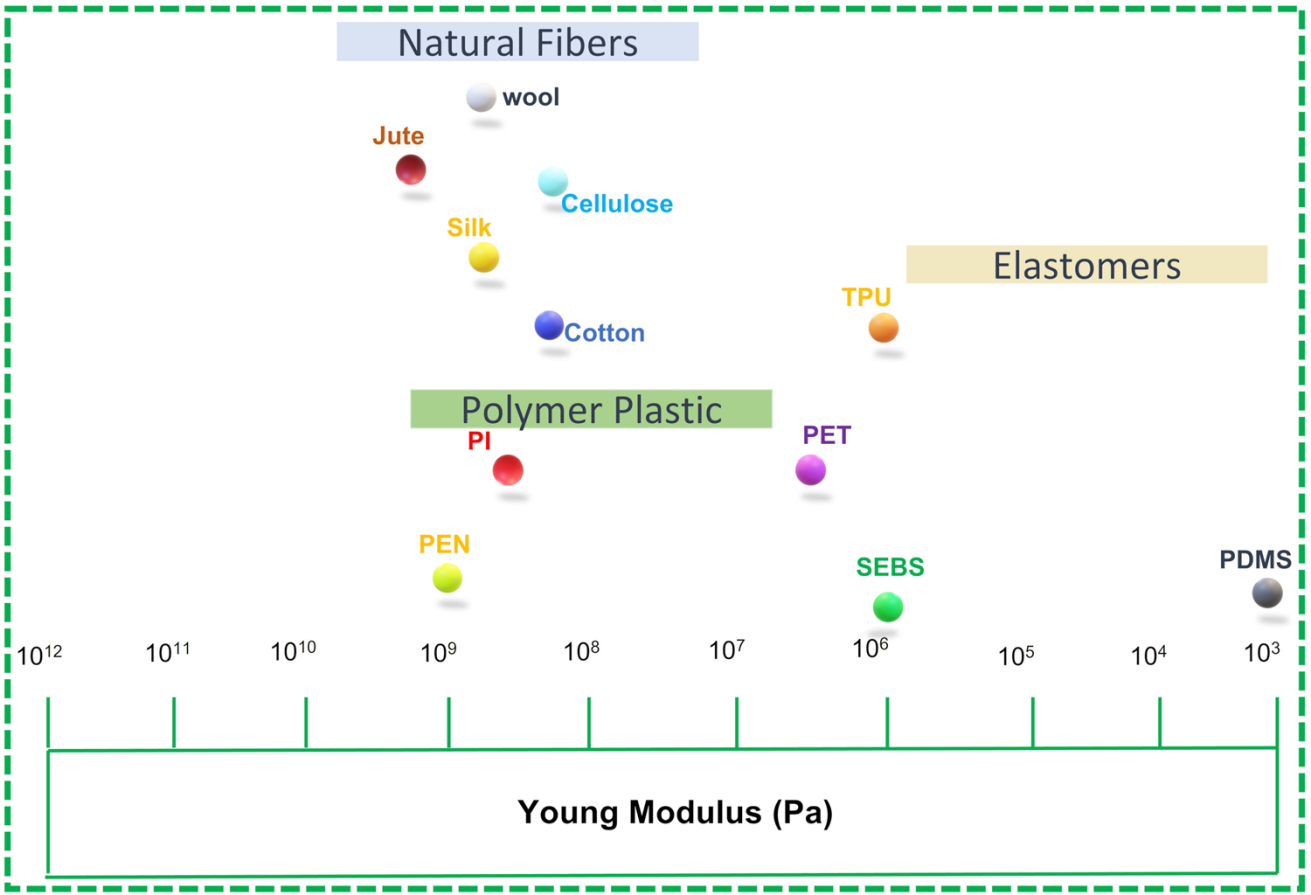
Young’s Modulus of materials for flexible and wearable electronics [34, 35]

Figure 3 demonstrates the relationship between mechanical factors and flexible substrates for various ranges of YM of some traditional substrates and flexible materials for wearable electronic devices. Moreover, during processing and application of flexible and stretchable substrates as electrodes, the final strain experienced by the electrodes is directly proportional to YM of the material encapsulating the active material. Thus, for flexible and wearable electronics applications, the substrate must exhibit lower value of YM, sustain deformation stresses, and retain high stretchability. For instance, polydimethylsiloxane (PDMS) with YM = 360–870 kPa displays outstanding performance when subject to mechanical deformation conditions, which empowers its application in flexible and wearable electronics.

### Stretchability

In order to exploit flexible substrates in wearable and implantable electronics, it is required for the material to have excellent stretchability to guarantee outstanding performance of the electrodes. The term stretchability, in qualitative terms, is defined as the ability to sustain its properties, e.g., conductivity, under severe mechanical stresses. In quantitative terms, it is defined as the critical stress state of the material at which its behavior changed, for instance from a conductive material to a non-conductive material. In specific terms, stretchability is the ability of materials in flexible and wearable systems, to sustain stress induced by external stimulators such as motion of the body or 100% elastic deformation range. For wearable applications, materials with intrinsically low ductile-plastic behaviors prevent mechanical cracks beyond elastic limits and exhibit stretchability to required value while allowing for out-of-plan distortion. There are two main identified techniques for the fabrication of stretchable electronics: (1) structural modification of the substrate, which includes numerous patterning structures such as serpentine, mesh-types, merged structures, mogul pattern and fractal fabricated by depositing on polymeric substrates in order to sustain excellent conductive characteristics even under mechanical stresses. (2) Exploiting intrinsically stretchable substrates and (3) incorporation of filler materials in the polymers system by either systematic manners or random technique. On the latter approach due to the non-stretchable nature of numerous fillers, several small dimensional nanomaterials, such 1D or hybrid structures, are fabricated to increase the stretchability, while upholding transparency of the flexible substrates [7]. Moreover, 2D materials with exceptional intrinsic conductive properties and facile scalable synthesis technique can be exploited as potential candidate for future electronic devices.

### Adhesion

Adhesion is the propensity of unalike nanoparticles or surfaces to stick to one another due to different forces namely intermolecular forces responsible for numerous functions of several kinds. For instance, labels and tacky tape fall into different classes of chemical adhesion, diffusive adhesion, and a dispersive adhesion. Subject to different chemical and physical properties of the substrates and metals, poor adhesion is obtained mainly for thin film electrodes deposited on flexible substrates. In the flexible and wearable electronics, outstanding adhesion of deposited material or thin films are critical because of external stimulators provide abrasion energy which might cause pilling of some poorly adhered thin films or materials and render the electronic devices impracticable. The leading factor for the adhered material is usually substrate surface energy which comes from polar or dispersive characteristics of the substrates. A good adhesion is obtained when the substrate demonstrates a surface energy of 7–10 dynes cm^−2^ higher than the deposited materials and it is normally in the range of 34–36 dynes cm^−2^ [36]_._ Amid other factors, surface pollution and low activation of the substrate are some of the root causes for low activation energy. To achieve an excellent adhesion of nano-particles between conductive materials and substrates, primarily two methods are exploited including construction of chemical bond between the metal and physical interlocking. For implantable devices, where good adhesion of the active material with the active substrate is important chemical bond adhesion is not stable and only physical methods offer good adhesion which involves the development of a transition layer sandwiched between metal and substrate in order to obtain good adhesion and stability of the devices. Excellent adhesion properties are critical to realize a steady performance of the electrodes, and it becomes more important in the case of wearable electronics because during operation they are subjected to bending stresses and there are chances of pilling of active material from the substrate surface. In addition, for encapsulation of conductive material, adhesion between electrode and encapsulation coating is critical with inevitability of double interfacial coating design for good electrode encapsulation [7].

### Permeability

During use, wearable and flexible electronic device are surrounded by complex environmental constituents such as gases, liquids and other chemical molecules that have tendency to penetrate in the structure of the flexible substrate and compromise the performance and sensitivity of the devices. Water droplets which may become entrapped under the surface of the devices, leading to prolonged exposure of the device to this moisture, may deteriorate the device performance and cause some infection to the user’s skin, which may induce severe pain and delayed healing of the body parts. This problem can be avoided by using substrates which are impermeable or selectively permeable to biological environmental constituents,’ such as liquids, gases, proteins, and other molecular substances. Substrates like PDMS and PI are considered excellent flexible substrates for biomedical applications, due their good permeability to organic thinners, small lipophilic particles (PDMS), high durability, and good electrical properties for longer time in aqueous and saline solutions [37]. Stretchable substrates normally comprise of long chain molecules and have large pores size as compared to molecules, which allow good permeability to aqueous molecules and oxygen. This is also the case with breathability which is a critical parameter in case of wearable systems because it allows long lasting wearability and excellent adhesion of wearable and flexible systems during perspiration. The high permeability substrates also help to avoid potential risks to skin infections by allowing water and gases to pass through sweat gland ducts. Recent literature shows that by careful modeling and design of the substrates, porosity, pore size of the material can be successfully controlled to achieve selective permeability, such as in fibers and textile substrates [38].

### Water Repellency

Water repellency is the term described as the capability or characteristic of the substrate to repel wetting of the substrate. As discussed above, water vapor entrapped under wearable devices compromise the device performance and its sensitivity and may cause skin inflammation. In case of wearable systems, it is mandatory to make some surface modification of the substrate before its application to wearable systems. Chemical or structural modifications are made to change its affinity to water and introduction of moisture management properties to remain unaffected from moisture, sweating, washing, and showering. Recently, numerous approaches have been reported in the literature to exploit water affinity of the substrate, which has helped to enhance water repellency and introduce hydrophobicity in the material. This has been achieved by introducing filler elastomers such as poly(styrene–isoprene–styrene) in sensors, which shows better results as compared to PDMS, which has its hermeticity deteriorate for long exposure to water vapor [39]. Moreover, device sensitivity and performance can be tailored by making the substrate super hydrophobic, and this also helps to minimize its indissoluble affect during device fabrication and assembly. During the deposition of the hydrophilic coatings on the substrate, surface treatment must be done beforehand to increase surface functionality, although this may lead to external moisture penetration. Therefore, the hydrophobic property of the substrates must be carefully considered to design good water repellent substrates.

### Softness

The softness of the material, normally represented by the YM, is very important especially in biomedical applications where both stretchability and softness are critical to make sure mechanical compatibility among the human body tissues and the electrode material, because any mismatch between these properties may result in issues with tissues or inflammation in the skin. Therefore, simultaneous realization of these two properties is highly recommended in biocompatible medical devices. Softness is the key element while designing wearable biomedical devices, when compared to stretchability, which is not fully discussed in literature, and extensive research is required in this attribute to investigate tissue interactions at cell level to design an implantable device which has no harmful effects on human tissues. In addition to the substrate’s softness, conductivity of the implantable device is also very important and various approaches have exploited to enhance the softness of the active layer of the electrodes which plays a key role in biomedical field, especially during surgery, particularly for deep brain stimulation where electrodes need to be inserted in the brain. To avoid damage or to control the tissue damage during insertion and removal of the materials, stretchable, removable rigid substrates and shape-memory polymer substrates can be exploited for this purpose.

### Chemical Stability

Chemical stability of the substrate is very important because during the electronic device’s fabrication process, they are subject to different solvents and chemicals, which may cause damage to flexible substrates. Hence, substrates must be compatible with different chemicals such as acidic, basic, and other solvents. However, while thin film encapsulation coating on substrates is sometime exploited to protect flexible substrates from chemical damages, nevertheless, it is not practical. Typically, a hard coating on the substrate can offer better protection against permeation by atmospheric constituents [40].

### Thermal Stability

Thermal stability of the substrate is very important, especially for extreme conditions electronic devices, and this may result in more process steps in device fabrication. The thermal stability parameters optimization is ruled by |ΔCTE·Δ*T*|≤ 0.1 0.3%, where ΔCTE and Δ*T* are the coefficient variances of the thermal expansion of substrate, thin film of the devices and temperature excursion during fabrication, respectively [41]. This temperature excursion between thin film of the device and flexible substrate may lead to convex bending of the devices which may result in cracking and delamination of the film during fabrication of devices. Therefore, to avoid this thermal cracking problem under extreme temperature conditions, the design and development of the thin film must be robust to thermal expansion to evade the mismatches.

### Biocompatibility

The term biocompatibility is normally defining the property of the material being well-suited to living tissues and these materials do not yield lethal or immunological issues when exposed to human body or fluids. For application of wearable electronics in the medical field, biocompatibility of the material and substrate is considered a critical element because it will generate intimate connotation with the biological interfaces. Biocompatibility is compromised due to various factors such as chemical composition, pH of the solution and surface charge. There are numerous substrates which have good biocompatibility such as PDMS, cellulose substrate and silk-based textiles. In addition to these materials, some other substrate materials can also be exploited as biocompatible materials such hard gelatin, starches, and caramelized glucose. The other self-assembling polymers such as cellulose, silk and chitin that offers similar hierarchical configuration with natural bio-repetitive arrangement are also potential substrates for flexible electronics. In the recent trends, wearable health monitoring applications use biomass materials such as bacterial cellulose, which is reported as biocompatible fibers [42]. Another approach to fabricate a biocompatible substrate is to encapsulate flexible devices with biocompatible coatings such as PU, Eco flex and PDMS [7].

### Bioresorbability

Bioresorbable materials also known as biodegradable materials are materials that eradicate ecological, chemical, or physical harms by decomposing or dissolving themselves, without any environmental impact and residue toxic pollutants inside the human body. The very concept was given in 2009 and after that numerous efforts have been made for incorporating these biocompatible materials in wearable systems and exploited to improve conformability of the devices. Roger et al. [43] described electrocorticography and heart electrophysiological devices on mechanical compliant flexible substrates. Silk is recently reported as the green substrate which enhances device conformability when implanted on the surface or within the human brain [7].

## Applications of Flexible Substrates for Electronic Devices

In the following section, fiber and textile-based applications will be discussed mainly in two fields fiber-shaped energy harvesting and fiber-shaped energy storage devices, both from materials and application’s perspective. Thanks to fiber and textile-based substrate, flexible electronics can work where current electronic devices have limitations. Therefore, it is very important to highlight the advantages of these flexible substrates to make further improvement and overlook the irrelevant shortcomings.

### Energy Harvesting Devices

#### From Solar Energy

Conventional classification of solar cells is: (1) silicon-based, also known as first-generation photovoltaics such as crystalline silicon; (2) thin-film photovoltaic devices, known as second generation photovoltaics such as amorphous silicon, copper indium gallium selenide (CIGS), cadmium telluride and (3) recent technologies for energy harvesting, such as dye-sensitized solar cells (DSSCs), organic photovoltaics (OPVs) and Perovskite solar cells (PeSCs) [8, 44]. Currently, silicon-based solar cells have captured a major share of the market due to its excellent Photo Conversion Efficiency (PCE), around 20%, and remain dominant for the last 20 years [45]. Nowadays, researchers are focusing on third-generation solar cells, which are more flexible, cost-effective, and with different attributes that can easily be customized, such as translucency, shade/structure control as linked to traditional silicon-based solar cells.

**Fig. 4:**
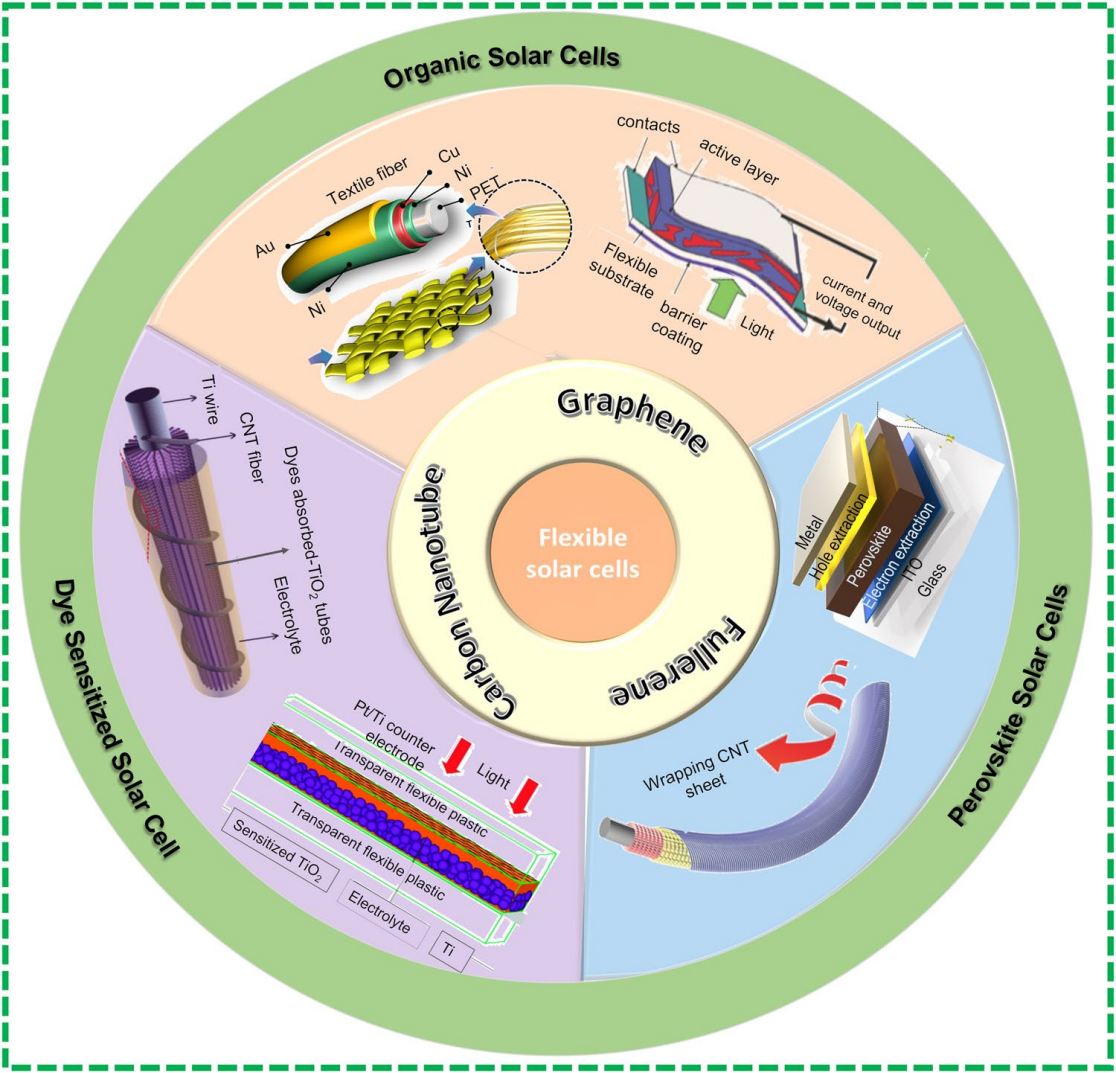
Schematic view of solar energy harvesters. Reprinted with permission from Refs. [27, 49–54]

Fiber-based solar cells are then a recent concept and are composed of two electrodes, one coated with electron transport materials (ETM) such as TiO_2_ and ZnO, photoactive reagents such as dye N719 for dye-sensitized solar cells, P3HT:PC61BM (poly(3-hexylthiophene) [6,6]:-phenyl-C61-butyric acid methyl ester) for polymer-based solar cells (PSCs) and PeSCs for Perovskite solar cells and HTM (hole transport material) such as PEDOT: PSS and CuI in succession [46]. Effective ETM and HTM hold suitable energy bands to extract electrons at fiber/ETM interface and holes at another fiber/HTM interface of the electrode. The photo-conversion efficiency and time are two parameters employed to assess the performance of fiber-based solar cells like conventional solar cells. Figure 4 summarizes the developments in the DSSCs [28] field PSCs [47], PeSCs [48].

##### Fiber-Shaped Dye-Sensitized Solar Cells (FSDSSCs)

FDSSCs were first proposed by Pichot et al. [55] in 2000. The FDSSCs devices attracted significant attention of researchers and companies owing to its potential for large-scale production at low cost, huge potential for commercial applications, such as integrated devices and biomedical applications [56] and due to its high deformability and fabrication in curve shape, which make it an appropriate nominee for mobile and flexible electronics applications. These types of devices normally required polyethylene terephthalate (PET) and polyethylene naphthalate (PEN) substrates. The choice of active material is made based on their band energy level (BEL). For DSSCs, TiO_2_, SnO_2_ and ZnO have been chosen because of their wide bandgap properties [57].

Since PET and PEN degrade in the temperature range of 120–150 °C, it limits the processing and applications of FDSSCs. The working electrodes must be prepared without binders and without annealing. These binder free layers of active materials result in cracks forming in the thin films during the drying process which increase the risk of exposure of electrolyte to substrate. Secondly, passing the calcination process during the fabrication process makes organic particles remain on the active layer surface which weakens electron transportation through the electrodes. Consequently, the cracks and residual particles present on the surface of the active layer degrade the photo-conversion performance and efficiency of the device and its stability [58].

**Fig. 5:**
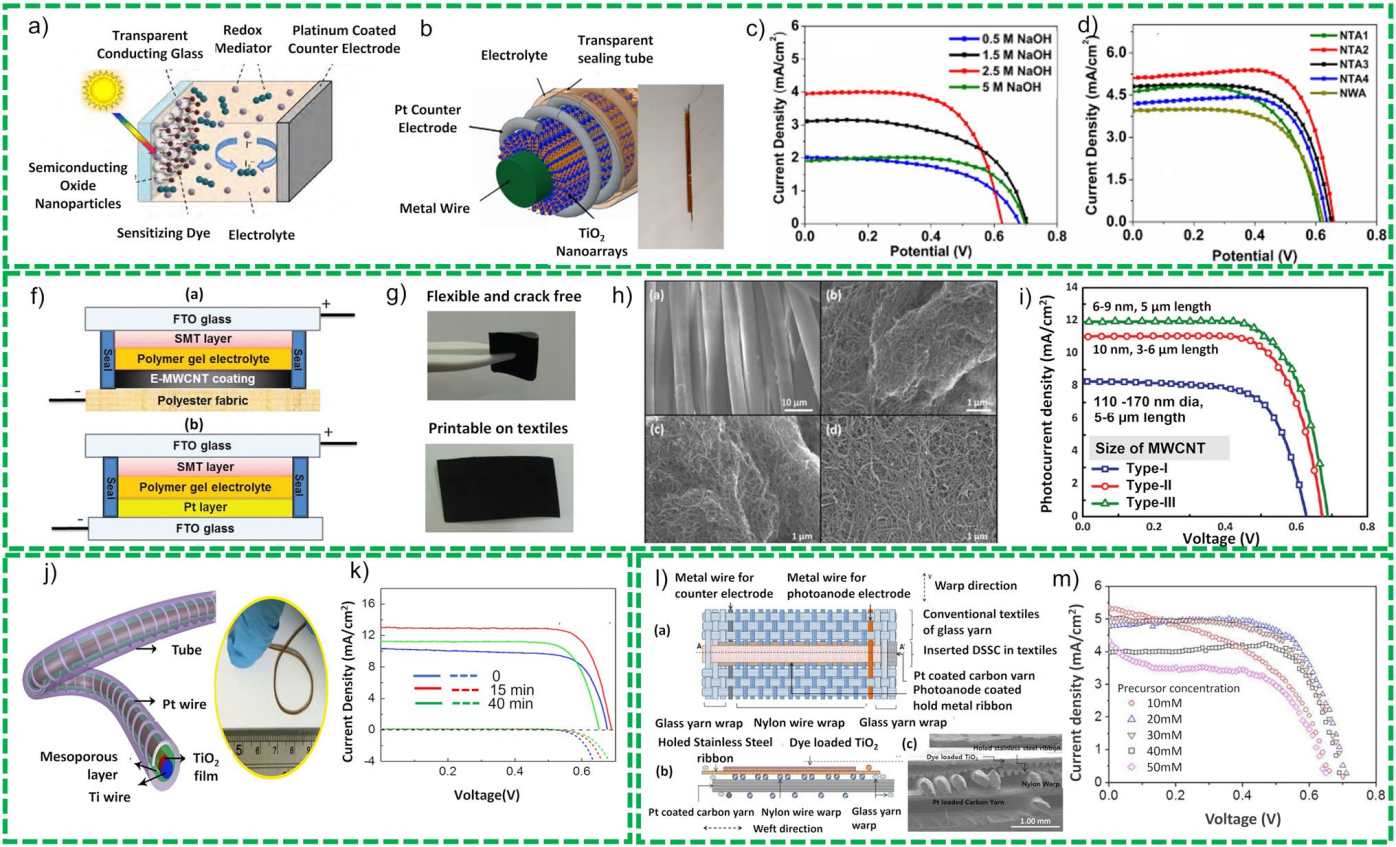
**a** Construction and working principle of DSSC, **b** graphic representation of device and pictorial demonstration of fabricated device, growth mechanism of the novel hierarchical photoanode and performance optimization, **c**–**e** optimization of base concentrations, optimization of H_2_SO_4_ for the NTA, comparison of nano-tree TiO_2_ and Ag deposited nano-tree TiO_2_. Reprinted with permission from Ref. [60]. **f** Flowchart demonstration of synthesis of E-MWCNT conducting paste and fabrication of an E-MWCNT fabric electrode, **g** diagram of assembly proposed DSSC and conventional DSSC, and Essential features of an E-MWCNT coated polyester fabric electrode, **h** FESEM images of polyester fabric with hydrophobic surface finish, Laccase suspended E-MWCNT and Gox suspended E-MWCNT, **i** electrical resistance of the E-MWCNT-coated fabric with the bending cycle, scotch Tape test performed on E-MWCNT coated fabric, clear surface of Scotch Tape and bending stability. Reprinted with permission from Ref. [61]. **j** Schematic illustration of DSSC fabrication with GCF counter electrode, **k** efficiency and PEC comparison with different thickness and bending radius counter electrodes. Reprinted with permission from Ref. [62]. **l** Schematic representation of the structure of the inserted dye-sensitized solar cells (DSSCs) in the textile in planar view and cross-sectional view of the AA′ section shown in (**a**) SEM images of the AA′ section in (**a**), showing the cross-sectional view of the inserted DSSC in the textile, **m** *J–V* features for thickness of layers and concentrations of precursors. Reprinted with permission from Ref. [64]

Fiber and textile-based substrates offer the advantages that they are stable and sustain high temperature calcination treatment to remove these organic residual particles from the active material paste. On the other hand, they offer more flexibility compared to the polymer-based substrates. The first planar-shaped DSSCs were presented by Xu et al. [59] in which cotton textile-based counter electrodes for PSDSSCs were used to substitute fluorine-doped tin oxide (FTO) in the solar cell devices. The working electrode is comprised of electroless plating of Ni at low temperature followed by in situ polymerization of polypyrrole on Ni-plated textile substrate. Although the device has demonstrated excellent stability, key performance indicators, such short circuit current (*J*_sc_), open circuit voltage (*V*_oc_) and fill factor (ff) for textile-based counter electrode, are still on the lower than required. Choudhury et al. [60] fabricated a device using Pt wire as a flexible substrate covered with TiO_2_ as active material that is synthesized using hydrothermal method. The active material is nanowires arrays (NWAs) that are doped with Ag nanoparticle using plasmonic resonance technique to absorb wider range of UV–visible light and results in higher PCE as shown in Fig. 5b. The device has demonstrated a PEC 4% as compared to pristine 3.15%. Arbab et al. [61], Song et al. [62] and Memon et al. [63] fabricated the similar configuration of textile-based DSSCs in which they have used MWCNT-coated textiles substrates as counter electrodes. The devices have demonstrated photo-conversion efficiency of 5.7%, 7.41% and 6.26%, respectively, as compared to platinum-based counter electrode with 7.26%, as demonstrated in Fig. 5f–k. Yun et al. [64] presented a novel technique for the integration of DSSC electrodes into textiles using the knitting manufacturing method of textiles. In this device, the titania-based dye-loaded porous active material is exploited for the fabrication of the photoanode. Platinum nanoparticle-coated yarn is used as the counter electrode. The final fabricated devices are integrated into textile using sewing techniques as displayed in Fig. 5l and m. The device has shown a photo-conversion efficiency of 2.63%. Although this demonstrates an innovative technique for integrating the DSSCs into textiles structure, the fabricated devices are still not completely textile-based owing to the use of metal ribbon and metal wires as part of the devices.

Opwis et al. [65] developed a textile substate-based DSSC on weaved fabric employing glass fibers. Initially, they loaded it with polyamide (PA) exploiting R2R fabrication technique to prepare a uniform and smooth surface for the photoanode layer, along with titania deposition using the screen-printing technique as active material for the solar cell. The problem with such fabrication technique is that it needs calcination at high temperatures which increases the cost of the development. There is a need to search and investigate for fabrication techniques for large-scale manufacturing at low-cost and bulk production for commercial applications.

##### Flexible Organic Photovoltaics

Organic solar cells (OSCs) are lightweight, have inherent flexibility, can be applied in large-area block printing assembly and are of low cost, which make them potential clean energy solutions. Conjugate polymers are normally employed as the electron donor and hole conductor in the active material layer of the OSCs, and it is expected that traditional development steps, e.g., doctor blade and roll-to-roll can be exploited to make wide area, low-cost OSCs on flexible substrate.

**Fig. 6:**
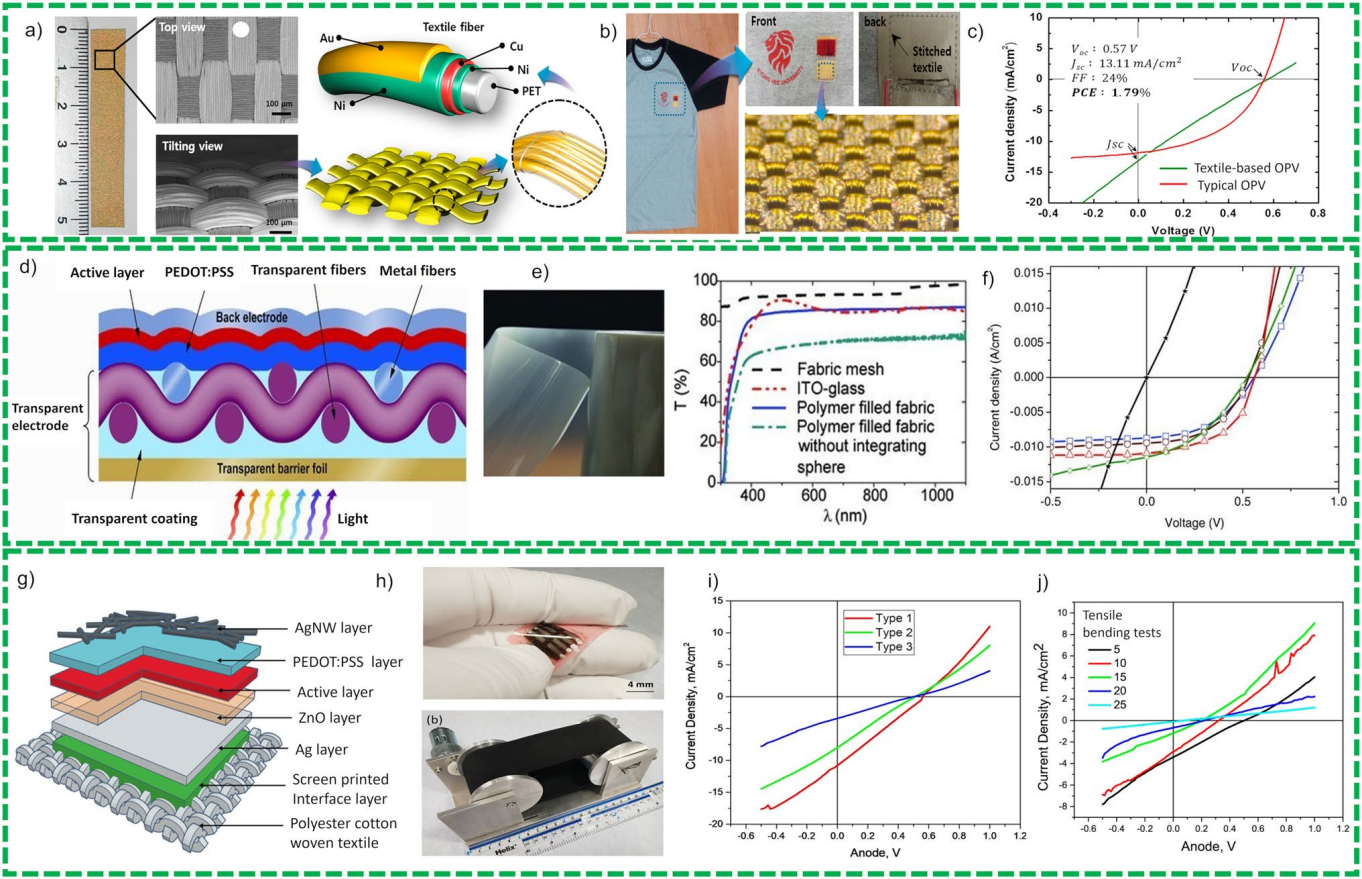
**a** Detail configurations of the large-area textile electrode. **b** Schematic images for the detail structure of the textile electrode Stitch able OPV with a textile electrode. **c** *J–V* curves of the textile-based OPV (green line and the typical OPV) (red line under 1 Sun illumination). Reprinted with permission from Ref. [49]. **d** Schematic side view of an organic solar cell including woven, transparent fabric electrode, active layers (PEDOT:PSS and P3HT/PCBM), and the aluminum back electrode. **e** Optical microscopy image of a woven fabric electrode before hole filling. A transmittance curve of a glass/ITO substrate is included for comparison (dash dotted, red lin**e)**. **f** *J–V* curves of fabric organic solar cells with bladed PEDOT: PSS layers of different thicknesses. Reprinted with permission from Ref. [66]. **g** Isometric pictorial representation a step one functional layer deposition of textile solar cells fabrication by spray coating, the full schematic diagram of the spray-coated textile organic solar cells with each layer defined, **h** photograph image of the encapsulated spray-coated solar cells on textile, and Photograph image of the bending machine for testing the durability of the spray-coated solar cells on textiles, **i** *J-V* characteristics of OSCs fabricated on FTO (type1), bare glass substrate (type2) and fabric substrates (type3) using the spray coating method, **j** *J-V* curves of device type 3 after outer and inner bending cycling tests. Reprinted with permission from Ref. [67]. (Color figure online)

First, OSC was presented by Lee et al. [49] in 2014 as stitch able power source into textiles. The authors employed ITO deposited flexible film at the bottom electrode, ZnO as the ETL, spin coated P3HT: PCBM as the active layer and MoO_3_ as HTL and gold coated textile as top electrodes as shown in Fig. 6a. The authors have also demonstrated a fabrication of flexible woven OSC in curved shape along with detailed structure of the device of the textile electrode, as shown in Fig. 6b. The electrode was fabricated by plating different metals such as nickel, copper, and gold. The fabricated OSC device exhibited a PCE of 1.97% for textile-based solar cells and 2.97 with silver as top electrode as demonstrated in Fig. 6c. Although the authors presented a new idea for the fabrication of the device, efficiency of the device persists on the lower side and purely physical links among gold and textile substrate and HTL have been established.

Later, Kylberg et al. [66] and Steim et al. [68] employed polymers (conductive and woven polymer) along with metallic wires. The open spaces between these layers were filled with transparent polymer materials deposited by immersing the woven fabric in the liquid polymers and exploiting doctor blade technique. The layer of PEDOT:PSS was loaded using doctor blade and P3HT:PCBM via spin coating on top of PEDOT:PSS. The aluminum is used as the top electrode. Figure 6d exhibits the complete fabrication of the devices and different coatings of active materials. Figure 6e demonstrates the woven device in which metal fabric is weaved with fibers that can be weaved in 1D or 2D directions. Different combinations of the electrodes have been tested, and the devices with fabric mesh have shown more transparency which provides larger surface area and hence enhances PEC. The device has shown a direct relation between thickness of the film and PEC of the devices. The device with metal mesh fabric and fibers has shown excellent performance at low thickness layer of PEDOT:PSS owing to the high transmittance of electrode and PEC of 2.2%. The referred device has also exhibited excellent cyclic and bending stability after 100 bending cycles at a radius of 0.6 mm.

Lately, Li et al. [67] demonstrated a textile-based OSC by printing and spray coating techniques in which they have exploited polyester and cotton weaved fabric in 65/35 ratio as flexible substrate. Here, they loaded the first three layers with a maximum coating thickness of 250 µm in search of reduced roughness on the flexible fiber-based substrate. Later, they have loaded with Ag electrode, ZnO, active layer, PEDOT:PSS exploiting spray coating as shown in Fig. 6g and h. Devices were encapsulated for protecting them from air and moisture which reduces cyclic life of the devices to 2 days, as compared to 30 days with protected encapsulation with no degradation of efficiency, and 60 days with 25% reduction in efficiency. Three different types of device combinations were investigated, and results are compared as shown in Fig. 6i. The device performance was demonstrated after multiple cycles in different bending radius. The device has exhibited excellent stability for initial cycles, and then, performance starts to deteriorate which can be ascribed to deterioration of the active layers. But still more investigation needed to enhance the stability of the device which can sustain bending and compressive stresses as demonstrated in Fig. 6j. One possible approach could be by locating the active layer at the neutral axis where bending stress can be reduced to zero and device stability can be increased. Therefore, more investigation is needed on this front to have a stable device.

##### Flexible Perovskite Solar Cells

**Fig. 7:**
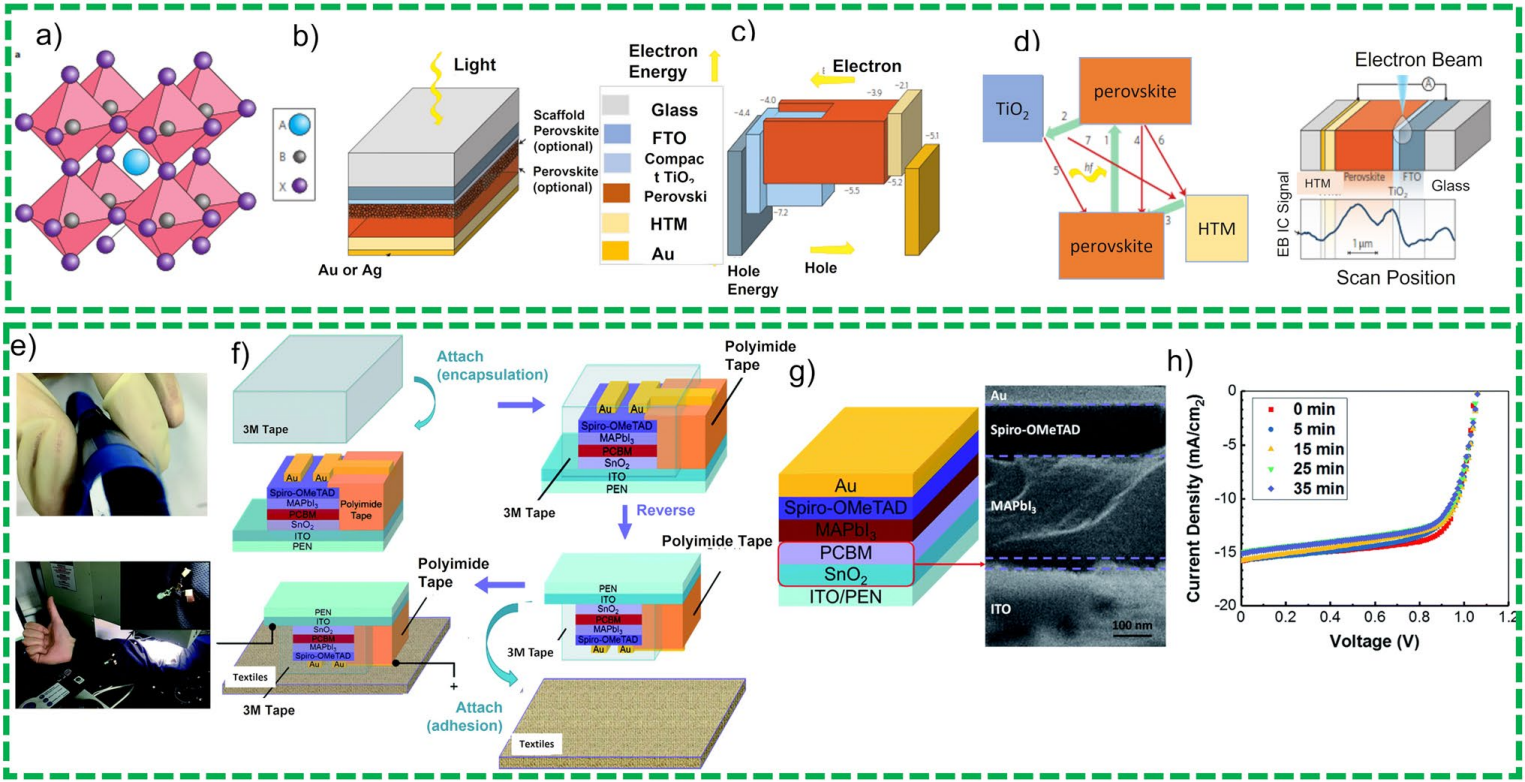
**a** Cubic crystalline structure of perovskite solar cell, here the bulky cation A is typically the methylammonium ion (CH_3_NH_3_), the minor cation B is Pb, and the anion X is a halogen ion (typically I, but both Cl and Br), **b** typical structure of perovskite solar cell, **c** vacuum energy level for constituents’ active materials, **d** demonstration of electron transfer process in the perovskite solar cell and pictorial demonstration of EBIC process. Reprinted with permission from the Ref. [70]. **e** Photograph of textile substrate-based perovskite solar cell, **f** graphic interpretation of development process of perovskite solar cells through functionalized encapsulation, **g** device structure and cross-sectional SEM view of the fabricated device, **h** *J–V* graph of the textile substrate-based perovskite solar cell with respect to immersing time in water [71]

Perovskite materials are a category of ingredients that have an empirical formula AB*X*_3_, where A, B are positive ions and *X* represent a negative ion of different charges and sizes as shown in Fig. 7a [69, 70]. Figure 7b demonstrates the typical structure of the perovskite solar cell which has demonstrated a PCE of 15%. Figure 7c exhibits the vacuum energy levels of a typical material used for Perovskite solar cells, e.g., (CH_3_NH_3_PbI_3_ mix halides and porous titania scaffold). Figure 7d demonstrates the key process involved in the perovskite solar cell starting from excitation of electron 1, 2 charge transfer to TiO_2_, 3-hole transfer to HTM, 4 recombination of the excited electrons, 5, 6 back transfers of electron at TiO_2_ and HTM interface, 7 charge transfer between TiO_2_ and HTM. Electron beam-induced current (EBIC) technique has also been exploited recently for the generation of the excited carriers. These perovskite materials have suitable attributes to be used as active materials for the solar cell such as maximum light absorption, excellent charge transfer, wide excitement diffusion length, simple tunable bandgap, and low excitation binding energy. These devices exhibit merit over the traditional solar cells for the simple fabrication processes and cost-effectiveness.

Lam et al. [71] presented a study in which they have integrated a washable and flexible perovskite energy harvesting system on a textile substrate as shown in Fig. 7e. First, tin oxide was electrodeposited on the flexible ITO-loaded polymer substrate as ETL and the active layer spun coated on tin oxide to reduce the roughness of SnO_2_ for enhanced electron transport as shown in Fig. 7f. The developed device was encapsulated by a 3M™ acrylic elastomer and adhered to the textile substrates as shown in Fig. 7g. The device has been immersed in the water, and *J–V* curves have been obtained as a function of time of immersion in the water. The device has shown a stable behavior during the immersion in water and exhibit the high barrier value in their solutions as shown in Fig. 7h. Even though, this work exhibited the fabrication of a textile-based perovskite solar cell with the good stability properties for the delivered applications, it is still demonstrating low flexibility as this device was directly fabricated onto the textile substrate. Therefore, the manufacturing of the integrated device has compromised flexibility properties such as folding and residual constraints for the attaining of complete device flexibility.

##### Textile-Based Solar Cells Comparison

From the last couple of decades, scientists have successfully demonstrated the fabrication and manufacturing of FDSSCs and counter electrodes which are significant for the development of integrated devices. Though, as the DSSCs are still exploiting glass-based photoanodes in the reported studies, the fabricated devices are not fully flexible, but it is considered first step for the manufacturing of the textile-based photovoltaics. Textile-based OSCs are flexible and suitable for wearable applications, but still they demonstrated inadequate performances in terms of PCE and stability. More sophisticated fabrication techniques are still required to fabricate solar cells which can harvest energy and can be integrated into textiles.

### Energy Storage Devices

#### Fiber-Shaped Supercapacitors

Since the start of the 21st century, it is impossible to imagine the activity of human society without flexible/wearable electronics such as laptops, activity trackers, or smart watches which have huge impact on improved economic productivity, human knowledge development and transfer which offer a convenience to human beings. Indeed, the scarcity of the energy arising from the fossil-fuels and environmental concerns increasingly force the scientists to explore new sustainable sources of the energy. Electrochemical energy storage devices in this aspect perform a very substantial role. Amid reported [72] electrochemical energy storage sources, supercapacitors, and batteries are considered as the most significant contenders on the Ragone checkerboard and have been a hot topic of research in both academia and in industry. In the recent past, flexible, and wearable electronics have attracted significant attention of the researchers and scholars due to its continuous emergence in diverse applications, such as biomedical, mobility, sports, consumer electronic devices, and clean energy. The operability of these devices has boosted the necessity for more efficient energy storage devices and their commercialization leading to new challenges [72]. Modern flexible electronic devices designs have revealed a new visualization of potential flexible wearable electronic devices such as implantable sensors, artificial skins, and other [73]. To cope with the requirements of the above-mentioned devices, storage devices must establish excellent performance standards even under constant mechanical stresses and deformations including folding, bending, stretching, and twisting for long cycles. For example, an artificial skin may conceivably present a resolution of obtaining real-time data by exploiting body sensors that can assist in regulating biological signals and health related information of the human while assisting human beings in varied conditions and activities. The fundamental challenge is to design and fabricate a FSEDs exploiting fiber-based electrode materials which demonstrate high specific energy and power and show exceptional cyclic stability using compatible electrolyte and separator. Other key parameters are low-cost fabrication and safety of the device which also attract significant importance especially when it’s implantable and fiber-shaped devices. Thus, the increasing applications of these flexible electronics in different fields will inflate the need for FBEDs for modern wearable/implantable devices. From the past couple of decades, electrochemical supercapacitors (ESCs) are attracting significant importance equally in industry as well academia owing to their good energy density (as compared to traditional capacitors) and excellent power density, long cyclic life, and fast charge/discharge rates, which is normally in seconds [74, 75].

The conventional ESCs possess inept structures (i.e., a separator sandwiched between two active materials (electrodes) wrapped in aqueous electrolyte), which poses key problems for their applications in realization and commercialization of wearable electronic devices. Major problem with traditional aqueous electrolyte-based SCs is its safety related issues where toxic electrolytes are used during fabrication which require high-quality encapsulation and technological advancement to avoid the leakage of these electrolytes. This encapsulation ultimately increases the weight of these devices and shows some shape constraints to be integrated into wearable electronics and other functional electronic systems. To address these issues and limitations in integration, flexible fiber-based SCs have appeared as innovative device for energy storage devices and engrossed extensive amounts of research in recent years [76, 77]. The FSCs are comprised of two flexible electrodes, gel–electrolyte (solid state or gel-like), a separator and an encapsulation material analogous to traditional SCs. The FSCs offer some advantages relative to traditional SCs, such as the exploitation of gel–polymer electrolyte and fiber-shaped electrodes that can be fabricated in smart designs, lightweight, thin shapes and miniaturized size thus enhancing their potential scope of applications in flexible and wearable electronics.

Research on flexible FSCs originated from the investigation on carbon and conducting polymer-based materials in early 90’s. During the first decades of 21st century, some breakthroughs have been observed in this field [78]. In these publications, SCs are typically fabricated by sandwiching the polymer-based electrolytes between two flexible electrodes in parallel configuration in which the active material is mainly deposited on stainless steel grids. Such approach to fabricate and assemble SCs devices poses two different problems: (1) limited control on the thickness of the device due to the thick layer of solid-state electrolyte and metal-based flexible current collectors; and (2) in this configuration normally electrodes are pressed against each other and become in contact with electrolyte. Therefore, only part of the electrode closed to electrolyte at electrode/electrolyte interface will efficiently be utilized. However, over the past decade the fabrication techniques have been improved to address these issues and remarkable progress have been reported in this regard [79].

##### Principles and Understanding of Charge Storage Mechanism

**Fig. 8:**
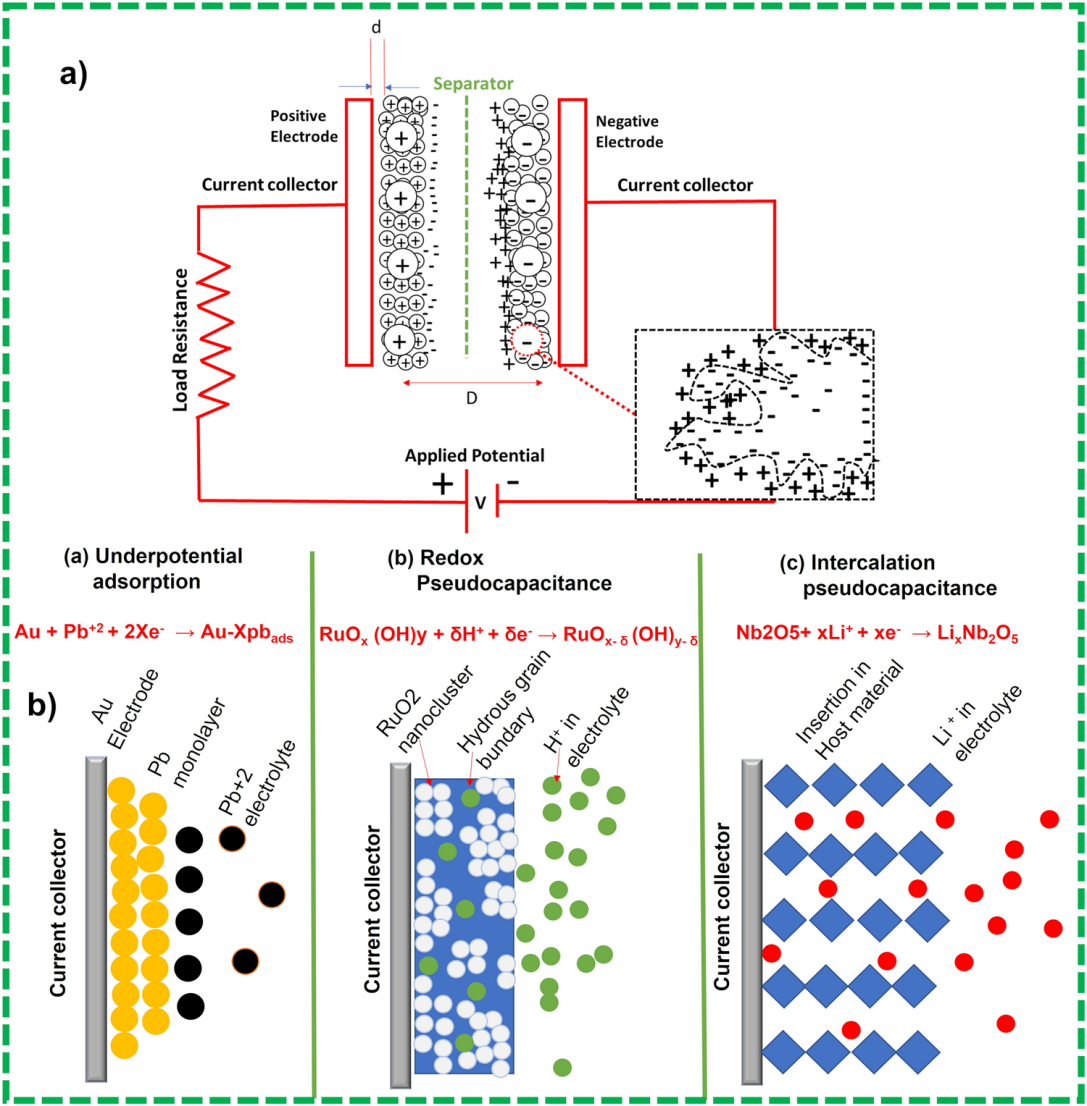
**a** Graphical demonstration of EDLCs charge storage mechanism and **b** various types of redox mechanism in PCs: (**a**) underpotential adsorption, (**b**) redox PCs, (**c**) intercalation PCs. Reprinted with permission from Ref. [76]

Numerous studies have briefly described the charge storage mechanism of SCs and its fundamental electrochemistry [80]. SCs are mainly classified into two types based on their charge storage mechanism [81]; (1) electrochemical double layer supercapacitors (EDLCs), and (2) pseudocapacitors. EDLC is a surface phenomenon and charge accumulation are done on the electrode/electrolyte interface. It is a physical process without any faradaic/redox reaction as shown in Fig. 6. The specific performance of the ELDC devices is governed by the surface properties of the active material such as specific surface area and pore size distribution [82]. It is recommended that pore size of porous material for ELDCs must be double the size of the electrolyte molecules/ions to grant full penetration to the pore walls. If there is no compatibility between electrolyte ions and pore dimensions of the active material, the solvated ions will not pass through the pores, but some research reports invalidated this postulation and calculated the highest ever specific capacitance values, even for the smaller pore sizes than electrolyte ions [83]. This shows that no clear correlation exists between specific surface area and capacitance, allowing to infer that large surface and average pore size have no significant impact on specific capacitance. However, according to some reports [84], in carbon-based material, capacitance increases ~ 1.5 fold when pore sizes < 1 nm are employed. This means that the carbon nanostructure (instead of specific surface area) and pore size play a significant role in enhancing gravimetric capacitance as shown in Fig. 6a. In the recent era, due to numerous in-situ spectroscopic studies and advanced computational techniques close investigation of developments and source of charge storage mechanisms in carbon materials have been reported [85]. Conway reported [76, 86] that different faradaic reactions are the outcome of capacitive electrochemical attributes as depicted in Fig. 8, such as: (1) underpotential deposition, (2) redox reactions normally observed in transitional metal oxides, e.g., RuO_2_·*n*H_2_O: and (3) intercalation and deintercalation pseudocapacitive normally observed in V_2_O_5_, TiO_2_. A brief account on pseudocapacitive behavior can be found in the latest reports [87]. In the pseudocapacitive behavior, charge accumulation is normally done via surface or near surface charge transfer responses. An intercalation/deintercalation charge storage mechanism was reported in which electrolyte molecules penetrate inside the structure of the redox active material or tunnel is complemented by faradaic charge transfer without compromising default structure of the crystal. In some of the recent reported studies, the term “pseudocapacitive” is confused with materials whose cyclic voltammetry and charge/discharge curves are like the attribute of battery materials such as (Ni (OH)_2_ and Co_*x*_O_*x*_ in KOH electrolyte). Conversely, the designated pseudocapacitive materials are those which demonstrate electrochemical behavior like carbon-based materials such as MnO_2_ and RuO_2_ [88]. Therefore, it is recommended that the term “pseudocapacitors” must be used for material which shows behavior like RuO_2_ in liquid electrolyte.

The charge stored in pseudocapacitive materials is governed by two different phenomena namely, (1) capacitive controlled charge storage; (2) diffusion-controlled charge storage. The total charge stored in pseudocapacitive materials is the sum of both capacitive controls and diffusion control charge storage [82]. Normally charge storage contribution is determined by using the following relationship:

2 $$i = a\nu^{b}$$

where $i$ is the current on the scan rate *ν*, *a* and *b* are adjustable parameters? The parameter *b* is estimated from the slope of the curve by taking the log ($i$) and log(*ν*). If the *b* = 0.5, the corresponding charge storage is considered diffusion-controlled faradaic intercalation reaction and represents the traditional battery like behavior and current is proportional to square root of the scan rate considering the following expression:

3 $$i = n{\text{FAC}}^{*} D^{1/2} \nu^{1/2} \left( {\alpha nF/RT} \right)^{1/2} \pi^{1/2} \chi^{bt}$$

where C* is concentration of the active material of the electrode, $\alpha$ is the coefficient of transfer, *D* is coefficient of diffusion, *n* is the number of electrons participating in the electrode reaction, *A* is the area of the electrode material, *F* denotes the Faraday’s constant, *T* represents temperature, *R* designated to molar gas constant and *bt* is normalized current value. On the other hand, in a capacitive controlled process, current is proportional to scan rate, i.e., *b* = 1. The total charge stored in the device is the sum of the capacitive controlled charge stored *k*_1_*ν* and diffusion-controlled charge storage *k*_2_*ν*^1/2^_._ Therefore, Eq. 1 can be modified as follows:

4 $$i = k_{1} \nu + k_{2} \nu^{1/2}$$

The expression above shows that total current accumulated at fixed potential is the sum of capacitive contribution (*k*_1_*ν*) and diffusion-controlled contributions (*k*_2_*ν*^1/2^). Here, *k*_1_ and *k*_2_ are constants which are determined by drawing a graph between $i/\nu^{1/2}$ and $\nu^{1/2}$ and computing the angle and *y*-axis intercept point of the straight line correspondingly.

##### Performance Evaluation of Supercapacitors

There is ongoing debate in the literature for the evaluation of supercapacitors because standard practices are yet to be formulated about different configurations of the supercapacitors, e.g., three electrode studies and two electrode configurations in symmetric and asymmetric, active material film thickness, constant current charge and discharge, cyclic voltammetry, sweep rate and current density among others, which yields different values. The specific capacitance of the cell can be calculated using Eq. (5):

5 $$C_{{{\text{cell}}}} = \frac{Q}{2V} = \frac{1}{2V\nu }\mathop \int \limits_{v - }^{v + } i\left( V \right)\,{\text{d}}V$$

where *C*_cell_ is the capacitance of the cell, $i$ represents current, ν denotes scan rate, and *V* (*V* = *V*^+^ − *V*^−^) is the potential window of the cell. The gravimetric capacitance is computed by using Eq. (6):

6 $$C_{{{\text{sp}}}} = \frac{{C_{i} }}{m} = \frac{{2C_{{{\text{cell}}}} }}{m} = \frac{{4C_{{{\text{cell}}}} }}{m}$$

where *m* represents the mass of the electrodes. The energy and power densities can be computed using Eqs. (7–10):

7 $$E = \frac{1}{2}C_{{{\text{cell}}}} V^{2}$$

8 $$E_{i} = \frac{1}{2}\frac{{ C_{{{\text{cell}}}} }}{m} V^{2}$$

9 $$P = E/t_{{{\text{discharge}}}}$$

10 $$P_{i} = \frac{P}{m}$$

where *C*_cell_ is the capacitance computed by using Eq. (5), *E* and $E_{i}$ are energy and specific energy density, respectively, *m* is the mass of the material used as active material, *R*_ESR_ is the equivalent series resistance, *t*_discharge_ is the time of discharge of the device, *P* and *P*$i$ are the power and specific power, respectively.

##### Innovative Approaches in Cell Configuration

**Fig. 9:**
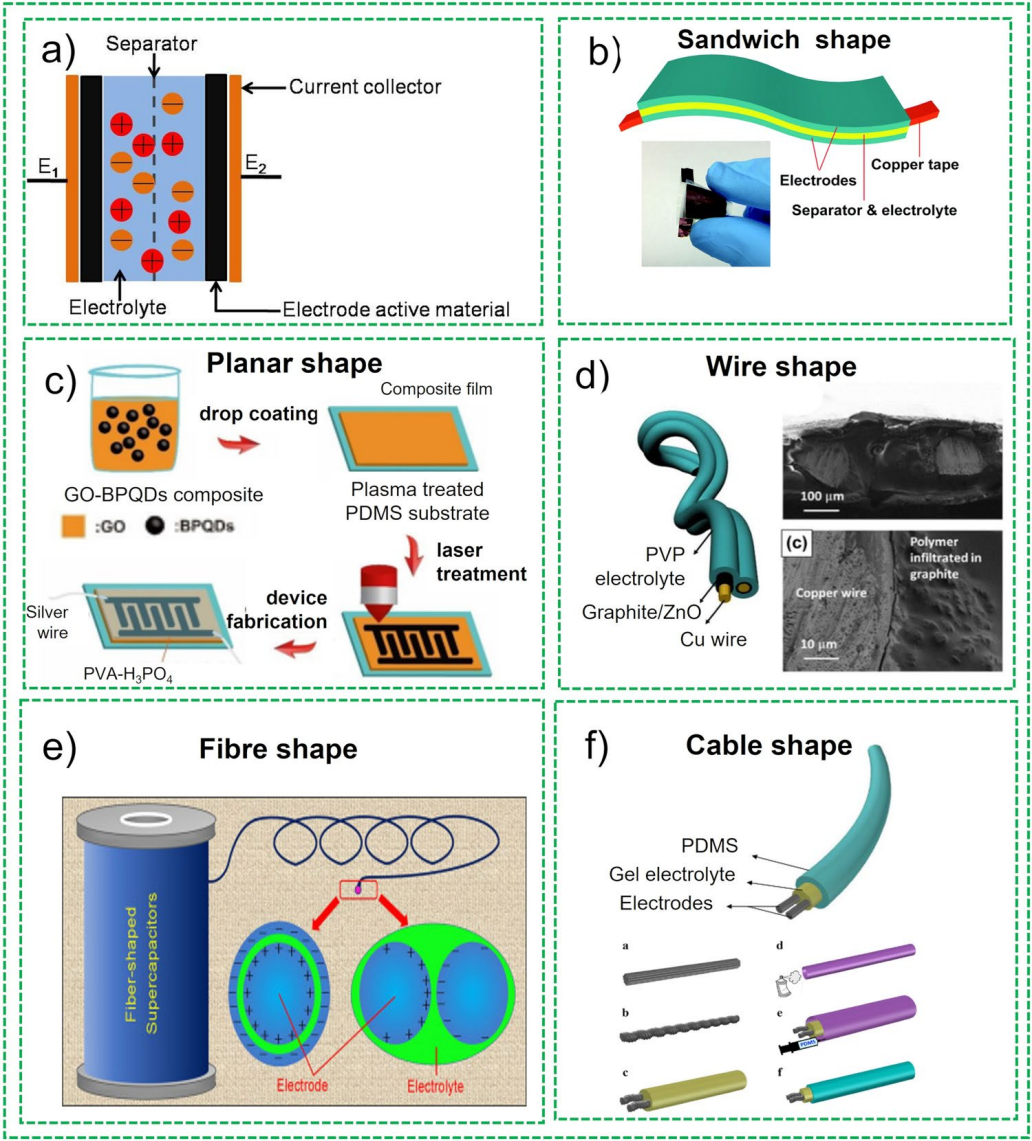
**a** Typical structure of the supercapacitors, **b** Sandwich shape supercapacitors. Reprinted with permission from Ref. [89]. **c** Planar shape supercapacitors. Reprinted with permission from Ref. [90]. **d** Wire shape supercapacitors. Reprinted with permission from Ref. [77]. **e** Fiber shape supercapacitors. Reprinted with permission from Ref. [91]. **f** Cable shape supercapacitors Reprinted with permission from Ref. [89]

Although different cell design configurations have been proposed in the literature, cutting edge research aiming breakthroughs on efficiency of cell design configurations of FBSCs has been preferred to exclusively investigating active materials for SCs. Figure 9a demonstrates the different innovative cell design approaches exploited for the fabrication of the FBSCs proposed in the last couple of decades to use in flexible and bendable wearable electronics. These are discussed in detail in the following section.

*Sandwich-Shape Structure* Sandwich type configuration for the fabrication of the FBSCs is the most commonly exploited configuration because of its easy fabrication of electrodes and assembly of devices as shown in Fig. 9b [92]. In this design, two flexible electrodes are placed parallel to each other with gel-polymer electrodes sandwiched between them wrapped in a flexible packaging. Mechanical flexibility is the most critical property for flexible fiber-shaped supercapacitors because they are exposed to extensive mechanical stresses in real-life applications. Therefore, mechanical properties are evaluated by performing electrochemical characterization such as bending stability, folding and twisted conditions. Owing to easy fabrication and assembly, this type of configuration is the most studied design in recent studies [77, 81]. Nevertheless, due to the absence of separator in this configuration, complete protection from short circuiting is not guaranteed.

*Planar or On-Chip Structure* Planar configuration of the SCs device fabrication is an emerging design of the device and has the advantage of making thinner and more flexible devices than current design of fabrication techniques of devices, due to their distinctive structural pattern, as shown in Fig. 9c [90, 93]. The device comprises three important constituents integrated into a 2D configuration in the same plan, which include active material for electrode, current collector, and electrolyte. The incumbent configuration offers enormous channels for electrolyte’s ions and accelerates charge transfer in 2D direction. Additionally, this configuration demonstrates configuration stability and does not affect the electrolyte ions movement even if the devices are subjected to different bending conditions such as rolled and folded conditions.

*Fiber-Shaped Structure* In the recent past, owing to growing demands for wearable and portable electronics, fiber-based SCs have attracted significant attention from investigators and researchers. Thanks to the structural integrity of fibers, fiber-based SCs offer unique advantages over sandwich and planar-shape SCs in terms of structural variety, flexibility, and light weight. In addition to above-mentioned attributes, conventional configurations (sandwich and planar) are unable to match specific capacitance and power volume. Fiber-based SCs offer numerous structures, and focused efforts have been made in developing and designing novel configurations and construction, as shown in Fig. 9d [91]. One simple example of this configuration is assembling two parallel fiber-based electrodes dipped in gel-polymer electrolyte in parallel, on a flexible substrate as depicted in Fig. 5 [1, 76, 81]. Fiber-based SCs also offer the benefit of easy integration of single fiber SC in parallel and series which help in enhancing current density and voltage to increase power and energy densities needed to meet the requirement for practical applications in portable and flexible electronics. Carbon-based active materials such as carbon fibers, carbon nanotubes, mesoporous carbons and graphene-based materials are exploited to fabricate different fibers and yarn shapes. These shapes can also be hybridized with other active materials to synthesize a composite material for faradaic pseudocapacitance, such as transition metal oxides and conducting polymers.

*Wire-Shape Structure* The increased demand for flexible and wearable electronic devices wire-type SCs needed that can easily be woven into textile or wearable electronics. The conventional configuration of device fabrication described above offers many advantages, but they are not classified as fiber-based SCs in true sense, because they are exploited as support to devices. True fiber-based SCs are fabricated as an 1D-wire structure in which one fiber-based electrode is coiled around the other electrode with separator or gel-polymer electrolyte filled in between the electrodes [77]. For example, wire-type SCs is fabricated using two carbon fiber-based yarns in which one yarn thread is coated with cellulose acetate as separators and other carbon-based yarn thread is coiled around the first yarn soaked with simulated sweat solution and gel-polymer as electrolyte. This configuration is also classified as coil-type SCs as shown in Fig. 9d. The electrochemical characterization of these configurations can be performed under different mechanical stresses to evaluate the performance of the device, such as winding, stretching, bending, and folding conditions. However, these stresses might cause damage to coated gel-polymer electrolyte and two wire electrodes may get separated from each other in these conditions and deteriorate the performance of the device. Therefore, best configuration structure is not finalized yet that can sustain these mechanical stresses for mechanical stability.

*Cable-Shape Structure* Wire-shaped SCs have some disadvantages such as short-circuiting and electrolyte seepage challenges because fiber-based electrodes in this configuration are in direct contact with each other, which makes them incompatible for real-life applications. To counter these problems, researchers have offered a novel technique for the fabrication of SCs devices called cable-type supercapacitors as shown in Fig. 9e [89]. The basic principle of this configuration is exactly like wire shape SCs device but in this case an additional wire is introduced in the structure to act as spacer, which is evenly coiled around one of the electrodes with specific field as demonstrated in Fig. 9f. This additional wire is also required in conventional flat SCs to avoid the short circuiting of the electrodes that are in direct contact. The first device of this configuration was fabricated in 2012, in which commercial pen coated electrodes are used as active material along with gel-electrolyte and coiling spacer wires [94]. This spacer wire not only plays a role of prevention of short-circuiting during folding and bending but also offers efficient transfer of electrolyte ions and support large-scale fabrication of FSCs.

In summary, there are three main cell configurations sandwich, planar and fiber-based supercapacitors. Fiber-shaped SCs are further classified as plan, sandwich, and cable type. Wire based or cable type SCs are most suitable for textile and wearable electronics. All three configurations are important with reference to application of these devices. Thus, the assembly of FBSCs must conform to prerequisites to be exploited for wearables including safety, mechanical flexibility, and excellent charge accumulation to enable higher energy density devices that are able to comply with application requirements. Here, flexibility of the devices is frequently cited as a mandatory feature for portable and wearable electronic devices.

##### Components of Supercapacitors

The SCs devices normally comprise the following components as demonstrated in Fig. 9a:

1. Working electrodes or active material
2. Electrolyte
3. Separator
4. Current collectors

A SCs is composed of two working electrodes, a separator, gel-polymer electrolyte and two current collectors. However, active materials, electrolyte and current collectors are the most important elements of the device owing to their significant role in the device operation and performance especially for wearable and portable electronics. High cyclic life and excellent mechanical stability are core attributes of highly porous active materials and flexible current collectors. We will discuss current collectors in detail here in this review study and its different form in FSSCs.

##### Current Collectors for Flexible Supercapacitors

Before exploiting flexible substrate and current collector for fiber-shaped SCs, freestanding active materials films are used, but these films are relatively fragile and cannot sustain prolonged mechanical stresses. To counter these problems, research has proposed the use of flexible current collectors for SCs development and fabrication. Flexible substrates are considered the most critical component in fabrication and development of FBSCs which shows high electrical conductivity for fast charge transportation and excellent mechanical stability to sustain high mechanical stresses when exposed to real-life applications. Considering recent applications and technological developments, the following flexible substrates have been developed and exploited for flexible electrodes which are mainly classified into three broad categories:

1. Fiber-shaped substrates
2. Paper-shaped substrates
3. 3D porous substrates

**Fig. 10:**
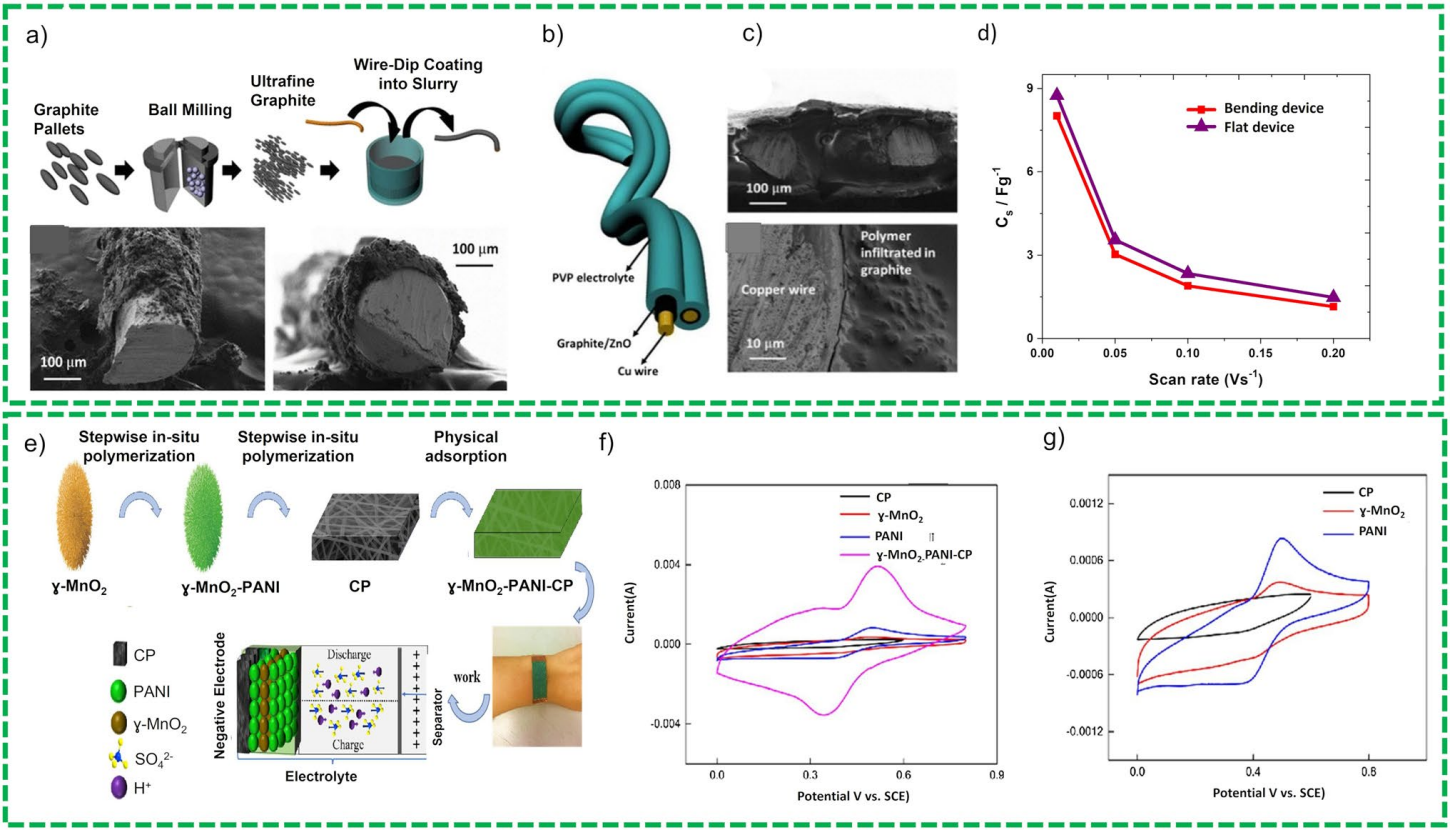
**a** Schematic representation of fabrication of the wire-based electrode, **b** pictorial representation of two parallel electrode configuration, **c** SEM image shows composite (graphite/ZnO) electrodes and gel-polymer electrolyte sandwiched between them, **d** bending stability tests. Reprinted with permission from Ref. [77]. **e** Graphical flow diagram for MnO_2_/PANI-based electrode fabrications, **f** CF ration of carbon paper and pore size distribution, **g** bending stability test of the electrode. Reprinted with permission from Ref. [100]

*Flexible Wires/Flexible Metal Matric/Metallic Fabrics* Metal wires or metallic fabrics or meshes are extensively used as flexible current collector for SCs due to their excellent mechanical strength and high electrical conductivity [95]. For this purpose, various metal-based substrates have been explored including stainless steel (SS) [96], copper (Cu) wire [97], nickel (Ni) [95], and titanium (Ti) wire [98], which provide easy fabrication of electrode, high mechanical strength, and excellent conductivity. In addition to flexibility, the active materials deposited on these substrates during electrodes fabrication results in enhanced specific capacitance, energy, and power density, respectively. On the other hand, powder-based active materials need binder for better adherence to the substrate resulting in low value of capacitance and energy density. Rafique et al. [77] deposited porous nanostructures of ZnO/graphite composite on copper wires as shown in Fig. 10a and b. Fiber-shaped SCs comprise these ZnO/graphite-based electrodes that were fabricated using polyvinyl pyrrolidone in 1 M NaCl as gel-polymer electrolyte and offered a specific capacitance of 27 F g^−1^ as shown in Fig. 10c and d. The device demonstrated an excellent cyclic and bending stability and exhibited insignificant deterioration in electrochemical performance under different bending stresses. The device shows 100% retention of capacitance after 500 cycles. Similarly, Cu (OH)_2_ deposited directly on Cu foil substrate offer large numbers of active sites for electrolyte ions and redox reactions which are easily accessible for the electrolyte’s ions [99]. A flexible and bendable SCs is fabricated in asymmetric configuration in which Cu (OH)_2_ and AC (activated carbon) are used as anode and cathode, respectively. This device has demonstrated high energy density of 3.68 mWh cm^−3^ and power density of 5314 mW cm^−3^ which was used to lit 26 LEDs as an example of its applicability.

Regardless of these improvements, the conducting current collectors exploited for fabrication of FSSCs (SS, Al and CU foil) are generally bulkier and thicker than deposited active material. This increase in thickness of the films results in enhanced gravimetric capacitance which strongly depend on thickness of the active material and decrease in the thickness of the film will slightly increase the specific capacitance for the device. But the problem with the thick film of the active material is that it increases the path length between the conductive substrate and surface of the active material which consequently deteriorates the capacitance and rate capability. These problems can be encountered by exploiting 3D conductive structures as current collectors which enables high mass loadings. Avasthi et al. [101] grew vertically aligned CNT coated with titania on stainless steel mess using chemical vapor deposition and demonstrated excellent capacitance performance. The assembled FSSCs show a specific capacitance of 16.24 mF cm^−2^ at a specific current density of 1.67 mA cm^−2^. Zhou et al. [102] synthesized a core–shell composite structure comprised of layered titanate nanowires covered with Ni(OH)_2_ nanosheets on titanium mesh by simple ion-exchange method. FSSCs are fabricated using K_2_Ti_2_O_9_@Ni(OH)_2_/Ti mesh covered with a gel-polymer electrolyte PVA/KOH. The devices showed a capacitance of 5.8 mF cm^−2^ at 5 mV s^−1^ with capacitance retention rate of 93% after 2000 cycles.

It is noted that FSSCs exhibit excellent mechanical stability and reliability with negligible electrochemical performance deterioration after subjecting to mechanical stresses of more than 100 intentional bending cycles. Kambel et al. [103] recently grew a binder free FeCo_2_O_4_ thin film on a flexible stainless-steel mess and used this material for SCs applications. The nanostructures of various morphologies were grown by varying the temperature (80, 100, 120 and 1400 °C) using reflux condensation synthesis technique. The material synthesized at 120 °C exhibits a good electrochemical performance with 260 F g^−1^ specific capacitance at 1 mA cm^−2^ and 96.16% capacitance retention after 1000 cycles shows excellent cyclic stability.

*Paper-Based Substrate* Although metallic-based substrates exhibit excellent conductivity for SCs applications, they also offer low resistance to corrosion which limit their application in energy storage devices. In addition, the use of these substrates as current collectors enhances bulkiness of the whole SCs device. On the contrary, carbon-based substrate such as carbon paper (CP), carbon foam and carbon cloth, demonstrated excellent electrical conductivity, good corrosive resistance, good flexibility, and light weight. Thus, as compared to metal-based substrates, carbon-based substrates are considered excellent contenders for FBEDs applications. Here, in this section, latest developments in carbon-based flexible substrates for FBSCs are discussed.

CPs are composed of standard arrangements of microfibers of carbon in plan sheets with numerous nanoscale pores that offer larger specific surface area to accommodate the active material. The network of microfibers constitutes the CPs substrates that are well linked with other microfibers to establish a conducting network that permits suitable pore channels to be formed. These channels offer an effective network of ions/electrons transportation and efficient electrolyte access to electrochemical sites of the active material. Thanks to the high porosity, mechanical integrity and excellent conductivity, CPs are widely explored as current collectors for SCs, fuel cells and other applications.

Numerous types of CP have been applied in different devices, ranging from hygiene and sanitation purposes to decorating and packaging of the materials. Moreover, CP has also been extended to portable and flexible electronic devices including circuits, flexible displays, photoanodes and transistors. Recently, researchers have shown that CPs also provide support for depositing active material for fabrication of high-performance SCs. Ling et al. [100] prepared a carbon fiber paper substrate having low weight, high flexibility, and foldability. CP exhibited very low internal resistance and high porosity. A core–shell structure of ɣ-MnO_2_ wrapped PANI is fabricated and modified stepwise by in situ polymerization technique to load core–shell structure uniformly on CP substrate by the physical adoption method and results in a flexible electrode material for SCs known as ɣ-MnO_2_-PANI-Cp substrate as shown Fig. 10e and f. The electrode shows a very high value of specific capacitance of 625 F g^−1^ at current density at 1 A/g and very high energy density and power density of 114.2 Wh kg^−1^, 798.6 W kg^−1^ as shown in Fig. 8g. The electrode demonstrated an excellent stability and retained 81.3% of its initial capacitances after 5000 cycles.

Although performance of these CP based flexible electrodes remains low, the performance can be improved by making a composite with some pseudocapacitive material loaded on carbon-based SCs. Rahman et al. [104] enhanced the performance of the PANI based supercapacitors by sodium phytate-doped PANI nanofibers deposited on different type of CP for supercapacitors application. During analysis, it exhibited an excellent relationship between the material properties and nanostructures. The assembled device exhibits a remarkable characteristic, good charge transfer and specific capacitance of 1106.9 and 779 F g^−1^ at current density 0.5 and 10 A g^−1^, respectively, achieved at optimized conditions. The device has shown excellent cyclic stability of 96% capacitance retention after 1000 cycles at current density of 10 A g^−1^. Another paper-based device [105] was also assembled in asymmetric configuration by using Ni/MnO_2_ filter paper as cathode and Ni/AC as negative electrode in a PVA/Na_2_SO_4_ gel-polymer electrolyte. Strikingly, the assembled asymmetric device demonstrated a volumetric specific energy of 780 Wh cm^−3^. Additionally, the device has displayed exceptional flexibility under different mechanical stresses and bending conditions.

*Textile Substrates* Textile substrate-based flexible electronic devices such as wearable electronic devices, electronic textiles and intelligent textiles can be exploited for the development of future high-tech textiles for different applications such as workwear, health monitoring systems, sportswear, portable energy systems, and defense applications [106]. These integrated wearable devices need a continuous supply of energy to operate, and FSSCs have shown a great potential for these applications. Typically, textile substrates: polyester, acrylonitrile, and cotton because these substrates, can be recycled and are low-cost, hydrophilic, and of course, flexible. When considering practical applications of these substrates, they offer many advantages other than flexibility and stretchability over CP-based substrates such as porous structure for optimum loading of active materials and facilitating quick absorption of electroactive constituents due to their hydrophilic properties. This optimum loading of active material leads to higher specific capacitance, specific energy, and power, respectively [107].

**Fig. 11:**
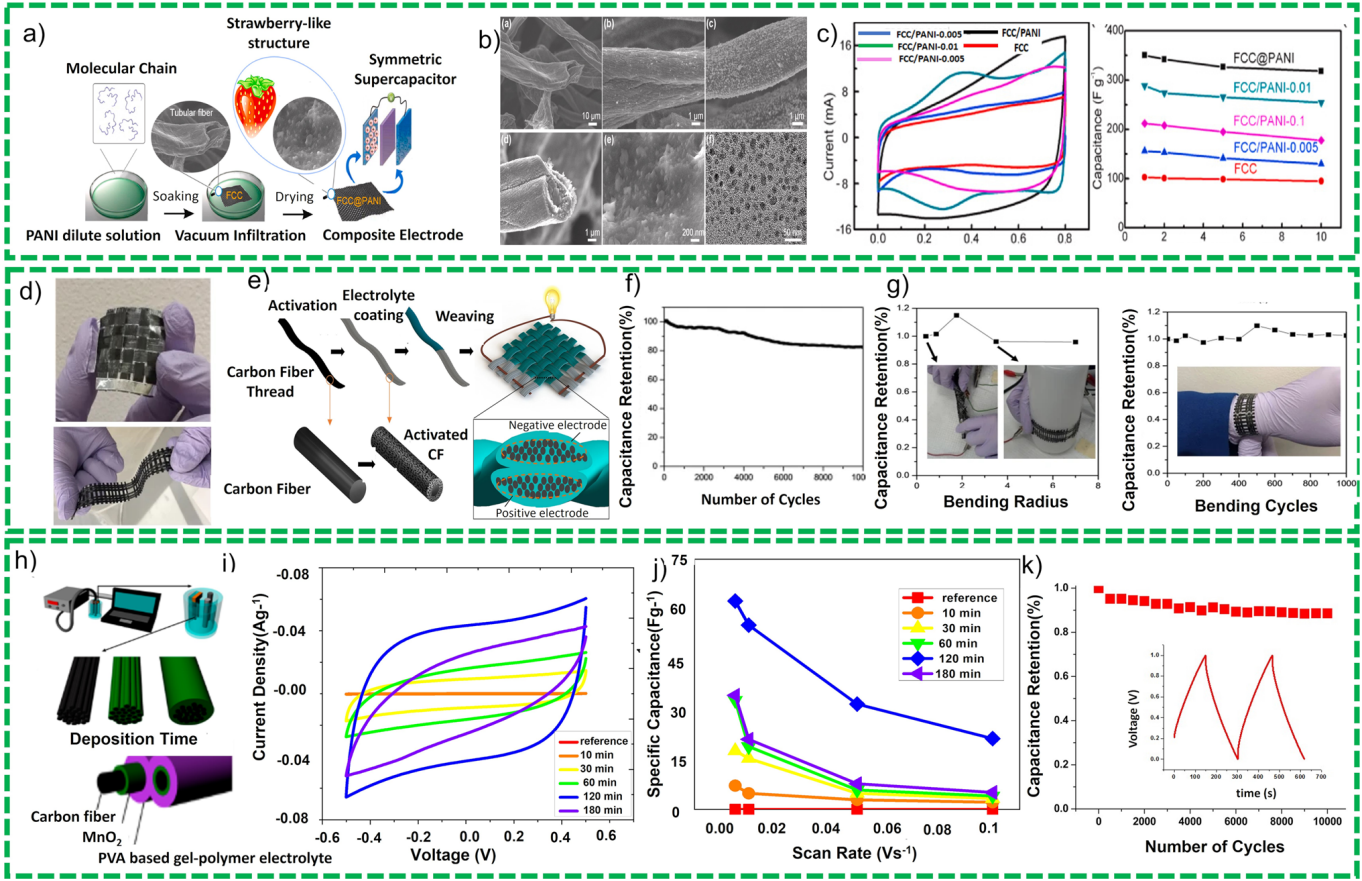
**a** Graphical representation of strawberry-alike FCC@PANI composite by vacuum penetration technique, **b** SEM images of FCC and FCC@PANI composite synthesized exploiting vacuum penetration technique, **c** electrochemical characterization of FCC, PANI@FCC composite cyclic voltammetry, and specific capacitance comparison at different scan rate, and cyclic stability. Reprinted with the permission from Ref. [108]. **d** Pictorial representation of carbon fabric-based SCs, **e** schematic representation of fabrication of activated carbon fabric-based supercapacitors, **f** cyclic stability of device, **g** bending stability of the devices. Reprinted with permission from Ref. [109]. **h** Schematic representation of electrochemical deposition of MnO_2_ characteristics with cyclic stability, **i** and **j** comparison of electrochemical characterization of electrodes at different time of deposition, **k** cyclic stability of the device. Reprinted with permission from Ref. [1]

*Cotton Cloth-Based Substrate* Hong et al. [108] prepared a PANI functionalized carbon substrate by vacuum infiltration method known as FCC@PANI nanocomposite. Here, functional carbonized cotton cloth was soaked in dilute PANI solution under vacuum condition to obtain a penetration of PANI inside the substrate as shown Fig. 11a. After evaporating the solvent, strawberry shape like FCC@PANI nanocomposite synthesized with PANI nanoparticles incorporated in the fiber surface. This technique of manufacturing nanocomposite avoids the formation of nanofibers of PANI and restricts their agglomeration even at higher loading of PANI, i.e., 28 wt%. The device assembled using these electrodes demonstrated high value of specific capacitance 351 F g^−1^ at current density of 1 A g^−1^ and has shown excellent cyclic stability with capacitance retention of over 90% after 10,000 cycles as shown in Fig. 11b and c.

*Carbon Fabrics* This type of textile-based substrates is easy to synthesize and weave, but it has some limitations of low electrical conductivity for preparation of textile-based electrodes. From this perspective, carbon fiber fabrics, which offer flexibility, porosity, and electrical conductivity, are better contenders for body materials in textile-based substrates as shown in Fig. 11d [109]. Figure 11e demonstrates the fabrication of activated carbon fabric-based supercapacitors. The device has exhibited an excellent electrochemical characterization of 18.6 and 17.8 F g^−1^ and has shown remarkable cyclic stability after 10,000 cycles as evident from Fig. 11f. The device has also demonstrated an excellent bending stability after bending at different radii and has shown 100% capacitance retention after 1000 cycles as depicted in Fig. 11g. Gu et al. [110] have also used carbon fiber-based cloth for supercapacitive applications. Carbon fiber-based cloths are first thermally activated by simple annealing at 450 °C to increase the specific surface area of the substrate and to achieve excellent electrochemical performances of the SCs. The capacitance of the devices after activation of carbon cloth is 1484 times higher than untreated carbon fiber clothes-based electrodes (1136.7 vs. 0.96 mF cm^−2^ at 2 mV s^−1^), chiefly due to incorporating the surface oxygen functional groups which results in significant improvement in surface area. The full cell device was also fabricated of carbon fiber clothes-based electrodes which demonstrated excellent electrochemical performance in terms of specific capacitance and energy density with capacitance values 554 mF cm^−2^ and 77 µWh cm^−2^, respectively. The device also exhibited a remarkable rate capability of 70 µWh cm^−2^ at 50 mA cm^−2^ and good cycling stability up till 50,000 cycles.

*Carbon Fibers* Fabrication of high-performance SCs based on carbon fibers poses many challenges that must be addressed before fabrication of the devices. The carbon fibers are inactive for SCs because of poor electrochemical performance, low surface area and poor porosity as compared to graphene and CNT. Rafique et al. [1] used carbon fibers and functionalized this carbon fiber with electrodeposited manganese dioxide and used them for SCs applications as shown in Fig. 11h. SCs device was assembled using MnO_2_/CF electrodes in symmetric configuration, and the device showed an excellent specific capacitance of 63 F g^−1^ as depicted in Fig. 11i and j. The device was also checked for cyclic and bending stability, and device has shown 90% capacitance retention after 10,000 cycles and 95% capacitance retention after different stress levels at different angles as shown Fig. 11k. Serrapede et al. [82] also fabricated a carbon fiber-based asymmetric device in which 2-step electrodeposited MnO_2_ is used as cathode and rGO/Fe_2_O_3_ as anode material. The devices have shown a wide range of potential window 1.4 V and high values of energy and power densities. The device exhibited good cyclic and bending stability with almost no loss of specific capacitance after 2500 cycles and 75% of capacitance retention after bending from 0° to 180°.

*Electrospun Carbon Nanofibers* Carbon nanofibers (CNF) are widely exploited for energy storage applications because of its inherent properties such as excellent electrical conductivity, structural stability, and large specific surface area. CNFs in energy storage devices are mainly used in batteries and SCs in multi-functional roles such as active electrode materials, as a substrate to support active material loading, and sometimes as conductive additive. Electrospun CNFs are low-cost, scale-able and offer the advantage of making hybrids with tunable nanostructures. Allado et al. [111] fabricate a binder-free electrode by depositing a binary metal oxide such as MnO_2_/Co_3_O_4_ on electrospun CNFs which helps to overcome the problem of low active site and resultant energy density due to binders. The SEM analysis discovered that both the materials are uniformly wrapped around the CNF to offer porous morphologies with high energy storage capacity. The three-electrode electrochemical analysis indicated that fabricated electrodes showed a capacitance of 728 F g^−1^ for MnO_2_/Co_3_O_4_/ECNF in 6 M KOH and 622 F g^−1^ for MnO_2_/ECNF at 5 mV s^−1^. Asymmetric assembled device exhibited high energy and power density of 64.5 Wh kg^−1^ and 1276 W kg^−1^, respectively, and cyclic stability tests revealed a capacitance retention of 72% approximately after 11,000 cycles.

**Fig. 12:**
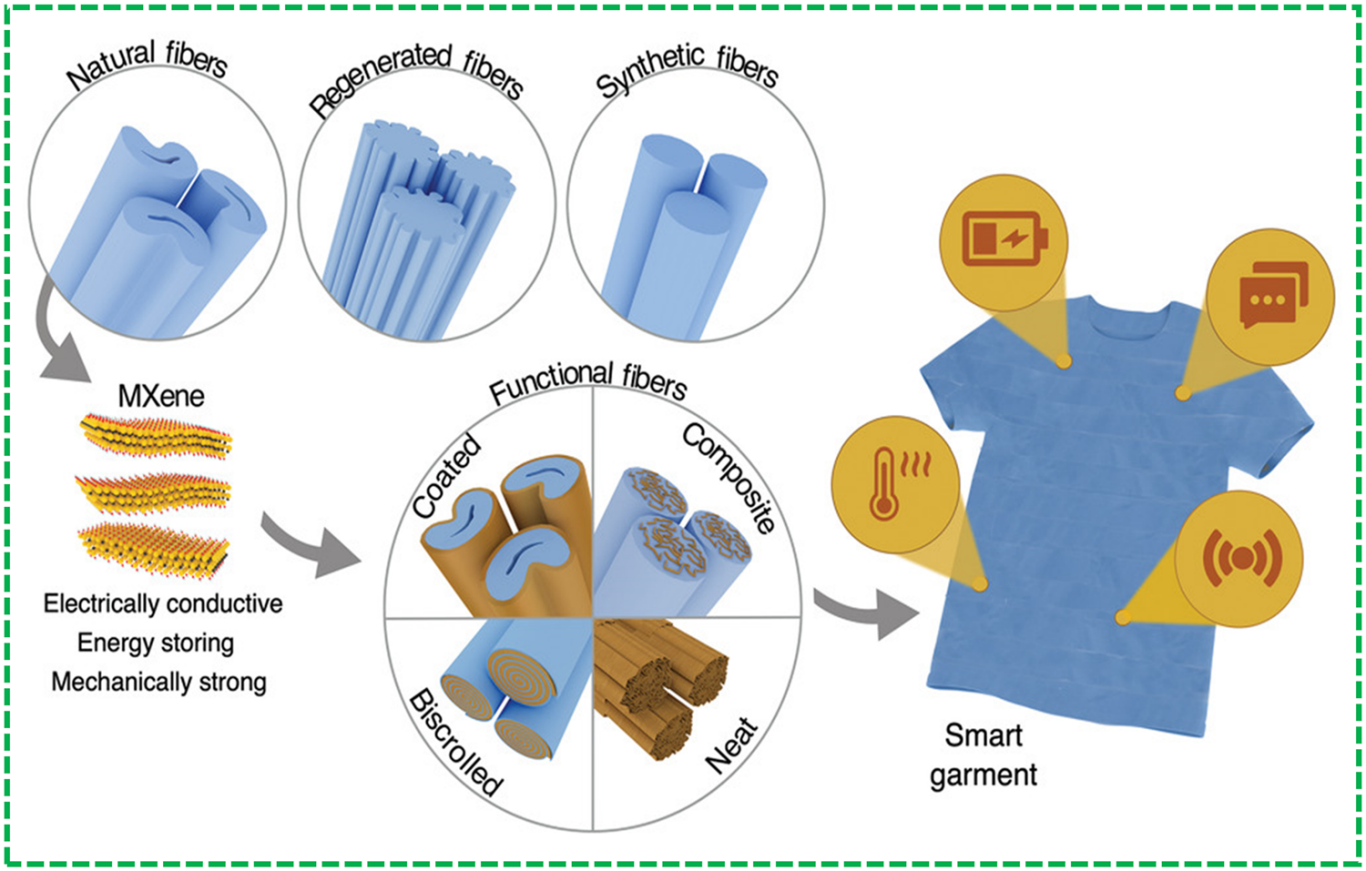
Pictorial demonstration of the growth of functional fibers by incorporating MXene into fibers from natural, regenerated, and synthetic sources that facilitate smart garments have potential of accumulating charge, harvesting energy, heating, sensing, and communicating with nearby electronics. Reprinted with permission from Ref. [116]

*2D MXene Fibers* The recent advances in internet of things (IoT), portable and flexible wearable electronics assert the new prerequisites for substrates such as fibers, together with high conductivity, store energy, and sense physiological signals and movements [112]. In the recent past, the researcher coined a new term in this category and referred it as “functional fibers” [113] which are produced by compositing highly conductive, high potential for energy storing and mechanically strong materials. MXene (represented as *M*_*n*+1_*X*_*n*_*T*_*x*_, where *M* denote early transition metal, *X* represents carbon and/or nitrogen, *n* = 1–4, and *T*_*x*_ = F, O, and/or OH) [114] is considered a new class of emerging 2D materials, e.g., 2D carbides, nitrides and carbonitrides, into fibers as shown Fig. 12. MXene fiber has attracted considerable attention owing to remarkable electrical and excellent electrochemical properties as well as ease of processability. Since its discovery a decade ago [115], 30 different compositions of MXene have been synthesized and reported with Ti_3_C_3_T_*x*_ the most studied composition possess exceptional electrical conductivity 20,000 S cm^−1^ and exhibited specific capacitance of ∼ 1500 F cm^−3^ in protic electrolyte.

**Fig. 13:**
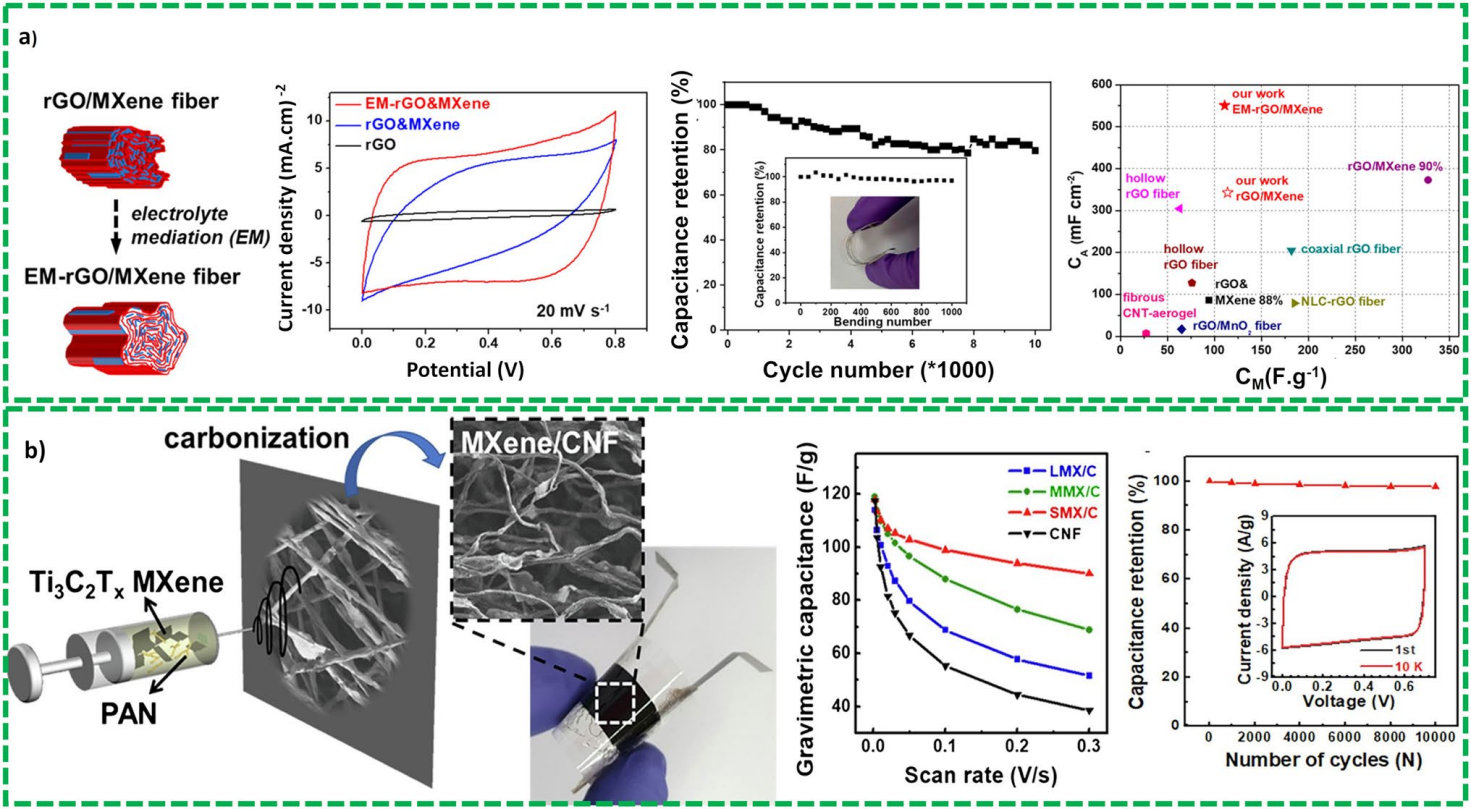
**a** Synthesis of hybrid MXene-based fibers using wet spinning, comparison of electrochemical performance, cyclic stability comparison and Ragone plot comparison, reprinted with permission from Ref. [122]. **b** Electrospinning of MXene based fibers for supercapacitors electrodes, comparison of specific capacitance of different composite fibers, Cyclic stability of SMX/C under 10 K repeated CV tests at a scan rate of 100 mV s^−1^. Reprinted with permission from Ref. [123]

Two different approaches are employed to synthesize MXene based functional fibers to obtained potential energy storage function: (1) coating fibers and yarns (individual or shall around) with MXene-based active material such as nylon, polyester, and cotton [117–119]: (2) Another approach is to incorporate MXene-based active material in the structure of the fibers by mixing material in spinning solution formulation and synthesize customized composite fibers. In this approach, three different techniques can be used to synthesize composite fibers such as melt spinning [120], wet spinning [114], electrospinning [121]. He et al. [122] reported a synthesis of hybrid fiber structure, via wet-spinning technique, composed of reduced graphene oxide (rGO) and MXene, which are highly conductive 2D materials as shown in Fig. 13a. These fibers are used to assemble supercapacitors, and devices have exhibited electrochemical performance and energy density and power density of 9.85 mWh cm^−3^ and 7.1 W cm^−3^, respectively. Similarly, Hwang et al. [123] prepared a composite fiber of Ti_3_C_2_T_*x*_/carbon nanofibers for supercapacitors electrode exploiting electrospinning technique as shown in Fig. 13b. The device has shown excellent electrochemical performance with specific capacitance of 120 F g^−1^ and 98% capacitance retention of after 10000 charge and discharge cycles.

*Fiber Shape Substrate* Flexible and portable microelectronics, e.g., sensors and micro-cameras, have been developed for monitoring quality and production in industry, day-to-day life, defense, biomedical applications, and environmental nursing. These electronic devices need continuous energy and power supply to make them operational for real-time information and monitoring. The potential candidate for this continuous supply of energy to the devices is FSSCs and known as wire-shaped SCs [77]. FSCs, in addition to continuous supply of energy for flexible or portable electronics, demonstrated excellent potential for future intelligent textile. Thanks to technological advancement in weaving technology, appropriate yarn [the “suitability” regarding mechanical attributes, e.g., mechanical stability (flexibility and tensile strength)] can be converted into different clothes. These properties sustain the development and fabrication of wearable and intelligent textiles that not only have appropriate mechanical stability but also can store energy. Thanks to excellent energy storage capabilities of FBSCs, they can be directly integrated into textiles exploited traditional textile processes [124].

**Fig. 14:**
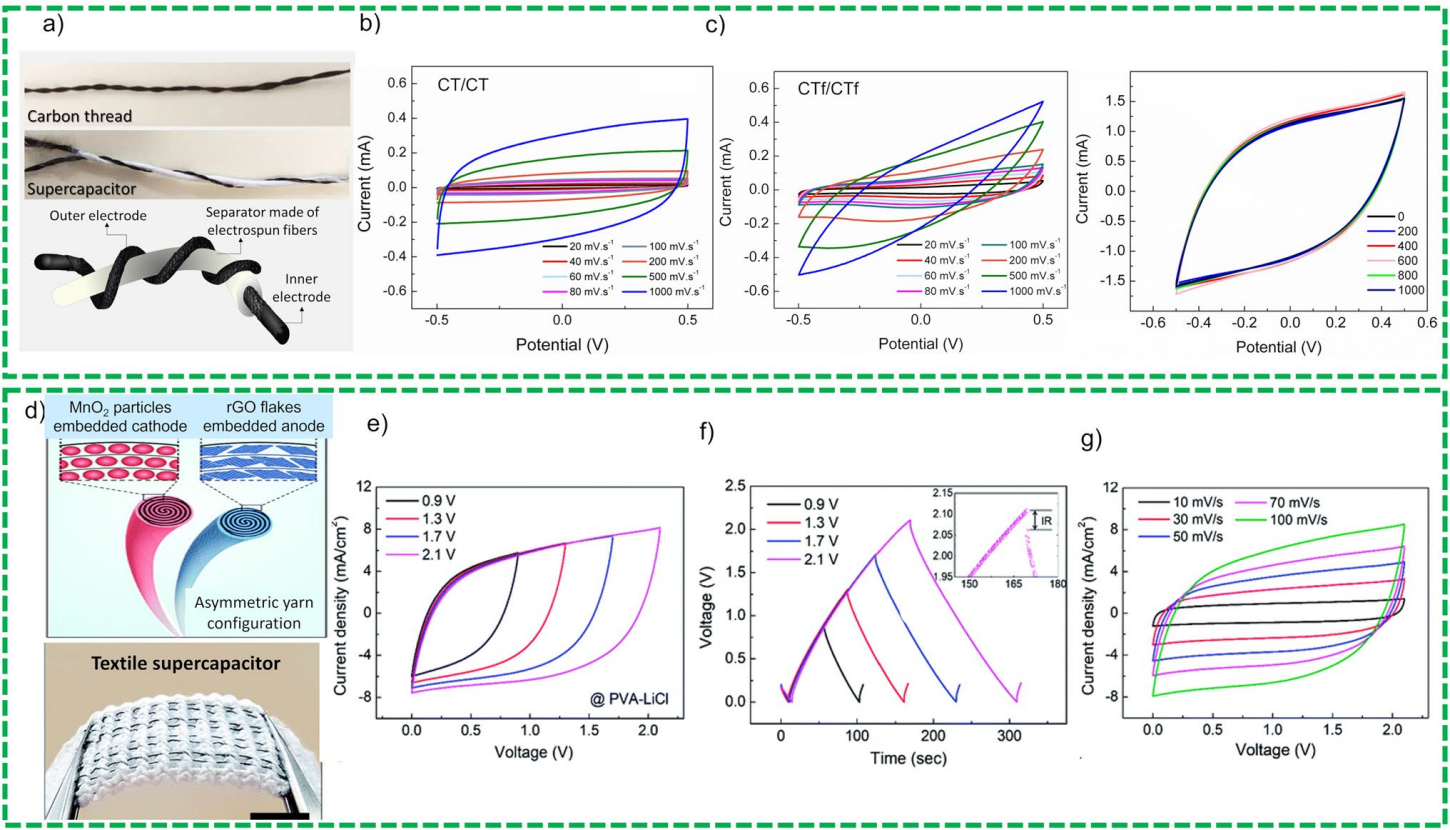
**a** Pictorial representation of the device in twisted configuration of carbon thread, **b** and **c** electrochemical performance comparison of pristine carbon fibers and polypyrrole functionalized carbon fibers at different scan rate, Reprinted with permission from Ref. [125], **d** schematic demonstration of asymmetric supercapacitors, **e** electrochemical performance in different potential windows and comparison of cyclic voltammograms on different scan rates, **f** charge and discharge comparison of the device at different potentials, **g** CV comparison at different scan rates. Reprinted with permission from Ref. [126]

Fibers-like substrates are used in two types of configurations as depicted in Fig. 14a. Configuration 1 is fabricated by coiling two fibers like electrodes around each other, and configuration 2 demonstrates a coaxial configuration: a fiber-like is used as current collector with inner electrode and outer electrode Fig. 14d. These electrodes are also known as film electrodes. Typically, during fabrication of these electrodes an electrolyte and a separator is sandwiched between these films depending on the type of electrolyte used. If aqueous electrolyte is used during the fabrication, then a separator is placed in between two films for assembly of the devices. But, in case of gel-polymer electrolyte such as PVA/Na_2_SO_4_, which offer dual functionality separator and electrolyte eliminate the need of separator to avoid short circuiting. Moreover, gel-polymer electrolytes are more diverse than aqueous electrolytes in FSSCs owing to the problem of leakage in later case.

Lima et al. [125] fabricated a symmetrical wire-based supercapacitors using wearer sweat as electrolyte as shown Fig. 14a. Here, they used polypyrrole activated carbon wire-based flexible electrode as inner and outer electrodes in simulated sweat solution as shown in Fig. 14b and compared the electrochemical performance of the devices with and without polypyrrole. The inner electrode was covered with electrospun cellulose acetate film, used as separator, and outer electrode coiled around this cellulose covered electrode. The device has shown a specific capacitance of 2.3 F g^−1^ and energy and power density of 386.5 mWh kg^−1^ and 46.4 kW kg^−1^, respectively. The device demonstrated excellent cyclic and bending stability under different mechanical stress levels as shown in Fig. 14c.

Similarly, Choi et al. [126] developed a carbon nanotube (CNT) yarn-based asymmetric supercapacitor using the liquid-state biscrolling technique for fabrication as shown in Fig. 14d. Here, they used rGO as anode and MnO_2_ as cathode and successfully incorporated them in CNT yarn for flexible electrodes. The electrodes were dip-coated with gel-based electrolyte (PVDF-HFP-TEA-BF4) which offers extended potential window up to 3.5 V and exhibited high energy density of 43 µWh cm^−2^ as shown in Fig. 14e and f. The devices were also characterized in parallel and series configuration, and they exhibited remarkable performance. The devices were also subject to stress level, and performance was evaluated and the device demonstrated excellent stability. Additionally, yarn-based electrodes showed excellent mechanical strength to be woven easily into textiles for commercialization.

*Polymer-Based Nonconducting Substrates* Polymer-based substrates offer many advantages such cost-effectiveness, comparatively light weightness along with excellent bending stability. Polyethylene Terephthalate (PET) [127], polydimethylsiloxane (PDMS) [128], polyimide [129] are considered promising substrates for active materials. Among these non-conducting polymers, PET is extensively exploited for energy applications due to its inherent properties such as excellent water and moisture repellency, easy accessibility, and transparency. Numerous studies have reported PET-based substrates for flexible SCs applications [130]. A transparent flexible SC composed of graphene as active material has been reported [131]. An energy storage device with an optical transmittance approx. 67% at wavelength of 500–800 nm has been synthesized which demonstrated considerable potential for transparent electronic devices applications. The devices assembled using these substrates as support to active materials showed a good bending stability and retained a capacitance of 93% of initial capacitance after bending it at 80°. The slight decline in capacitance retention was attributed to electrode material crumpling during compression. Choi et al. [132] presented a graphene-based FBSCs with a twofold increase in capacitance exploiting functionalized rGO(reduced graphene oxide) thin film as active material and Nafion membranes that offer a dual functionality electrolyte and separator. Moreover, a SCs device based on rGO cell demonstrated a higher specific capacitance as well as fourfold higher specific capacitance than that of rGO-based cells. This increased specific capacitance and improved performance of the devices were ascribed to excellent ionic transport at EDLC because of interfacial engineering of rGO and Nafion.

**Fig. 15:**
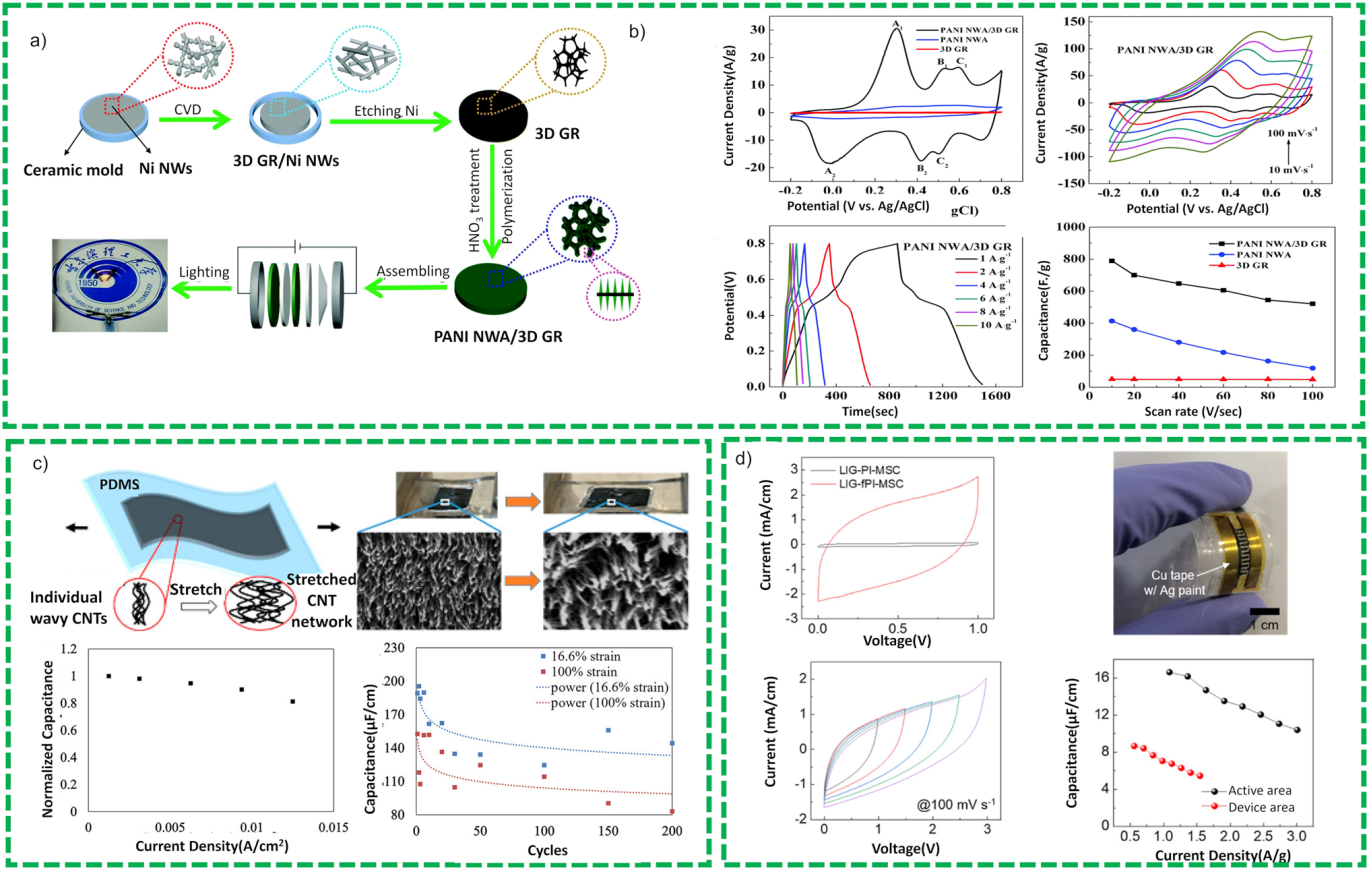
**a** Graphical representation of PANI/3D graphene electrode fabrication and then assembling in SCs device, **b** electrochemical characterization of the composite electrode CV, GCD, performance comparison, Reprinted with permission from Ref. [133]. **c** Graphical representation of fabrication process and demonstration of the shape change woven CNTs on PDMS under strain condition via APCVD, synthesized CNTs are moderately inserted in PDMS, adhesion of CNTs with PDMS in stretched conditions along with electrochemical characterization. Reprinted with permission from Ref. [134]. **d** Electrochemical characterization of both materials in 1 M H_2_SO_4_ at 0.005 V s^−1^, picture of pouch cell, stability in different voltage windows, specific capacitance as function of current density, cyclic and bending stability in different bending radius. Reprinted with permission from Ref. [135]

However, specific capacitance of graphene-based devices is still low and therefore, it hindered the exploitation of graphene thin films in a diverse range of applications. Zhang et al. presented a new strategy of synthesizing three-dimensional graphene exploiting chemical vapor deposition using Ni nanowires template as shown in Fig. 15a. PANI NWAs have been grown on this substrate surface via in-situ polymerization to form a composite referred as PANI NWA/3D graphene that possess small pore size which consequently offer reduced ion-diffusion path for electrolyte ions. The device has demonstrated an excellent electrochemical performance with 789 F g^−1^ at 10 mV s^−1^ as shown in Fig. 15b. The symmetric configuration assembled device has shown excellent energy and power density and retain 90% of initial capacitance after 5000 cycles.

Zhang et al. [134] prepared a highly flexible and stretchable SCs device comprised of interwoven CNT incorporated in PDMS substrate, as demonstrated in Fig. 15c. CNTs were synthesized using chemical vapor deposition (CVD) on glass substrates and then partially incorporated in the structure of the PDMS. The assembled device demonstrated an excellent electrochemical performance in 30% solution of KOH and PVA-KOH gel-polymer electrolyte. The device showed performance of 0.6 mF cm^−2^ with 30% of KOH solution and 0.3 mF cm^−2^ with simple PVA-KOH at a scan rate of 100 mV s^−1^. The device exhibited excellent stretchability of almost 200% and bending stability by twisting/twisting angle range of 0°–180°.

Polyimide (PI)-based polymeric current collectors are attracting substantial consideration for potential application in flexible SCs. Kim et al. [135] deposited a 3D nanostructure of laser induced graphene (LIG) on fluorinated PI using the photothermal technique and used for supercapacitors applications as displayed in Fig. 15d.

This LIG-based active material offers high specific surface area (1126 m^2^ g^−1^) which results in higher electrochemical performance and specific capacitance. The assembled device exhibited higher specific capacitance 110 mF cm^−2^ which is 27 times higher than current state of art micro-supercapacitors. Similarly, Peng et al. [136] synthesized a vertically aligned graphene on PI current collector fabricated by laser induced technique. The assembled device demonstrated a specific capacitance of 9.11 mF cm^−2^ at current density of 0.01 mA cm^−2^ along with remarkable cyclic stability with capacitance retention of 98% after 8000 cycles and bending stability after 7000 cycles bend at radius of 14 mm.

Several other polymer-based flexible current collectors have been fabricated and tested for FBSCs such as polyether sulfone (PES) [137], polycarbonate (PCs) [138] and Perylene [139]. Although current polymers substrates-based SCs are offering excellent performances in terms of capacitance portability, ion accessibility and flexibility they have limitation of electrical conductivity which has affected negatively SCs charge and discharge rate. Table 1 shows the list of numerous flexible substrates applied for the development energy harvesting devices and Table 2 shows the list of flexible substrates used for energy storage devices.

**Table 1:** Performance of textile-based energy harvesting devices

Classification	Materials	*V*_oc_ (mV)	*J*_sc_ (mA cm^−2^)	FF	Efficiency (%)	Refs.
Fiber shape OSC	Conductive polymers	607	11.9	53.8	3.87	[140]
	Conductive polymers	930	22.18	72.5	15	[141]
	Polystyrene templated TiO_2_ nanocrystalline film					[142]
	P3HT:PCBM/PEDOT:PSS	570	13.11	24	1.8	[49]
	P3HT:PCBM/PEDOT:PSS	560	8.5	0.46	2.2 ± 0.2	[66]
	P3HT:PCBM/PEDOT:PSS	500	0.24	3.44	0.4	[67]
Fiber shape DSSC	TCO free TiO_2_ film	707	5.07	0.676	2.4	[143]
	Oriented, crystalline ZnO nanowire array	710	5.85	0.38	1.5	[144]
	TiO_2_ nanowire array				5.38	[145]
	TiO_2_ nanotube array	670	15.46	0.648	6.72	[146]
	TiO_2_ nanocrystal film				7.19	[147]
	TiO_2_ micro cone array	702	16.036	0.717	8.07	[148]
	Hydrophilic and hydrophobic CNT	725	19.43	0.71	10	[28]
Fiber shape perovskite solar cell	Perovskite CH3NH3PbI3	664	10.2	0.487	3.3 95% retention after 50	[149]
	CNT fiber and Perovskite nanocrystals	615	8.75	0.564	3.03 89% efficiency retention after 1000 cycles	[27]
	Ti/c-TiO_2_/meso-TiO_2_/perovskite/spiro-OMeTAD/Au	713	12.32	0.609	5.35	[150]
	Carbon cloth embedded in carbon paste/Spiro-OMeTAD	1120	20.42	0.670	15.29	[151]
	PET/ITO-TiO_2_/meso-TiO_2_/perovskite/spiro-OMeTAD/Au	1150	23.36	80.25	22.68	[151]

**Table 2:** Performance of flexible energy storage devices

Category	Current collector	Material	Electrolyte	Performance	Refs.
Wire-based supercapacitors	Copper wire	ZnO/graphite	PVP/NaCl	Specific capacitance 27 F g^−1^, device retain 95% its initial capacitance after 500 cycles	[77]
	Copper wire	CuO	PVA/KOH	Specific capacitance (39.67 mF cm^−2^, 24.91 mF cm^−1^), device retain 51% its initial capacitance after 1000 cycles	[97]
	Stainless steel	CoOx	KOH	650 mF cm^−2^	[96]
	Stainless steel	TiO_2_–VACNT	Na_2_SO_4_	Specific capacitance 16.24 mF cm^−2^, device shown 99.7% capacitance retention after 5000 cycles	[101]
	Titanium wire Ti	carbon/TiO_2_ nanotube/Ti (CTNT)	PVA/LiCl	Areal capacitance of 121.42 mF cm^−2^, devices exhibited 76.5% capacitance retention after 10,000 cycles	[98]
	Titanium wire Ti	Nitrogen-doped graphene	5 M KOH	Specific capacitance 113.8 F g^−1^, device showed 95.2% capacitance retention after 4000 cycles	[102]
	Nickel wire	NiCo_2_O, Fe_3_O_4_	PVA/KOH	Specific capacity of 315.4 C g^−1^, device demonstrated 94.8% retention of initial capacity after 20,000 cycles	[152]
Paper-based supercapacitors	Carbon paper	Carbon Nanotubes and Polyaniline Nanowire Arrays	PVA-H_2_SO_4_	Specific capacitance 38 mF cm^−2^, device exhibited 91% capacitance retention after 800 cycles	[153]
	Carbon paper	nanostructured Polyaniline (PANI)	H_2_SO_4_	Specific capacitance 1106.9 ± 1.5 F g^−1^, device demonstrated 96% capacitance retention after 1000 cycles	[104]
Textile-based supercapacitors	Carbon yarn	Ppy/CF yarn	Simulated sweat solution	Specific capacitance 2.3 F g^−1^, 100% capacitance retention after 1000 bending cycles at 180^o^	[125]
	Carbon Nanotube Yarns	Co_3_O_4_ and NiO Nanoparticles	PVA-H_2_SO_4_	Specific capacitance 52.6 mF cm^−2^, capacitance retention 91% after 1000 cycles	[154]
	Cotton cloth-based substrate	FCC@PANI nanocomposite,	H_2_SO_4_	Specific capacitance 351 F g^−1^ at current density of 1 A g^−1^, 90% capacitance retention after 10,000 cycles	[108]
	Carbon fibers	MnO_2_	PVA/Na_2_SO_4_	Specific capacitance 63 F g^−1^, device exhibited 90% cyclic stability after 10,000 cycles and 95% bending stability	[1]
	Carbon fibers	rGO aerogel/Fe_2_O//MnO_2_	PVA/KOH	Specific capacitance 50 Fg^−1^, device exhibited 75% cyclic stability after 2500 cycles	[82]
	Carbon fiber	Mn_3_O_4_//Mos_2_	PVA/Na_2_SO_4_	Specific capacitance 56 F g^−1^, device demonstrated 80% cyclic stability after 3000 cycles	[81]
	Electrospun carbon nanofibers	MnO_2_/Co_3_O_4_ on electrospun CNFs	6 M KOH	Specific capacitance 728 F g^−1^ for MnO_2_/Co_3_O_4_/ECNF and 622 F g^−1^ for MnO_2_/ECNF at 5 mV s^−1^, device exhibited 72% capacitance retention after 11,000 cycles	[111]
	Conducting polymer-based substrate	CNT incorporated in PDMS	PVA-KOH	Specific capacitance 0.3 mF cm^−2^	[134]
		Laser induced graphene on PI	PVA-KOH	Specific capacitance 110 mF cm^−3^	[135]
		Vertically aligned graphene on PI current collector		Specific capacitance 9.11 mF cm^−2^ at current density of 0.01 mA cm^−2^, 98% cyclic stability after 8000 cycles and 7000 bending cycles	[136]

#### Flexible Batteries

The second potential source to meet the emerging demands of power supply is flexible batteries [155]. Confidence on operating systems in electronic gadgets such as mobile and electric vehicles has pushed for compact, lightweight, and flexible power solutions, which have potential to substitute plane, rigid and heavy batteries [156]. The evolution of flexible batteries dates back to almost hundreds of years ago. Evolution of flexible batteries started from flexible alkaline batteries [157] and full polymer batteries [158]. Later, polymer Li-metal batteries started to attract more interests [159]. Recently, investigators concentrated their focus on flexible Li-ion batteries with more superior energy density and cycling stability [160]. Flexible batteries share the similar working principle as of traditional batteries and comprises of an anode, a cathode, a separator, and an electrolyte. The traditional rigid battery and planar structures cannot meet the obligations of flexible electronics, and it is imperative to fabricate flexible batteries with unique features and attributes to integrate with robust flexibility while conserving high energy density and extended cyclic stability [161].

Significant progress in this aspect has already been made not only in the development of constituent elements (anode and cathode materials, current collectors, solid electrolytes, etc.) but also in design aspect of battery system. It is certainly critical, flexible batteries must hold a high flexibility and deformability for all the four crucial constituent elements namely cathode, anode, electrolyte, and separator, to guarantee their steady power supply even under mechanical stresses [13]. Flexible Li-ion batteries are the most extensively used battery systems for flexible and wearable electronic devices, but other battery chemistries including low-cost and wide accessible, e.g., sodium-ion batteries (SIBs) [162], potassium-ion batteries (PIBs) [163] and high energy density lithium-metal batteries (LMBs) [164], lithium–sulfur batteries (LSBs) [165] and metal-air batteries (MABs) [166] have also been aggressively researched to inflate the scenery of flexible batteries in recent past. Flexible batteries have been fabricated in three different configurations such as 1D [156] fiber-shaped, 2D and 3D architectures.

##### Constituents of Battery Devices

Flexible battery, like traditional batteries, is also comprised of four essential constituent elements, i.e., an anode, a cathode, a separator, and an electrolyte. However, the rigid and delicate nature of these constituents’ elements for traditional batteries would trigger failure and even safety concerns under mechanical stresses [13]. To ensure the sustainability and mechanical stresses for flexible batteries, it is imperative to fabricate flexible constituent elements. Remaining in the scope of this review paper, we are focusing on flexible current collectors.

##### Current Collectors

Current collectors perform a vital role in the fabrication of flexible electrode because it serves as both the substrate to assist active materials and help to connect the electrodes to complete the circuit. An ideal flexible substrate for fabrication of flexible battery electrodes must have certain attributes such as high flexibility, low-weight, excellent electrical conductivity, and solid adhesion with active materials. We will discuss in detail different types of flexible substrates used for flexible batteries.

##### Metallic Foils Substrates

**Fig. 16:**
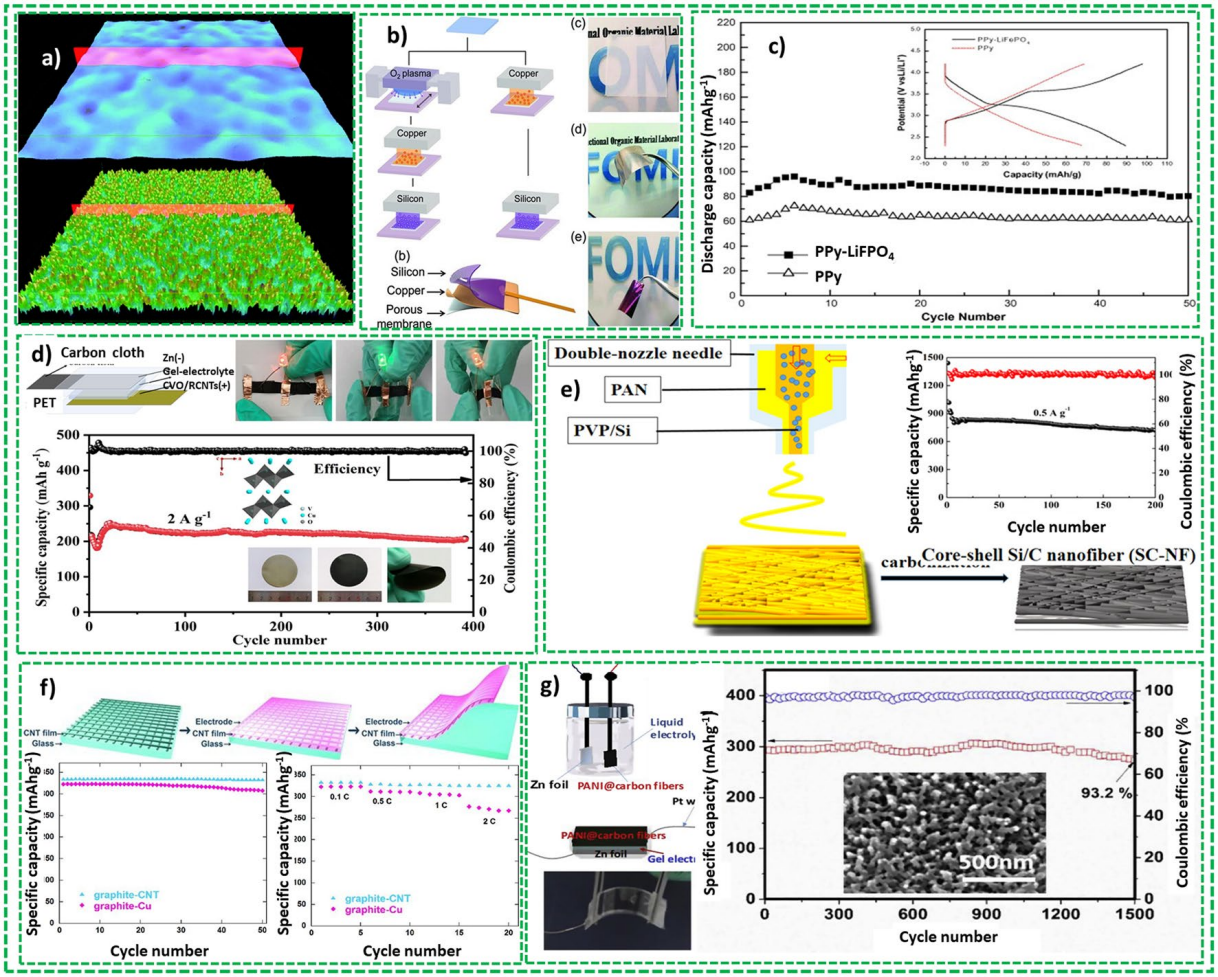
**a** Surface morphology of a rough cupper substrate. Reproduced with permission from Ref. [175]. **b** Demonstration of multilayer silicon electrode fabrication. Reproduced with permission from Ref. [176]. **c** Cyclic stability comparison of PPy and PPy-LiFePO_4_ deposited material, inset illustrates the discharge/charge curves of the 10th cycle Reproduced with permission from the reference Ref. [177]. **d** Graphic diagram of flexible gel Zn//CVO-18/RCNTs battery, demonstration of two flexible gel Zn//CVO-18/RCNTs batteries in series to light up a 1.5 V led bulb at different bending states and cyclic performance of the battery. Reproduced with permission from Ref. [178]. **e** Diagram of synthesis of the SC-NF composites using electrospinning technique and cyclic performance of the battery. Reproduced with permission from Ref. [179]. **f** Graphic illustration of the method of making flexible electrodes with SACNT films working as lightweight and thin current collectors, Cycling performance of battery at (0.1C) and rate performances of the graphite-CNT and graphite-Cu electrodes. Reproduced with permission from Ref. [180]. **g** Schematic illustration of 3-electrode setup for electrochemical performance of PANi grown on carbon fibers, digital image of ZIB and cyclic performance of ZIB at 4 A g^−1^ (the inset SEM image after 1500 cycles) Reproduced with permission from Ref. [181]

Metallic foils owing to their high electrical conductivity have been used as current collector for fabrication of the battery’s electrodes in the traditional batteries for years. Thanks to the flexibility of these foils, it is a simple approach to employ these current collectors to fabricate electrodes for flexible batteries. In this regard, variety of metallic foils have been exploited such as Al [167], Cu [168], Ti [169], stainless steel [170] and Li [171] to fabricate electrodes for flexible batteries. One of the problems with these metallic current collectors is the delamination of the active materials under mechanical stresses and deformations owing to their rigidity and delicacy which sometime leads to their poor battery performance. To counter this problem, researchers have devised a new strategy in which they make the current collector surface rough to increase the contact points and surface area for active material layer and their adhesion with the substrate. To counter this problem, many techniques have been adopted to introduce roughness on surface of the current collector such as sandpaper grinding [172], chemical etching of the substrate [173], and special morphology designing of the substrate [174] as shown in Fig. 16a. However, if a mismatch exists between current collector and active material during charge/discharge cycles, can intensify delamination of electrode materials from the metallic substrate especially under repeated mechanical deformations. In addition to bulkiness and low specific performance as well as delicacy and rigidity for metallic current collectors could inexorably impede the realization of flexible batteries. This has motivated researchers to explore other flexible and lightweight substrates, such as polymeric and carbon-based flexible materials, which has improved the adhesion of active material.

##### Polymeric Substrates

Polymers substrates with built-in attributes such as flexibility and lightweight attracted researcher attention as flexible current collector for flexible batteries [182]. In this aspect, both conducting and insulating polymers have been explored as flexible substrates for flexible batteries. Different types of insulating polymers have been explored such as polyimide (PI), poly(ethylene terephthalate) (PET), poly(ethylene naphthalate) (PEN), poly(ether sulfone) (PES), and polypropylene (PP) which can sustain mechanical stress and deformation without fractures and deteriorating electrochemical performance. However, these substates have an obvious problem of low electrical conductivity which limit their use as flexible current collector. To address this problem, substrates are metallized before their use as current collector and deposition of active materials [183]. Choi et al. [176] and Yun et al. [184] employed magnetron sputtering technique for Cu coating on PI & PEN, PET and PI, respectively, to increase the conductivity and the substrates demonstrated a surface resistance of 1 Ω cm^−1^ with coating thickness of < 1 µm. The silicon nanofibers were sputtered on Cu coated current collectors and as shown in Fig. 16b. The pouch cell assembled from this silicon-coated anode and Li foil cathode demonstrated a high capacity of 2000 mAh g^−1^ and coulombic efficiency of 97% after 80 cycles at a bending radius of 1.9 cm which shows the viability of insulating polymers as flexible substrates in the battery applications [176]. The application of these insulating polymers bears an additional cost of metallization which may hindered their use as flexible substrates.

Other than insulating polymer, electrically conducting polymers such as polypyrrole (PPy), Polyaniline (PANi), and polythiophene (PTh) are naturally conductive and can directly be employed as flexible current collector without metallization [185]. Thanks to their electroactivity, they can be employed as free-standing electrode without loading an external active material. Numerous studies [177, 186] have been reported in which conducting polymers were used either as free standing or as substrate to load other active materials for flexible batteries. Wang et al. [177] fabricated a device by electrochemically preparing a PPy and PPy/LiFePO_4_ composite electrode and assembled devices exhibited a capacity of 60 and 80 mAh g^−1^ as shown in Fig. 16c. The higher performance attributed to LFP constituent in the composite material, but their practical application still impeded owing to their chemical volatility and comparatively low conductivity as compared to carbonaceous materials [187].

##### Carbon Materials

Numerous carbonaceous materials have been studied as novel current collector for flexible batteries such as carbon nanotube (CNT) [188], graphene [189], carbon paper [190], carbon foam [191], carbon fibers [192], and carbon cloth [193] owing to their excellent electrical conductivity, mechanical strength, flexibility, and chemical stability. Different fabrication techniques have been used for the preparation of the electrodes and loading of active material on carbonaceous materials such as vacuum filtration, solution-casting, slurry-coating, solvothermal method, and melt-diffusion [185].

Song et al. [178] employed vacuum filtration technique to fabricate a freestanding CuV_2_O_6_/reductive acidified CNTs (CVO/RCNTs) composite film for ZIBs as shown in Fig. 16d. The device has demonstrated a remarkable reversible capacity of 174.7 mAh g^−1^ and capacity retention of 61.5% after 1400 cycles at 5 A g^−1^. The device prepared with gel-polymer electrolyte has exhibited stable electrochemical properties under mechanical stresses and bending degrees displaying great potential for flexible batteries. Similarly, Li et al. [179] fabricated core–shell silicon/carbon (Si/C) composite-based anode materials for lithium-ion battery using an electrospinning technique as shown in Fig. 16e. The fabricated battery device demonstrated specific discharge capacity of 1441 mAh g^−1^ with almost 77% of capacity retention at 0.5 A g^−1^.

Slurry-coating is the most used technique in battery industry that has been a steerable procedure to fabricate flexible electrodes on carbon substrates. Wang et al. employed this approach for LIB to load graphite and LiCoO_2_ (LCO) slurries on CNT films as anode and cathode, respectively, as shown in Fig. 16f [180]. The fully fabricated battery device comprising of LCO-CNT film cathode a LiPF_6_-EC/DEC electrolyte and Celgard 2400 separator, the graphite-CNT film and graphite-Cu anodes demonstrated a capacity of 335 and 318 mAh g^−1^ at 0.1C after 50 cycles, respectively. Similarly, Li et al. fabricated ultrahigh-energy cathode of PANI nanopillars developed on surface cracked carbon fibers as shown in Fig. 16g [181]. The device has demonstrated a very high capacity of 412.7 mAh g^−1^ at 0.5 A g^−1^ and excellent cyclic stability with capacity retention of 93.2% after 1500 cycles at 4 A g^−1^.

It is evident from the above arguments, metallic, polymeric, and carbonaceous materials have been investigated as flexible current collector for flexible batteries. Some advantages and disadvantages of the representative substrates discussed above are summarized in Table 3. It is evident from the table that these substrates possess diverse attributes and minuses, suggesting the attributes for an ideal current collector. Carbonaceous current collector has invited significant interests and will continue to play a crucial part a novel substrate for flexible batteries owing to possession of distinct attributes such as lightweight, flexibility, mechanical and chemical stability.

**Table 3:** Characteristic of metallic, polymer and carbonaceous substrates

Flexible substrate	Advantages	Disadvantages	Refs.
Metallic substrates	Excellent electrical conductivity	Bulkiness	[185]
	Good compatibility with established industrial practices	Rigidity/ inflexibility	
		Disparity in volume shift with active material	
Polymeric substrates	Low weight	Poor adhesion of active material	[182]
	Excellent flexibility of substrate	Chemical volatility	
	Cost-effective	Poor electrical conductivity	
Carbonaceous substrates	Low weight	Complex development process	[194]
	Excellent electrical conductivity	High cost	
	High flexibility		
	Remarkable chemical stability		
	High mechanical stability		

### Sensing Devices

The primary task of sensing devices is to detect external stimuli into an electronic signal such as changes in temperature, humidity level, environmental changes, and mechanical deformations to acquire specific information. The sensing devices are divided into three broad categories based on their working principle, physical sensor, chemical sensors, and electrophysiological sensors. In the recent past, flexible sensing devices have attracted broad interest as compared to their counterpart traditional bulky electronic sensors owing to demonstration of superior conformability with dynamic and irregular surfaces and can easily be integrated into textile [195]. Recently, the concept of flexible fiber-based sensors have been established and also employed in different practical applications ranging from basic healthcare monitoring to clinical diagnostics of patients [196].

**Fig. 17:**
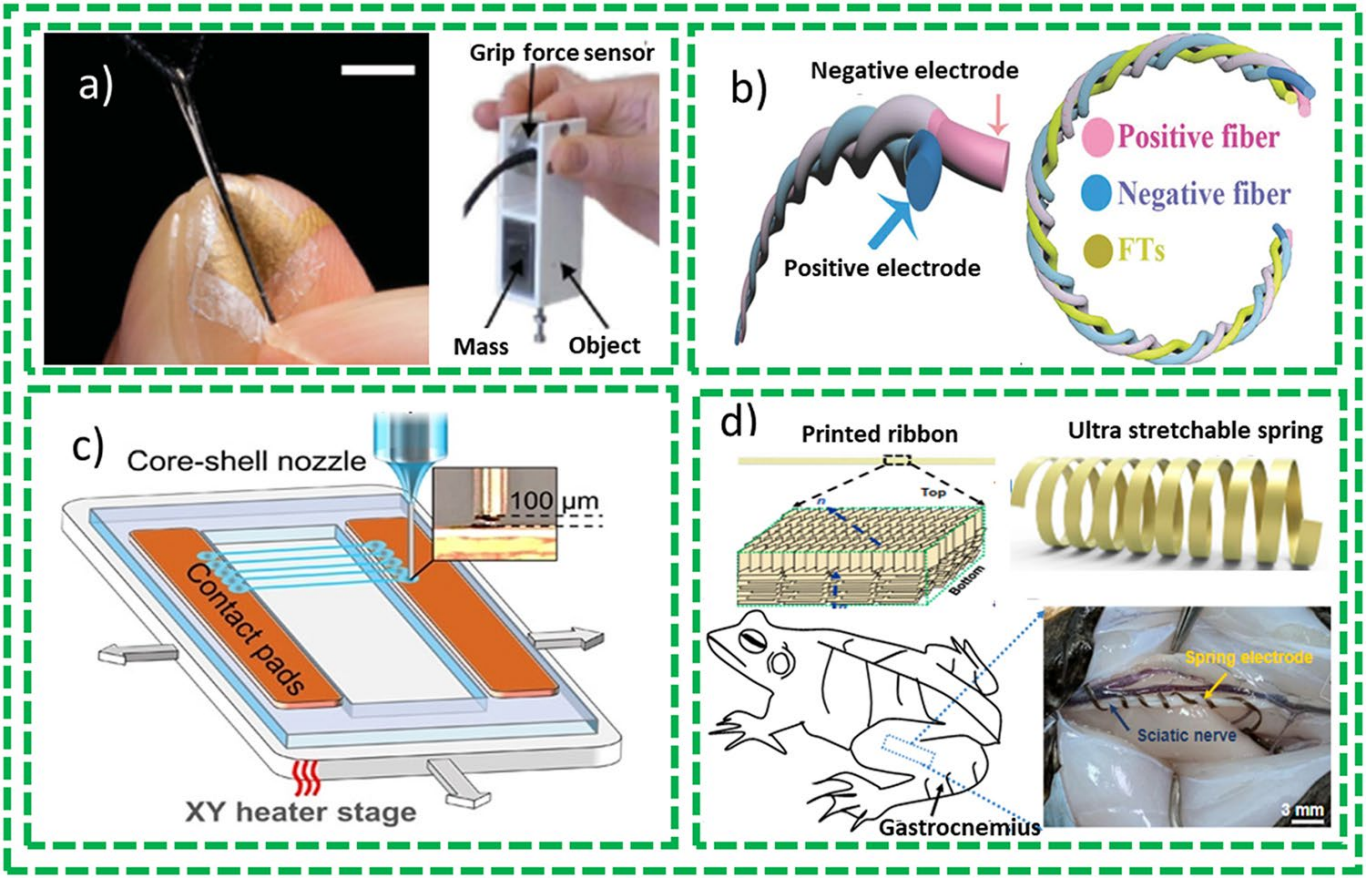
Fiber-based wearable sensing devices. **a** Nanomesh flexible pressure sensor for observing fingers control without sensory intervention reprinted with permission from Ref. [197]. **b** CNT fiber-based temperature sensors Reprinted with permission from Ref. [199]. **c** Inflight fiber-based 3D printed chemical sensors Reprinted with permission from Ref. [200]. **d** GO/SA micro-ribbon comprises of direct ink-based nanoflake-based ionic neural electrodes Reprinted with permission from Ref. [201]

The physical sensors, owing to their microstructures and inherent conductive properties, translate the external changes such as mechanical deformation or environmental stimuli into voltage/current or resistance/capacitance. Conventional sensor and even ultra-thin sensing devices can diminish the feeling of touch to some point, when they are mounted on human body. This ultimately results in the deviation of measured data and impotent to accurately reflect the natural sense of the skin. Recently, fiber-based stress/strain sensing devices were widely investigated and remain the most widely fabricated sensing devices because of their wide range of applications for physiological response monitoring such as blood pressure, heart rate, respiration rate [197]. Lee et al. [198] fabricated a FSCs pressure sensor applying electrospinning technique which can correctly monitor the finger pressure without noticeable impact on body feeling as shown in Fig. 17a. Similarly, Zhao et al. [199] developed a printed fiber-based temperature sensor and asymmetric FSC using 3D printing technique. The FSC could supply uninterrupted power to fiber-based temperature sensors and device exhibited a temperature responsivity of 1.95% °C^−1^ as shown in Fig. 17b. Wang et al. [200] functionalized the fiber conducting polymer with PEDOT:PSS to achieve conductive fibers to fabricate a portable respiratory sensor which can collect both the dispersal and direct the emanated gases as demonstrated in Fig. 17c.

In addition to observing physiological responses, uninterrupted nursing of biological and chemical parameters (e.g., electrolytes, metabolites and other biomarkers) in the body liquid is also vital to obtain broader picture of an individual’s health at the molecular level. Fiber-based chemical/electrophysiological sensing devices have been fabricated which can translate the characteristics and concentrations of different chemicals into electrical signals. Wang et al. [202] fabricated CNT fibers in coaxial configuration by depositing different active materials on the surface of CNT fibers which can real-time monitor the physiological parameters such as glucose, Na+, K+, Ca+ and pH and sustain the physical integrity and detection capability during reiterating mechanical deformation. Lately, fabrication of fiber-based implantable sensing devices have also been reported into living organisms to observe health response signals around the clock. Zhang et al. [201] used a direct-ink writing technique to fabricate a helical ionic neural electrode with ultrahigh stretchability almost 1000% and nanoscale ionic transport attributes. The device has demonstrated excellent compatibility with soft and active biological muscles as shown in Fig. 17d. Nevertheless, a great advancement has been accomplished, fiber-based chemical/electrophysiological sensing is still confronting challenges, such as easily drifted output signs, necessitating calibration before usage each time, unbalanced for long-term storage and use, restricting their everyday applications.

### Light Emitting

Other functional electronic fibers which have attracted immense attention of researchers are, Luminescent electronic fibers and played vital role in different fields such as transport, safety, anticounterfeiting, apparel and aeronautics [197]. Fiber-based luminescent electrical devices can be impeccably incorporated in textiles, permitting applications in flexible display, sensors, and mask. Incorporation of fibers into textiles is anticipated to amplify the human–machine interactions [203]. Different types of fiber-based LED electronic devices have been fabricated employing organic LEDs (OLEDs), light-emitting electrochemical cells (LECs) and phosphorescent electroluminescent devices [8].

**Fig. 18:**
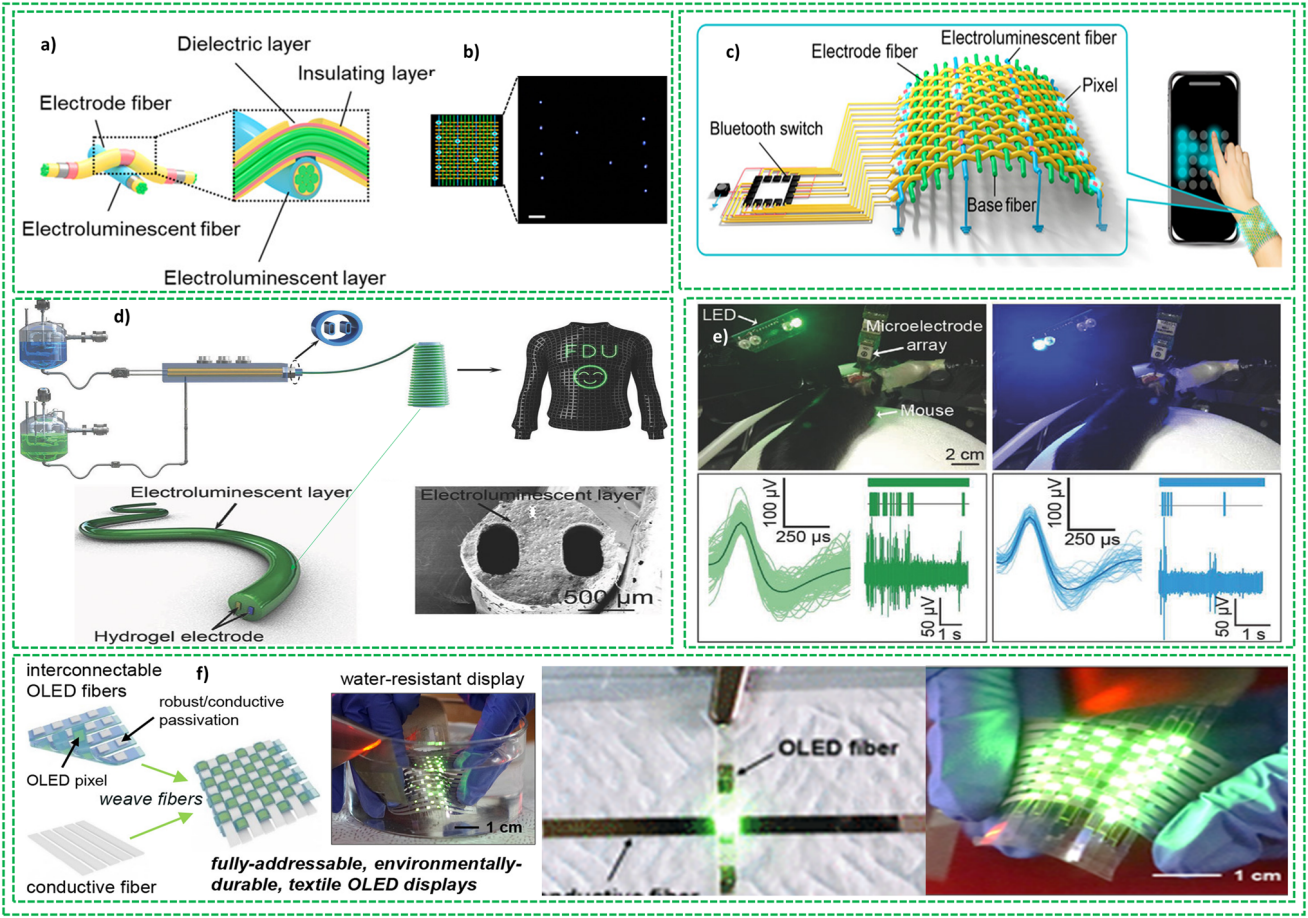
**a** Schematic graphic of a pixel configuration in the electroluminescent fabric. **b** Diagram and picture of the electroluminescent fabric displaying the design “N.” **c** Representation of the smart electroluminescent fabric being functionalized by connecting Bluetooth. **a**–**c** Reprinted with permission from Ref. [204]. **d** Graphics representation of the instantaneous extrusion process and the light-emitting fiber. **e** Real-time brain-interfaced camouflage of the SEF under green and blue illumination. Top: Setup for recording neural activity in mouse. Middle: Color responses of a V1 neuron. Bottom: The electroluminescent textile controlled by the decoder light up in the same color as the exposed light Reprinted with permission from Ref. [20]. **f** Pictorial demonstration of the woven OLED textile display comprising of orthogonally organized arrays of interconnectable OLED fibers and conductive fiber, OLED fiber perpendicularly placed to a conductive fiber for activating one-pixel, digital photograph of the working woven textile device comprising 10 × 10 fiber arrays by the passive matrix scheme Reprinted with permission from Ref. [205]. (Color figure online)

Mi et al. [204] fabricated an ultra-stretchable light-emitting fibers (up to 400% stretching) that can demonstrate a pixel-based controllable light-emitting pattern as shown in Fig. 18a. A dip-coated Zin sulfide (ZnS)-based luminescent layer and insulating layer were sandwiched between two liquid metal-coated electrodes (eutectic gallium-indium EGIn). The cross-sectional node of ZnS-coated fiber and EGIn-coated fiber function as luminescent pixel that develop a luminescent cloth matrix upon weaving as demonstrated in Fig. 18b. The pixel pattern can be achieved by employing DC and AC converter to Bluetooth switch in the electrical circuit as shown in Fig. 18c. Similarly, Zhang et al. [20] synthesized a continuous electroluminescent fiber employing a one-step extrusion technique as shown in Fig. 18d. The exterior ZnS nanoparticle along with silicon elastomer as a shielding layer was instantaneously extruded with two internal parallel hydrogel electrodes. The fibers have demonstrated excellent luminescence recoverability until 300% of stretch and maintained after 100 stretching cycles. These types of fibers are weave into stretchable fabrics to create display pattern and employed to execute a brain-interface camouflage system as shown in Fig. 18e.

Despite the advancement, fabrication and development of flexible displays made of luminescent fibers still need to be accomplished to attain high electroluminescent activity, strong interconnection of light-emitting fibers and long-lasting passivation layer. Song et al. [205] devised a viable approach to address the above problems for OLED textile displays that composed of interconnected phosphorescent OLED fibers and polyurethane (PU) based passivation layer as shown in Fig. 16f. The green OLEDs arranged in 1D pixel array were thermally loaded onto indium tin oxide (ITO)/Polyethylene terephthalate (PET) rectangular fibers with patterned PU side barrier. The device has exhibited a high brightness of 4300 cd m^−2^ at voltage of 5 V and an efficiency of 46 cd A^−1^. Although significant progress has been evident in this field but more research on light-emitting fibers required to achieve low diameter, 360° light emissions, and an easy industrial process for functional wearable displays.

## Economy of Scale for Flexible Electronic Devices

### Standard Configuration of Fiber-Based Electronics

**Fig. 19:**
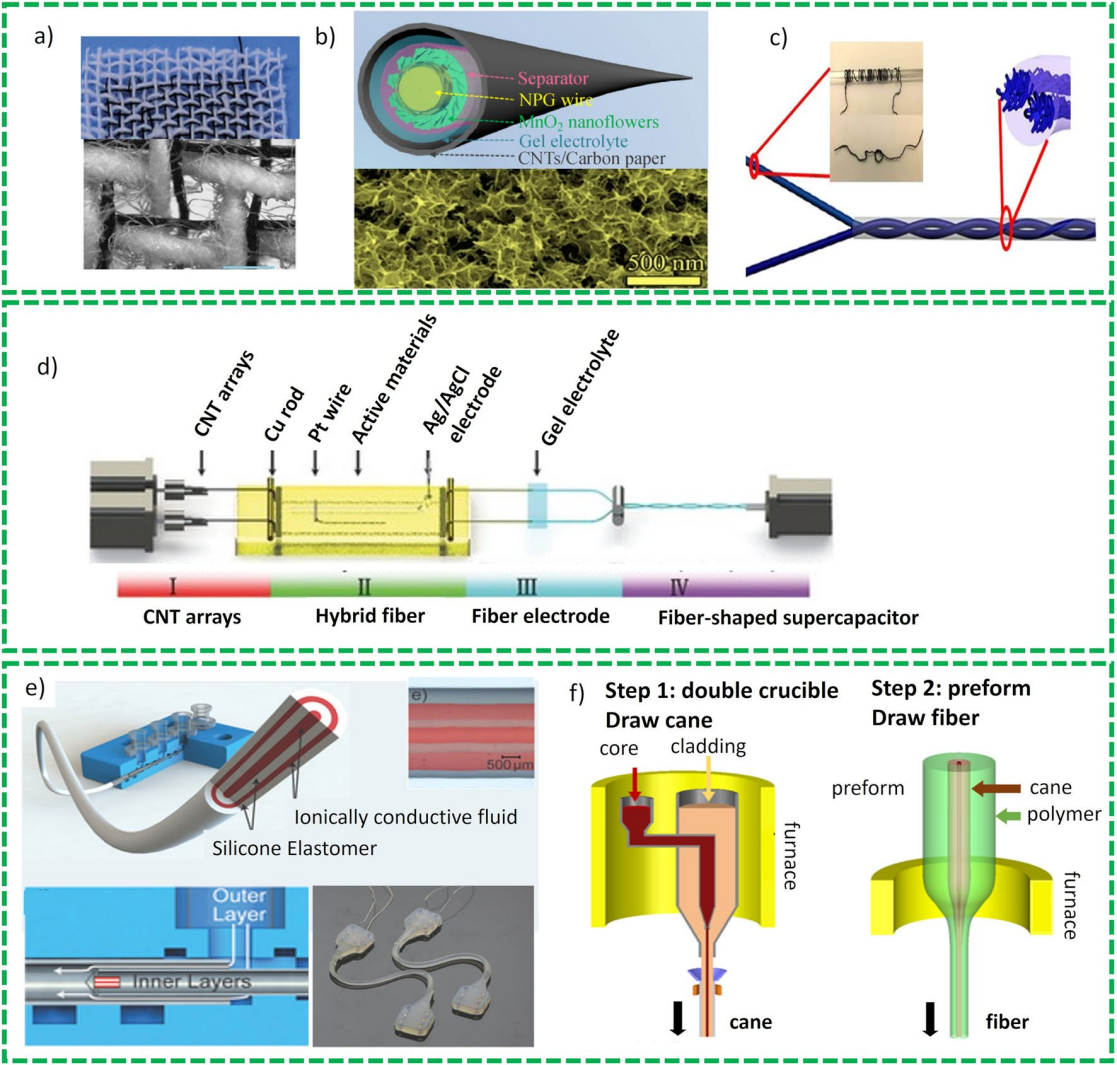
**a**–**c** FSEDs with different configurations, twisted, coaxial and interlaced or woven. **a** Reprinted with permission from Ref. [207], **b** Reprinted with permission from Ref. [208], **c** Reprinted with permission from Ref. [209]. **d**–**f** Large-scale production techniques for deposition, spinning, and thermal drawing techniques, **e** Reprinted with permission from Ref. [210], **f** Reprinted with permission from Ref. [211]

The current electronic devices are fabricated into three different shapes and assembled into different configurations such as twisting, coaxial and interlaced configurations as shown in Fig. 19a–c. Figure 19a shows that in fiber-shaped configuration electrodes are directly twisted or coiled around each other at specific angles and can be fabricated separately. The twisted electronic devices are usually encapsulated with some protected material to avoid the damages to the devices. However, twisted configuration of electronic devices still has some shortcomings such as, in case of photovoltaic devices, the wrapped and coiled secondary flexible electrode produced shade on the first electrode and block the light accessibility to this electrode, which consequently deteriorated the performance of this optoelectronic device [206]. The twisted configuration of the electronic devices is liable for generation of internal stresses. This stress will compromise the mechanical stability and constituents’ electrodes may drift and cause damage to the device during bending and twisting or wrapping buckle.

Compared to the twisting configuration where fabricated flexible electrodes are assembled in a parallel structure, the coaxial configuration displays a core-sheath structure. This configuration presents different advantages such as shorter path length for electrolyte ions transportation and better sustainability against daily life mechanical stresses [212]. Carbon nanotubes thin film, with optical transparency of 80–85% in wavelengths of visible range, can be used as a counter electrode in DSSCs. CNT deposited film guaranteed full contact with primary film of active material as well as consistent radiance along circumference through FBDSSCs.

Recently, a new configuration has been explored for fabrication of fiber-shaped electrodes: the interlaced configuration especially in photovoltaics [47]. Compared to twisting and coaxial configurations, the interlaced woven photoanodes and counter electrodes offer a fabrication of devices in form of single layer fiber electrodes as shown in Fig. 19c. In this configuration, holes are generated at active film due to photon excitation transported to photoanode and electrode transported to cathode fibers through cross interface. Therefore, it is feasible and economical to develop electronics textiles at the appropriate scale of meters by industrial loom.

### Bulk Fabrication and Production of Fiber-Based Electronics

Currently, numerous flexible electronic devices using interlaced [207], coaxial [208], and twisted [209] configuration are fabricated and tested extensively by researchers mainly at laboratory scale. The economy of scale and industrial commercialization of these laboratory scale electronic devices are still posing some challenges during fabrication and development, such as synthesis of fiber substrate-based electrodes, loading of active materials, the penetration of electrolyte ions, the device assembly and packing or encapsulation of these devices to avoid damages. The complicated procedures and time-consuming treatment limits commercialization of fiber-based electronic devices. Although latest and state-of-the-art instruments and development techniques are evolving for flexible electronics, there is still a lot to do for easy transformation and fabrication of flexible fiber-based devices [213].

Numerous approaches have been adopted to accomplish the continuous fabrication of fiber electronic devices. An integrated deposition approach has been developed for uninterrupted fabrication of FSSCs by synchronizing all above-mentioned procedures, as demonstrated in Fig. 19d. In this approach, a steady potential was maintained on pristine CNT fibers to set up an integrated electrochemical system. Through this procedure, GO sheets were loaded on CNT fibers and reduced to graphene synthesizing graphene wrapped CNT composite. After electrode fabrication, these composite electrodes were passed through the gel-polymer electrolyte, which will act as separator as well as electrolyte and prevent the short-circuiting during coiling with mechanical instruments. This integrated approach was extensible and possessed broad scope of applicability to other active materials such polypyrrole (PPy), MnO_2_ and Polyaniline. Additionally, this approach and technique is compatible with different flexible substrates such as metal wires, metal coated manmade fibers, and carbon fibers to produce FSSCs [210].

Coaxial configuration of fabrication of flexible electronics offer significant improvement exploiting wet spinning of graphene coated with gel-polymer protective film for uninterrupted fabrication of composite fibers, that are directly used as flexible electrodes to assemble FSSCs [214]. This configuration of co-spinning technique offers a valuable approach for direct production of flexible fiber-based electronic devices. Then, a typical multichannel spinning technique was suggested for uninterrupted production of coaxial FSSCs via inventing a particular spinneret with coaxial three outlet formation. During the fabrication process via wet spinning, active material and gel-polymer electrolyte are concurrently squeezed out into a coalescence bath. The electrolyte coating instantly dries out to protect the inner active material film and also acts as a separator between the inner and outer electrode. The diameter, thickness of the film and homogeneity of the electrode or gel-polymer electrolyte can be controlled by changing the concentration and viscosity of the material along with the extrusion speed [213]. Moreover, this technique can be updated to fabricate more complex flexible electronic devices such as the capacitive soft stress sensor device. Four alternate coatings composed of nonvolatile ionic liquids and silicone elastomer were combined and extruded simultaneously in circular alignment, applying printhead comprising of four cylinder-shaped needles supported coaxially as demonstrated in Fig. 19e. The position and constraints of these nozzles can be modified to control the thickness of each coating of the fiber sensor, facilitating personalized properties of the sensors [215].

3D printing has recently been considered as an advanced production technique and attracted significant attention owing to its high production effectiveness, synchronous fabrication, economy of scale and difficulty potential. 3D printing has been used in traditional electronics, such as SCs, sensors and batteries. Another advanced technology that is important for fiber processing is thermal drawing which was primarily employed for manufacturing of optical fibers and can also be exploited for polymers and active materials [216]. Lately, this technique is exploited as a new approach for fabrication of multifunctional fiber-based electronics. In this technique, all the reagents are first assembled into a single rod in predefined pattern and then, direct co-drawing for fabrication of fiber shape devices as shown in Fig. 19f. This technique has special prerequisites: (1) the component must be glassy to sustain drawing stress and physical integrity: (2) The components which tolerate drawing force exhibited softening or melting points above drawing temperatures: and (3) the component must display excellent adhesion properties in different states without delimitation.

### Encapsulation

One of the main limitations of the functional materials (organic or inorganic) in the fiber-shaped electronics is that they are volatile to react with water and oxygen which leads to device malfunction. For the effective utilization and long cyclic life, these fiber-shaped electronic devices must be protected using suitable packing or encapsulating layer outside the functional materials to avoid their direct exposure to moisture and oxygen. The sensitivity of these fiber-shaped electronic devices toward moisture and oxygen varies and the effect of encapsulation materials also varies according to active materials. Two different techniques are used to characterize the packaging materials such as water vapor transmission rate (WVTR) and oxygen transmission rate (OTR).

Encapsulation of the fiber-shaped devices have visible impact as it enhances the service life of the device by 1000 h and storage capacity life increases up to 5000 h. To avoid the deterioration of the functioning of the fiber-shaped electronic devices, WVTR should not exceed 0.01 gm^−2^ day^−1^ as for normal devices and for very sensitive electronic device it must be less than 1 µ gm^−2^ day^−1^ [217].

To enhance the life of fiber-based electronic devices, which are sensitive to oxygen and moisture, must be protected exploiting encapsulation technique which may affect the flexibility of the devices. Contrasted with conventional inflexible packaging materials, such as ceramic, and metallic packaging, polymer wrapping materials offer some advantages such as high flexibility, easy processing and cost-effectiveness and are widely explored as packaging coating in the pharmaceutical and food industries. Polyethylene, Polypropylene, PVDC poly (vinylidene chloride), PEN and PI are the most used polymers, but these polymers have limitations because they have WVTR in the range of 0.1–100 g m^−2^ day^−1^ which hinders their usage in these industries.

These problems can be encountered using polymer/inorganic composites which effectively reduces the WVTR without compromising the flexibility through sol–gel, melding, in-situ polymerization and intercalating methods [8]. By using these composites, WVTR can be restricted to 0.01–0.1 g m^−2^ day^−1^. The addition of nanoparticles in the polymer matrix will not only help to reduce the amount of polymer in the matrix which absorb the water vapor but also stretch the infiltration channel of water molecules to lowered diffusion rate potential permeable particles and enhance packaging materials efficiency. Constraints exist on the uniform distribution of nanoparticles into polymer matrix which often agglomerate during polymerization and on the transparency of the packaging material which limits its application as encapsulation coating for FBEDs. By using CVD (chemical vapor deposition) or PVD (physical vapor deposition), ceramics or metal can be deposited on the packaging surface which restrict the WVTR to Iµ g m^−2^ day^−1^. However, these techniques are too expensive, and the deposition rate is slow.

In daily life, fiber-shaped electronic devices are prone to many mechanical stresses and compression. Inorganic packaging materials are prone to penetration of water vapors which create cracks in the packaging materials and compromise its performance. To avoid this problem, multilayer composites are synthesized using both organic and inorganic materials in which an organic layer is incorporated to reduce cracks in the packaging material and an inorganic layer to prolong the permeability path and reinforce the packaging efficiency [217].

The flexible packaging materials for flexible electronics are still in early stage of development. At laboratory scale, thermal tubes are used for encapsulation of the devices and consequently devices which limits to centimeters its length and hindrance industrial production [218]. Considering the recent growth in demands for flexible electronic devices, new materials for packaging technology especially for fiber-shaped electronic devices are needed to accelerate the commercialization and industrial production of the electronic devices.

## E-Textiles

Electronic textile (E-textiles) embedded with diverse functionalities has applications ranging from energy harvesting, energy storage to sensing and display devices. These can be ascribed as the ultimate shape of wearable electronics. Presently, integration of electronics devices into textiles is classified into three broad categories: (a) embedment of simple device on textile substrate: (b) direct fabrication of flexible electronic device on textile substrate: and (c) integration of fiber-shaped electronics into textile fabric. For a practical applications and commercial point of view, the third approach will be mainly explored and discussed here.

### Development of E-Textiles

Compared with traditional electronic devices available in the market, Fiber-Based Electronic Devices (FBEDs) offer advantages such as softness and deformability. The construction of the textile substrate can be remodeled into enormous, customized patterns with a high degree of integration. The present fabrication instruments and technology failed to meet the requirements for the production of E-textile. To some extent, conventional textile processes, such as spinning, weaving, knitting and textile processing, have been proved as valuable strategies. Some innovation in the above traditional machineries will further boost the productivity for bulk production.

#### Integration of FBEDs for E-Textiles

FBEDs has been integrated into textiles by manual weaving at laboratory scale, as a conceptual E-textile devices. To boost production efficacy, there is a need for quasi-automatic or completely automatic production techniques. Currently, two integrating approaches have been applied to integrate the FBEDs into textiles:

1. The fabrication of the full FBEDs and integration of these electronic devices into textiles by conventional textile processes.
2. The fabrication of FBEDs and weaving them as anode and cathode electrodes with interlaced configuration to develop textile-based electronics.

**Fig. 20:**
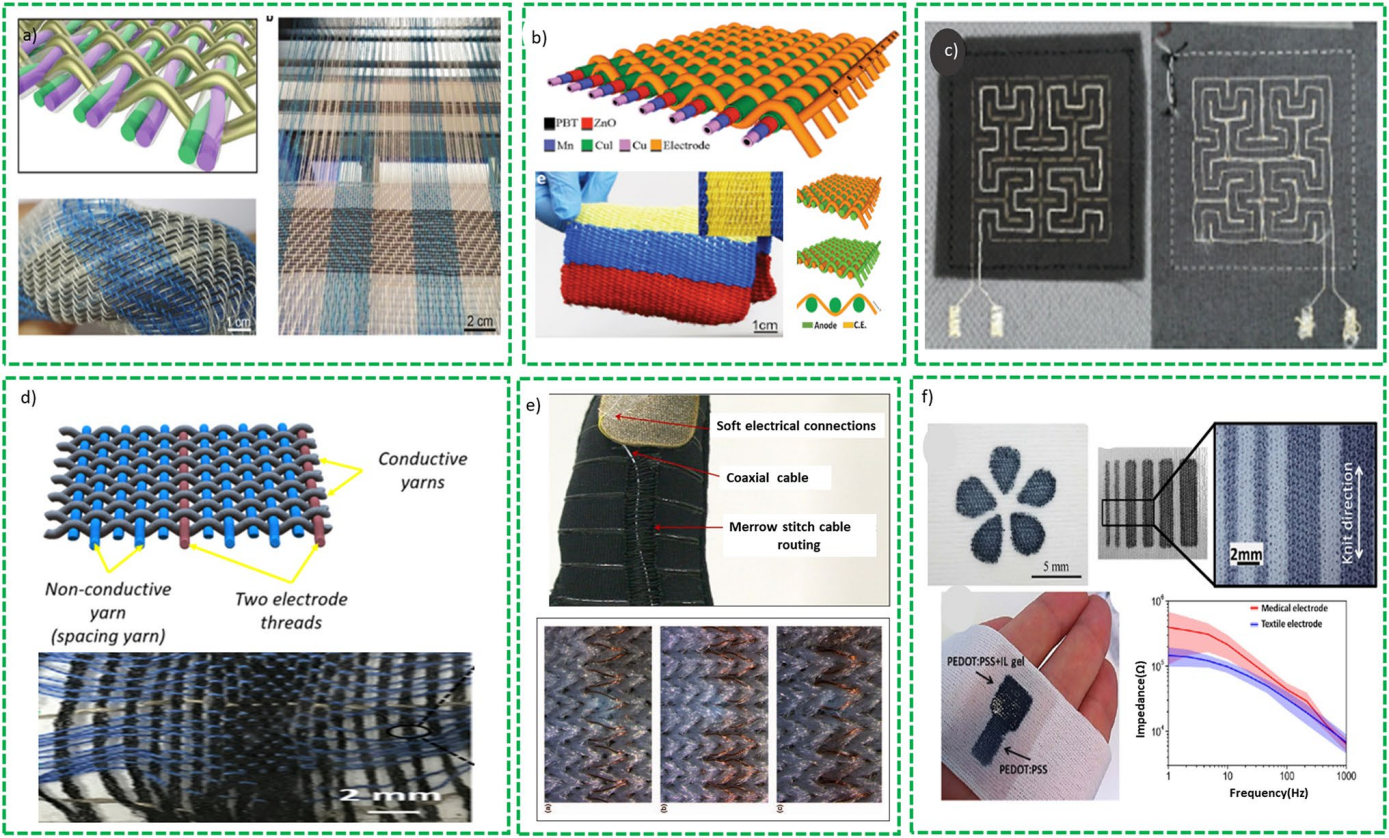
**a** Graphical representation of fiber-shaped lithium-ion battery. Reprinted with permission from Ref. [219]. **b** Schematic of photovoltaic textile and a pictorial of an as developed photovoltaic textile mixed with colored wool wires with anode and cathode. Reprinted with permission from Ref. [220]. **c** Pattern of conductive inks, reprinted with permission from Ref. [221]. **d**–**f** Conductive circuit on textile embedded in/on textile by different textile techniques, **d** reprinted with permission from Ref. [222]. **e** Knitting, reprinted with permission from Ref. [223]. **f** Embroidery, flower pattern conductive polymers on textile substrate. Reprinted with permission from Ref. [224]

The first approach has been used to fabricate diverse FBEDs such as flexible solar cells [25], flexible lithium-ion batteries [13, 155], flexible sensors [198, 199], and light-emitting diodes [203]. Figure 20a depicts fiber-shaped liquid electrolyte-based lithium-ion batteries fabricated into power fabrics [219]. The working potential windows of the device for flexible lithium-ion battery can be increased from 2.5, 5, 7.5 and 10 V, respectively. This potential window can be obtained after connecting electrodes in series and parallel configurations depending on the intent of usage. However, it is difficult and challenging to successfully link a substantial number of fiber electrodes of assembled FBEDs.

Compared to the first approach, the second approach is mainly exploited to fabricate textile substrate-based solar cells in which two constituents’ yarns (warp and weft) are used as the anode and cathode, respectively, as shown in Fig. 20b [220]. A textile-based polymer solar cells of defined scale of length can be weaved by traditional manufacturing looms. During the weaving process, changing parameters such as thickness of the film and distance between anode fibers were systematically studied. The conclusion was the thickness of the film and the larger distance between the anode fibers deteriorate the performance of textile based polymer solar cell energy harvesting device. These can also be connected in parallel or series according to requirement of current or voltage. The limitation of this approach is encapsulation of the device to prevent damage, which is a major challenge to date. On the other hand, if the complete electronic device is encapsulated, some key attributes may be compromised, such as moisture management and air permeability. More focused research is needed to address these issues in the seamless manufacturing of the interwoven configuration approach.

It is worth noting that the mechanical attributes of many FBEDs are inferior to expected after automatic integration. As an outcome, integration of the FSEDs is performed manually which not only increases the cost of fabrication but also compromise the bulk production opportunity of FBEDs. This approach of manual integration may be adequate for small scale, but this aspect needs solutions.

#### Incorporation of Conductive Traces into Fabrics

Conductive yarn network can be incorporated in the textile by four different established techniques such as, weaving [225], knitting [226], embroidery of conductive yarn [227] and conductive ink patterning [221] as shown in Fig. 20c–f. Traditional processes weaving and knitting are explored to produce an interlocked network system of conductive textiles.

##### Weaving Process

Conductive yarn traces can be incorporated by weaving processes into the textile. First, conductive yarn is developed and then, weaved into consistent framework of design which was possible either in warp or weft direction or at identified sites. In the past couple of decades, a variety of weaving processes and patterns of conductive traces have been successfully designed and incorporated into textiles [228]. Dhawan et al. [229] and Gaeul et al. [222] developed a textile-based electrical circuit interlacing configuration exploring conducting and non-conducting textile yarns (polyester (PE) and copper/steel wires) into complete fabric/textiles. In these textile-based electrical tracks, transmission of electrical signals is received by establishing effective electrical interlinks and detachment from the circuits. But there is a limitation of capacitive and inductive crosstalk’s, which is usually exacerbated due to long parallel constituent yarns in a weaved circuit. This short coming is handled by special fabrication configuration such as coaxial and twisting couple of copper wires or insulated conductive yarns [229]. A similar product was also developed known as PETEX which was weaved with PE monofilaments and separated copper yarn as depicted in Fig. 20d. In this textile, although circuit pattern is facile and realized by using traditional weaving processes which is only wired in vertical direction which restricted freedom of the circuit routing.

In contrast to the weaving technique, the knitting technique of producing textile fabrics offers more elasticity owing to its three-dimensional design and fabrication. Consequently, a stretchy conductive circuit with a more complicated random route can be developed employing the knitting technique. An elastic conductive circuit was exhibited by employing fine shielded metal conductor wire co-knitted into textile as depicted in Fig. 20e [223, 226] which showed an electrical resistance of 44 and 45 Ω, for 5000 cycles with an average stretch of 46%.

Another technique can also be employed for incorporation for electrical circuits in the development of fabrics. This technique offers the advantage that customization or modification can be incorporated in the fabric after designing of electrical circuits. The embroidery process can be integrated with traditional commercial embroidery procedures using computer-aided design (CAD) [230]. The circuit pattern of conductive yarn can be transferred by sewing on the textile substrates. However, suitable strains are crucial to permit needle exploitation of the yarn at the comparatively high rates in the sewing machine, while the technical properties of the conductive yarn are usually smaller than traditional yarn. Good sewability can be achieved with composite yarn combining conductive and non-conducting yarns. Metallic yarn is currently used for making track patterns manufactured by applying a computer numeric control embroidery machine, as shown in Fig. 20f. The intrinsic elasticity is, however, compromised due to the inelastic conductive thread [224].

Another method to transfer conductive tracks on the textile substrate is the patterning technique. Several established techniques such as screen printing and inkjet printing can accomplish the fabrication of conductive textiles [231]. These printed circuits are limited by the electrical conductivity of inks and still a challenge. The unevenness and permeability of the fabric that complicate the printing of uninterrupted and even coating of conductive textiles or the large strain sustained by textiles which enables cracks or peeling of the conductive coatings are the constraints to be addressed. Recently, textile permeable conducting coatings or inks have been designed and fabricated which shows excellent mechanical and electrical properties of for instance, 450% of elasticity and 0.06 Ω sq^−1^ [232].

#### Connecting Technologies

Physical soldiering, technical riveting, and chemical bonding are the main techniques explored to perform the electrical connection between the electrical circuits and electronic modules on textile substrates. The physical soldering/welding technique is the most used technique for soldering/connecting electronic devices on boards. For example, a micro-spotwelding platform was introduced to join stainless steel yarn to the lead of a chip [230]. Even though a high electrical conduction was achieved, it is too rigid and brittle for E-textiles. These brittle connections in the textiles can be acceptable in those textiles only where body movement is limited.

However, soldering or welding processes could not be applied, if heat and brittleness cause mechanical gripping such as folding, fastening and embroidery of electronic components [233]. Like the welding/soldering process, fabrication based on folding and stapling techniques are also not flexible and it will lead to breakage during wrinkles and bending. The flexible connection only achieved via an embroidered connection, while the electrical contact is untrustworthy.

Another method that can be employed for the connection of the electrical components is chemical bonding via conductive pastes. These pastes or conductive adhesives connect the electronic device components with textile tracks as shown in Fig. 20h [234]. The advantage of this process is the facile fabrication and structure, however, it may have some environmental impacts such as moisture, temperature and a significant influence on the connection features. The incorporation of encapsulation layers can solve this problem.

### Functional Integration of Electronic Textiles

Practical applications of E-textiles which contain various electronic components need a physical support of these substrates. However, the operational integration of electronic devices parts in E-textiles has demonstrated very restricted commercial realization so far. This is because the electrical connections of FBEDs are more complicated in E-textiles than in planer electronic devices. The real E-textile systems that perform data acquiring, evaluation and communication require consistency and high computational analysis which lag much behind from commercialization and practical applications. Numerous approaches have been adopted for execution of operational integration of E-textiles.

#### Integration on Single FBEDs

**Fig. 21:**
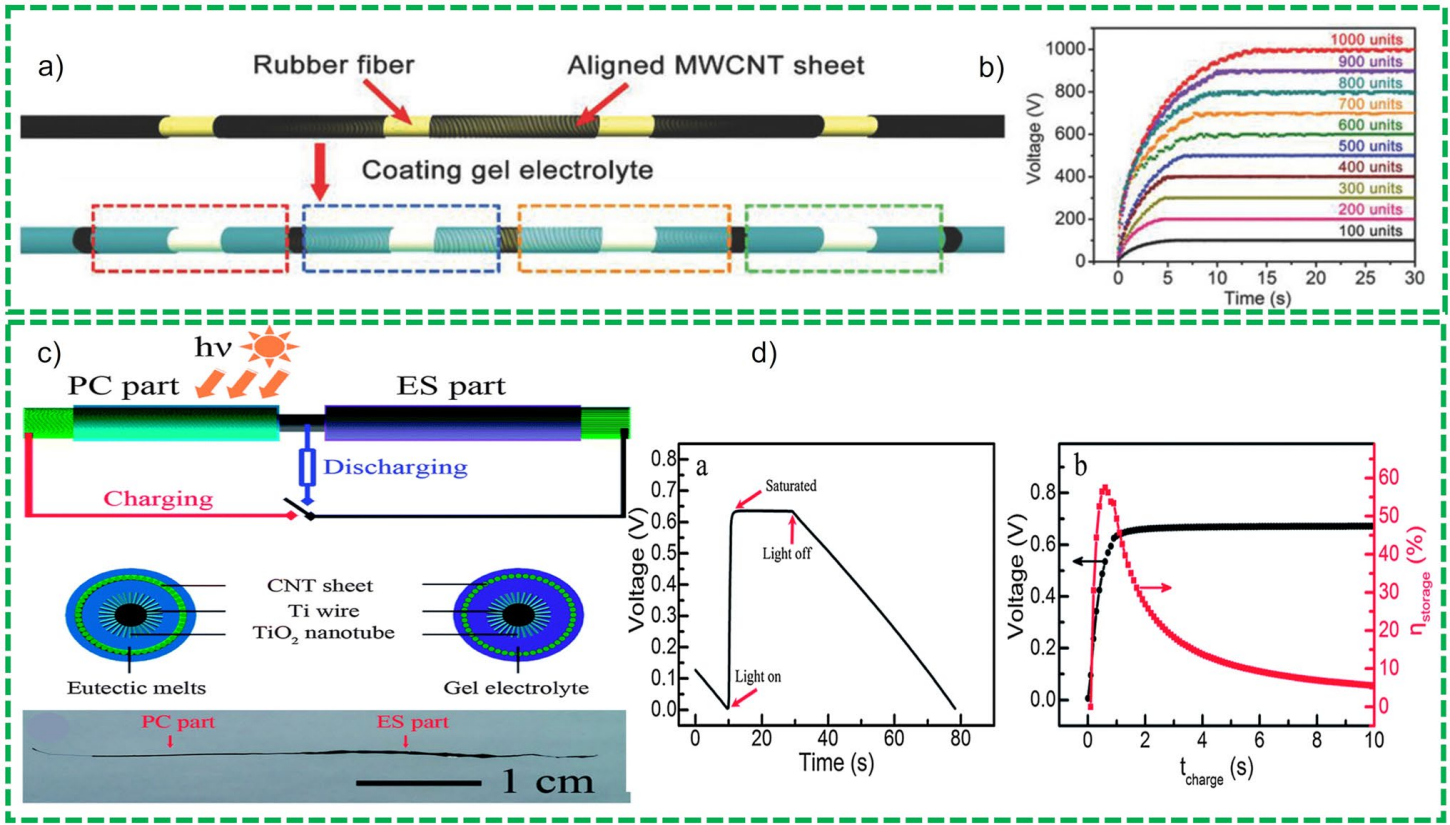
**a** Graphical representation of fiber-shaped SCs in series, **b** relationship between voltage on charging time. Reprinted with permission from Ref. [235]. **c** Pictorial demonstration of the structure of coaxially integrated photovoltaic device, along with cross-sectional views, **d** a standard photocharging and discharging of SC, relationship between voltage and storage efficiency on the solar charging time during charging and discharging process. Reprinted with permission from Ref. [236]

The convergence of several elements of an electronic device into one FBED not only eases the intricacy of peripheral circuits but also permits more appropriate and efficient devices. Selecting FSSCs as a benchmark example, conventional fabrication of the SCs devices has many disadvantages, especially, complex linkage procedures, bulkiness and fragile nodes which hinder the realization of practical E-textiles. To avoid the external connection wires, the structures of FBEDs are fabricated on single fiber with in-series electrical connections and offer higher efficiency as shown in Fig. 21a [235]. The integrated SCs device offered output voltage up to 1000 V along with excellent flexibility and stretchability as shown in Fig. 21b.

The integrated electronic and wearable devices in one fiber can harvest green solar energy into electrical energy and simultaneously stored it into storage devices such as SCs and batteries. For instance, an organic photovoltaic or dye-sensitized solar cell is integrated with SCs as demonstrated in Fig. 21c [206, 236]. These devices are fabricated, in which photoactive and electrochemical active materials were deposited onto a single fiber-based electrode and assembled into a device with suitable electrolyte. These left and right titanium wires were activated with TiO_2_ material which is used as common material for both solar cells as well as SCs device. Two multiwalled CNT-coated flexible electrodes are then coiled around both the electrodes to fabricate an integrated device. These devices were characterized by exposing DSSC to simulated illumination where DSSC harvested the energy and charged the SCs of 0.6 V in seconds [206]. Additionally, these devices fabrication in series configuration, the integration can be design into customized configurations in which SCs part is explored as core and energy harvesting part as the shell part [237].

The all-integrated electronic devices can further be integrated with some specific functional devices such as sensors to develop self-powered integrated systems. A CuInS_2_-based photodetector was fabricated for construction of a self-powered system which is integrated with coaxial configured SCs on single fiber substrate, as shown in Fig. 21c [238]. The device exhibited the same response powered by SCs part as compared to the devices powered by some external power source. The photodetector devices displayed excellent flexibility and bending stability with unchanged photo response. Some materials are more electrocapacitive and pulse responsive such as graphene, transition metal oxides and conductive polymer; therefore, it is valuable to fabricate integrated electronic device comprised of SCs and photodetection exploiting the same single electrode. For instance, a FSASCs-based integrated optoelectronic detection device was fabricated composed of Ti wire/Co_3_O_4_ nanowires as cathode and graphene/carbon fiber as anode which allow the assembly of devices of wider voltage window of 1.5 V as shown in Fig. 21d [239]. But when the SCs were charged up to 1.5 V, the seepage of current exhibited evident photo-response when the graphene or Co_3_O_4_ electrode was subjected to white light.

#### Integration on Fabric for E-Textiles

Ideally, to develop a functional E-textile, all the electronic parts are first fabricated in the shape of fibers and consequently, integrated for the final device. However, some FBEDs components such as transistors and memory are still in early stages and are far from real commercial applications. Currently, the first commercial electronic device was comprised of silicon-based electronic parts which were integrated into textile to realize the electronic functionalities such as data collection and analysis.

**Fig. 22:**
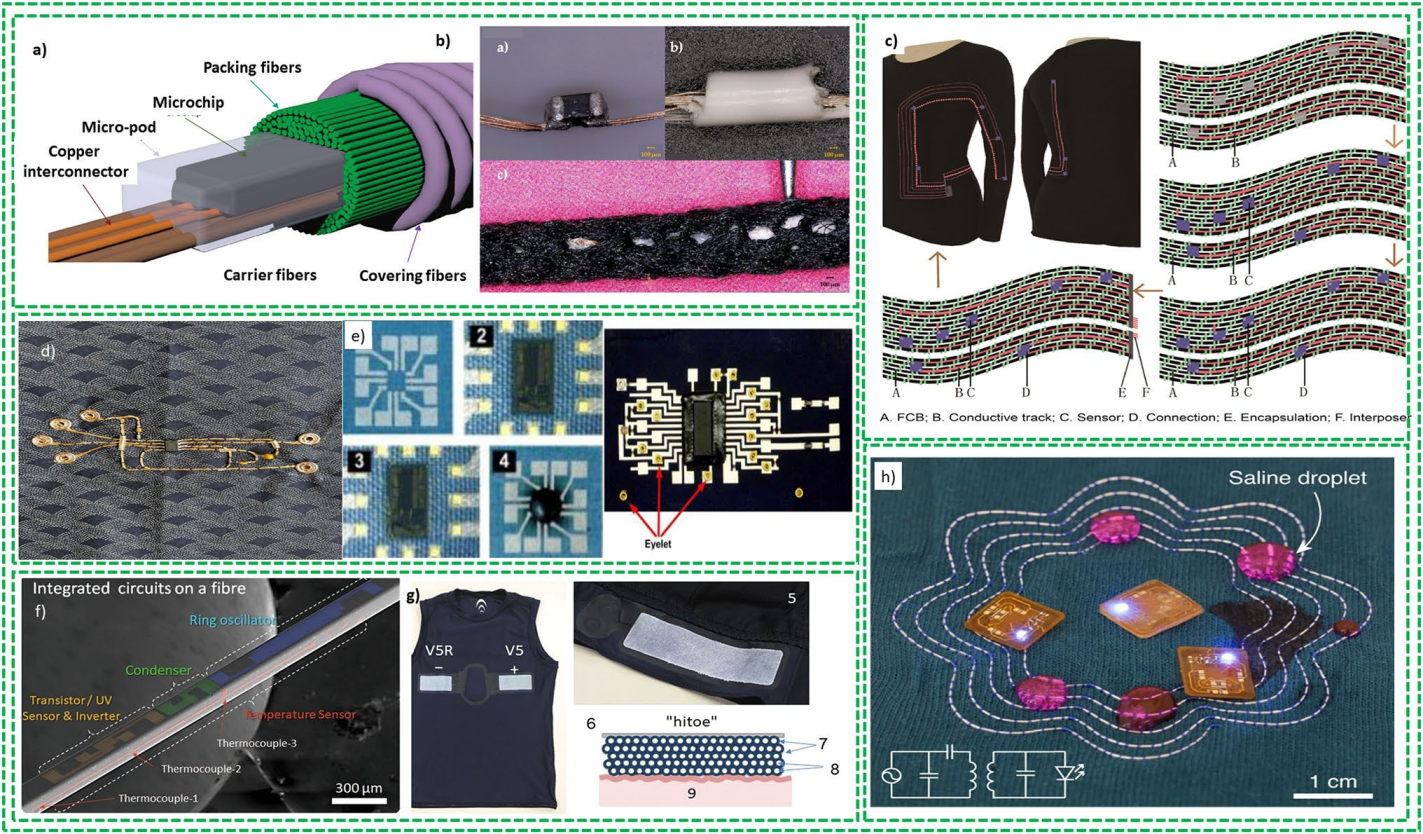
**a** and **b** Cross-sectional view of an electronically functional yarn, Photographs of prototype temperature-sensing yarn manufacturing process. An eight-strand copper wire was soldered onto the thermistor; the thermistor, copper wire and two polyester yarns were encapsulated with 9-20801 resin with 0.87 mm thickness; the final yarn with the position of the thermistor indicated by a needle. Reprinted with permission from Refs. [240, 241]. **c** Electronic tracks on textile substrate using interposers. Reprinted with permission from Ref. [225]. **d** and **e** Single-layered P-FCB system manufacturing process; Implementation of multi-layer connection using eyelet. Reprinted with permission from Ref. [242]. **f** SEM image of electronic devices fabricated on a single microfiber Reprinted with permission from Ref. [246]. **g** Designed smart ECG garment system. Reprinted with permission from Ref. [247]. **h** Embroidered conductive yarn circuit. Reprinted with permission from Ref. [246]

Over the period, different integrating strategies have been developed to integrate electronic parts into textiles. The typical strategy of discrete integration of electronic parts on textile substrate, in which rigid parts distributed textile surface, avoid large rigid area and allow natural distortion such a wrinkling [228]. Lately integrated electronics textiles are fabricated by inserting prepacked electronics into pockets, sewing comprising modules to the surface, integrating functional properties by exploiting conductive yarns, using screen printing technology or integrating electronic devices into belts or straps. The ultimate goals must be fabricating an integrated functional textile which offer distinct attributes such as softness, conformability, and flexibility. A novel approach was introduced by Nottingham University to encapsulate semiconductor chip within the yarn. In this approach, chips are connected with fine wires of copper and incorporated into yarn and protected by polymer coating as demonstrated in Fig. 22a and b [240–242]. Researchers earlier fabricated a circuit interface concept that embraced the field mismatch between the electrical elements and fabrics which consequently enables the integration of arbitrary elements as demonstrated in Fig. 22c [243]. Another technique was to design a planar circuit board directly produced by screen printing technology on flexible substrates to fabricate patterned electrodes for electronic parts integration as shown in Fig. 22d and e [242, 244]. Different electronic parts and components such as chip capacitors, resistors, and light-emitting diode (LEDs) can be coupled directly with flexible substrates as depicted in Fig. 22f [245].

The recent state-of-the-art technology for integration of flexible electronics components comprises the design of flexible electronics that can be directly woven into textile to obtain a fully wearable electronic device. Soldering technique is the first technique to integrate the flexible electronics components with textile in the form of conductive tracks, but it has some short comings such as rigidity and brittleness. Embroidery can also be exploited to integrate elements on flexible substrates in which conductive yarns are weaved to obtain an interlinked electronic module such as the ECG shirt demonstrated in Fig. 17g and h [245] exploiting embroidery technique [246, 247]. They exhibit excellent stability in terms of electric connections and can be enhanced by the device encapsulation. For commercialization and bulk production, standard snap fasteners are exploited which allows reproducible manual mounting and dismounting of electronic parts. Different brands of intelligent textiles have been fabricated and developed for real-time monitoring and access of the real-time data where these electrodes have been weaved into textiles which exhibited excellent washing resistance and can be easily integrated into textiles.

## Evolutionary Developments

The evolutionary trends of the current wearable market must be analyzed to appraise the fast expansion of this market and extent of research on fiber and textile-based electronics. The fiber and textile based electronics is an emerging field because of its promising functionalities related to the flexible electronics. The research and development tendencies adopted by scientific community can be evaluated by the number of publications in this field per annum here based on the Scopus and Web of Science archive. Several keywords employed for the research of scientific articles in relationship with fiber and textile based electronics, such as “intelligent textiles and clothing,” “textile electronics,” “wearables” and “fiber,” “wearable” and “textile” were scrutinized. From the analysis, it is observed that the number of articles and publications on textile electronics and wearables have been increased during the last decade. Similarly, the trends were also investigated through evaluating the number of patents registered in the same period and it has shown the same trend.

One of the best key performance indicators of growth of textile electronics is the economic impact and market worth of the textile and fiber-based electronics. The smart and intelligent textiles market is the major application area for electronic textiles, and it has been growing at a fast pace during the last couple of decades. According to one report, the global E-textiles and smart clothing market size is 1725.2 million USD in 2020 and it is increasing at compound annual growth rate (CAGR) above 32.3% and projected to be 15,018.9 million USD by 2028 [248]. According to one published report (Tractica report 2017), the number of shipments of smart clothing will increase from 1.7 million units in 2016 to 26.9 units of smart clothing by 2022 at a CAGR of 58.6% [249]. Currently, the classification of smart clothing includes smart shirts, undergarments, trousers, jackets, and shoes. Owing to different functionalities, they are classified as energy harvesting, energy storage and sensing which are employed in numerous application such as health monitoring, sports and fitness, defense, fashion, and industrial manufacturing. However, the share of smart cloth in the wearable electronics market is still very low, estimated to be 1% as compared to other electronics systems such as smart watches or wristbands. There are certain limitations for smart clothing to capture major shares in wearable markets such as high development cost, economy of scale, continuous power supply, washability, security, and comfortability which are major hindrances in commercialization of these products. Despite all these challenges, the smart clothing markets future is still exhilarating since clothing is essential part of the human being’s daily life. Total market worth of the clothing was 12.7 billion USD in 2018 and according to the forecast for 2018–2026, the value of this market is projected to be 19.6 billion USD in 2026 [250]. From these figures, the importance of textile and fiber electronics advancement can be imagined, and forecast shows an unprecedent market growth and expansion in next decade.

## Conclusion, Challenges, and Outlook

Fiber-based electronic devices are the key players in the realization of wearable E-textiles. Several research studies have been dedicated to developing a FBEDs with energy harvesting, energy storage, electronic, sensing functions. The current research and studies are focused on identification of functional requirements of FBEDs and assortment of active materials, device design, fabrication techniques and structure of the devices. Scientific community has surpassed way ahead of conducting fibers and fiber-based electrochemical sensors, and dielectrically gate field effect transistors have been replaced with complex logic gate from simple switches. The development of FBDSSCs and FBPSCs has opened new potential fields for solar energy harvesting and supplying this power to wearable devices. The recent trends and development in this field lead toward the development of self-reliant E-textile system, i.e., only fabricated with textile fibers and fabrics. The availability of diverse nature of fiber-based electronics components will eventually help to cater various needs of practical applications of fiber-based electronics and will stimulate further development of fully integrated E-textile systems.

In the recent past, fiber and textile-based electronic devices are deployed in various fields and considered the most attractive branch of modern electronics and have attracted significant attention from of researchers and enterprises. Even though, considerable growth has been made in the flexible electronic devices field, still only a few fiber and textile-based electronic devices are commercially available. The main fiber and textile-based electronic devices available in the market up to date are flexible fiber-shaped sensors. They are mainly deployed for electronic signal monitoring, but more functionalities are needed for fully function wearable device with diverse functionalities. There are still many challenges for fiber and textile-based electronics and also technology limitations which hinder their commercialization:

1. *Poor performance:* one of the major limitations of the fiber and textile-based electronic devices is the poor performance compared to its counterparts, i.e., rigid electronic devices and thin film devices. One of the main reasons for poor performance of the FBEDs is their thin shape which give lower conductivity as compared to plan electrodes. Numerous attempts have been made to synthesize and fabricate a high conductive material and optimize the configuration and structure of the devices without compromising their mechanical and electrical properties. This comes through a need for novel electronic materials for FBEDs, with enhanced performance for the electronic device’s applications. Further, the cyclic life of the FBEDs also needs to be improved to combine high performance and reduced cost owing to rapid change of the devices. The main problem of fabrication and characterization of these devices is the degradation of the electronic device due to active material loss or breakage of substrates during repeated measurement such as bending and stretchability characteristics. Stretchability is one of the benchmark characteristic for FBEDs; therefore, they can sustain mechanical stresses during stretchability and deformation. Unfortunately, stretchability is obtained at the cost of loss of conductivity due to incorporation of unused elastomeric polymer materials that enhance volume and make them bulky. Another problem with FSEDs is their sensitivity to atmospheric oxygen and moisture which cause deterioration of devices and hence their performance. Washing resistance is another challenging topic, and existing encapsulation packages and materials are unable to meet these challenges and requirements of the FSEDs with extremely curved exterior. It is particularly important for commercialization of the FBEDs that researcher focus on development of new encapsulation and packaging materials and fabrication techniques of FBEDs.
2. *Large-scale production techniques:* for economy of scale and large-scale production of the FSEDs, bulk production techniques are crucial and currently difficult to meet the requirements and targets. Continuous fabrication of the flexible electronic devices is quite challenging to obtain a stable interfacial bonding among active material and textile fibers. These devices are still designed and fabricated at laboratory scale manually, and there is no automatic procedure and technique for their production. Large-scale production compromises the performance of the FSEDs due to comparatively low electrical conductivity of flexible electrodes.
3. *High cost of production:* most significant concern for sustainability of any manufacturing industry to endure is the enhancement of product performance along with the reduced cost of production. High cost of production is also a major limitation for commercialization and mass production of E-textile owing to high cost of material and production technology. The future research focus will be finding stable and effective active material for electrode fabrication, electrolyte material along reduced cost.
4. *Energy storage of textiles:* although various research groups are working at laboratory scale, there remain many challenges for industrial scale production of E-textiles. But the energy storage capacity of the textile-based supercapacitors is still on lower side as compared to rigid traditional batteries. The most important challenge is the improvement of energy storage capability of the textile-based energy storage devices.
5. *Integration of electronic devices into textile:* the most important application of the FBEDs is smart clothing and electronic textile. At this front, it is essential to develop new fabrication techniques for the integration of different FBEDs into textiles with a safer and more relaxed human/textile interface model. Additionally, consistent linking methods and integrated tracks are needed to accomplish the high integration of E-textile. Currently, very few publications and studies are present in the literature about integration and human/textile interface is not considered in detail. Moreover, more functional fiber-based devices integration is significant to realize the broader spectrum of application of these textile electronics. For example, an integrated device in which solar cell, SCs and sensor are integrated into a single device, which can realize the monitoring of environmental alterations without using of peripheral energy supply. Even though, the integration of several functionalities into textile is well established, the disparity in material, assembly necessities among the various varieties of devices remain a crucial hurdle for functional applications.
6. *Multifunctionality:* E-textiles are a promising and fascinated technology that could soon become the essential part of everyday live owing to new concept of internet of things (IoT). For this reason, a textile-based electronic device should be diverse in applicability. However, E-textiles with single functionality limit its application. Fabrication of multiple functional textiles with multiple functionalities is challenging owing to integration of different electronic devices into textiles and will be focused of research in future.
7. *Joining technology reliability:* although several organizations are working on developing standards for E-textiles such as ASTM Subcommittee D13.50, IEC-TC 124, ISO-ISO/TC 38, etc., but currently no standard protocols are available to directly compare the joining techniques. There is no standard definition of reliability in E-textile but usually it involves bending endurance, sweat, washing resistance, stretching, humidity and temperature cycling. Different fields of application have different protocols and standards for reliability check, e.g., fashion garment can be hand washed whereas medical apparels need to resist machine washing at 90 °C.
8. *Safety issues:* along with the above-mentioned limitations and challenges, safety of the devices and its applications is also a major concern for fully developed wearable electronics and practical applications. Some FSEDs such as flexible batteries frequently need to use combustible and toxic organic electrolytes and they are vulnerable to risk of flames and bursts caused by short circuit during the bending. Durable and environmentally benign liquids and gel-polymer electrolytes are desired as an alternative to liquid organic electrolytes in FBEDs which must be tuned to enhance the electrochemical performance. Another safety concern issue is security of the personal data. Wearable sensors are expected to have a large database of personal data, where declassification of sensitive data may have detrimental impacts. Therefore, for the applications of these wearables, data security has to be the most crucial element. There are still some other problems that must be addressed such as comfortability.
9. *Washability:* E-textiles, like other everyday textile clothing, must have a washing resistance property to sustain general usability. Washability is considered one of the main challenge and obstacles to realize the commercial production of E-textile and wide-scale availability in the market. E-textiles suffer from poor washing resistance, which limit the reliability of the product and its commercialization. The hydrophilic nature of the textile substrate and capillary effect even hydrophobic substrates, still absorb water and making textile-based devices malicious. Moreover, application of mechanical stresses during washing operation may damage electrical connection of integrated textile devices and impedance of the device become uncontrollable. There washing of E-textiles remains a challenge, and there is a need of new technology that can provide better washing stability for conductive textiles.
10. *Standard evaluation systems:* One of the main issues with FBEDs is the lack of standardized performance evaluation practices that makes it challenging to compare the performance of the different electronic devices. Moreover, the reported performance formats are communicated in distinct forms according to various units. For instance, FSCs performance is reported in different units such as gravimetric, volumetric/areal/linear specific capacitance/specific energy/specific power and active mass/area/volume which are computed from single electrodes (three electrodes setup) or from whole devices (two electrode system). This creates confusion to compare performance in different units. Additionally, flexibility attributes such as bending, stretchability, twisting and other tolerances are distinct indicators of FBEDs, but there are no unified evaluation systems for FBEDs at present. There must be standard performance evaluation system and testing procedures in place for FBEDs to compare the performances of the FBEDs.
11. *Application of fiber and textile electronics:* use of fiber and electronics need the combined attempts of different disciplines such as chemists, textile engineers, material scientists, electrical engineers, and physicists. This multidisciplinary and interdisciplinary partnership will enhance the probability of effective implementation of fiber and textile applications in wearable electronics and offer numerous prospects to enhance human life standards.
12. *Sustainability manufacturing:* the goal of the E-textiles is to have seamless integration of electronic devices into textiles where electronic circuits remain undetectable by wearer. The accomplishment of E-textiles may have adverse effects for sustainability, as it makes renovation or recycling of an E-textiles more challenging. Some experts pointed out the risks related to large-scale production and mass markets which will introduce new waste stream of products whose recycling will be a challenge either E-textiles or electronic equipment. This makes issue of sustainability more important and more challenging about production of sustainable E-textiles.
